# Graph-Based Analysis of the Metabolic Exchanges between Two Co-Resident Intracellular Symbionts, *Baumannia cicadellinicola* and *Sulcia muelleri*, with Their Insect Host, *Homalodisca coagulata*

**DOI:** 10.1371/journal.pcbi.1000904

**Published:** 2010-09-02

**Authors:** Ludovic Cottret, Paulo Vieira Milreu, Vicente Acuña, Alberto Marchetti-Spaccamela, Leen Stougie, Hubert Charles, Marie-France Sagot

**Affiliations:** 1 INRA, UMR1089 Xénobiotiques, Toulouse, France; 2 Laboratoire de Biométrie et Biologie Evolutive, CNRS UMR5558, Université Lyon 1, Villeurbanne, France; 3 Bamboo Team, INRIA Grenoble-Rhône-Alpes, Montbonnot Saint-Martin, France; 4 Università di Roma “La Sapienza,” Rome, Italy; 5 Vrije Universiteit Amsterdam, Amsterdam, The Netherlands; 6 Centrum voor Wiskunde en Informatica, Amsterdam, The Netherlands; 7 INRA, INSA-Lyon, UMR203 BF2I, Biologie Fonctionnelle et Interactions, Université de Lyon, Villeurbanne, France; University of Virginia, United States of America

## Abstract

Endosymbiotic bacteria from different species can live inside cells of the same eukaryotic organism. Metabolic exchanges occur between host and bacteria but also between different endocytobionts. Since a complete genome annotation is available for both, we built the metabolic network of two endosymbiotic bacteria, *Sulcia muelleri* and *Baumannia cicadellinicola*, that live inside specific cells of the sharpshooter *Homalodisca coagulata* and studied the metabolic exchanges involving transfers of carbon atoms between the three. We automatically determined the set of metabolites potentially exogenously acquired (seeds) for both metabolic networks. We show that the number of seeds needed by both bacteria in the carbon metabolism is extremely reduced. Moreover, only three seeds are common to both metabolic networks, indicating that the complementarity of the two metabolisms is not only manifested in the metabolic capabilities of each bacterium, but also by their different use of the same environment. Furthermore, our results show that the carbon metabolism of *S. muelleri* may be completely independent of the metabolic network of *B. cicadellinicola*. On the contrary, the carbon metabolism of the latter appears dependent on the metabolism of *S. muelleri*, at least for two essential amino acids, threonine and lysine. Next, in order to define which subsets of seeds (precursor sets) are sufficient to produce the metabolites involved in a symbiotic function, we used a graph-based method, PITUFO, that we recently developed. Our results highly refine our knowledge about the complementarity between the metabolisms of the two bacteria and their host. We thus indicate seeds that appear obligatory in the synthesis of metabolites are involved in the symbiotic function. Our results suggest both *B. cicadellinicola* and *S. muelleri* may be completely independent of the metabolites provided by the co-resident endocytobiont to produce the carbon backbone of the metabolites provided to the symbiotic system (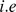., thr and lys are only exploited by *B. cicadellinicola* to produce its proteins).

## Author Summary

Some bacteria, called endocytobionts, permanently live inside the cells of a pluricellular organism and often bring an adaptative advantage to their host by providing compounds that the latter cannot produce or find in its diet. The association may involve several species of bacteria within the same host. The sap-feeding insect called glassy-winged sharpshooter thus maintains a permanent metabolic association with two different species of bacteria that it hosts within specialised cells. Complete genome annotations of the two endocytobionts allowed to reconstruction of their metabolism. By manually inspecting those annotations and comparing them to reference metabolic functions, earlier studies revealed a great complementarity between the metabolisms of the two endocytobionts and indicated potential metabolic exchanges between them. However, the metabolism of an organism is complex enough that such an approach could only give a partial description of the metabolic exchanges in the symbiotic system. We therefore determined all the metabolic exchanges in the symbiotic system by a systematic and automatic exploration of the full metabolism of the two endocytobionts in order to detail those leading to the biosynthesis of compounds involved in the symbiotic function of each bacterium. Our results highly refine our knowledge about the complementarity and the connections between the metabolisms of the two bacteria and their host.

## Introduction

Intracellular symbiosis involves a unicellular organism (the endocytobiont) which durably lives inside the cells of the other partner (the host). In the last century, the crucial role of intracellular symbiosis in the ecology and evolution of many eukaryotes was many times demonstrated [1], [2].

Intracellular mutualism (where the presence of the endocytobiont increases the fitness of both host and endocytobiont) was particularly well described in several associations between insects and bacteria [3], [4]. The association is most often metabolic: each partner provides metabolites that the other one cannot produce nor find in its environment. The complete genome annotation of mutualistic endocytobionts associated with insects revealed for all of them an extreme genome reduction paired with an extreme metabolism reduction [5]. Many metabolic functions of the bacterium are thus provided by the host and, inversely, the metabolism of the endocytobiont appears specialised into functions that are absent in the metabolism of the host. In addition, it often occurs that a host provides a habitat for more than one mutualistic intracellular bacterium species. This is the case for instance of the sharpshooter (*Homalodisca coagulata*) which hosts two bacteria: the 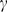-proteobacterium *Baumannia cicadellinicola* and the Bacteroidetes *Sulcia muelleri*. The complete genome annotation of the two endocytobionts revealed that their metabolic capacities are broadly complementary [6], [7]. The metabolism of *B. cicadellinicola* is globally devoted to cofactor and vitamin biosynthesis whereas the metabolism of *S. muelleri* is specialised in the essential amino acid biosynthesis that the sharpshooter cannot produce nor find in its diet, the xylem sap. Nevertheless, the partition of these metabolic roles is not so perfect: *B. cicadellinicola* produces two essential amino acids, methionine and histidine, that *S. muelleri* cannot produce while the latter appears to be able to synthesise menaquinone, a vitamin. Moreover, the complementarity between the two metabolisms also concerns the biosynthesis of some metabolites not needed by the insect host, such as the fatty acid biosynthesis pathway, supplied by *B. cicadellinicola*, except for one step which is provided by *S. muelleri* [7].

However, these previous analyses were essentially manually performed by comparing the lists of annotated metabolic genes using as reference the metabolic pathways available in metabolic databases such as KEGG [8] or MetaCyc [9]. Even when highly reduced, a metabolic network is however complex enough that such an approach based on lists of genes and metabolic pathways could only give a partial description of the metabolic exchanges in the symbiotic system, even for those directly involved in the symbiotic functions of the endocytobionts.

The aim of this study was therefore to determine the possible metabolic exchanges in the symbiotic system by a systematic and automatic exploration of the full metabolic networks of the two endocytobionts in order to detail those leading to the biosynthesis of metabolites involved in the symbiotic function of each bacterium.

Defining in an exhaustive way the metabolic exchanges in a symbiotic system implies to be able to indicate all the metabolites needed by one partner and produced by another partner. Our first task was thus to identify for each endocytobiont the metabolites potentially imported from the host cell (that is, the so-called “seeds”) and produced by another partner.

Focusing on the biosynthesis of the specific compounds that each bacterium produces and provides to the symbiotic system (from now on, we denote such compounds by “targets”) then necessitates to determine which sets of exchanged metabolites lead to their production. Our second task was thus to identify for each endocytobiont the subsets of seeds (from now on, we denote such subsets by “precursor sets”) that are sufficient to produce the targets, and to identify them all, that is all alternative precursor sets for each target.

These two tasks, and particularly the second one, are hardly feasible by just manually inspecting the metabolic pathways inferred from genomic annotations. Such broad and systematic analyses require working with the full metabolic network to consider it in a systemic way.

An intuitive way to define the seeds of a metabolic network is to consider as nutrient a metabolite not produced by any reaction but consumed by one or several ones. However, in particular because of reversible reactions that produce nutrients, this definition is not sufficient. Borenstein *et al.* extended the seed definition in a metabolic network by decomposing the metabolic graph into strongly connected components and then detecting those without incoming edge [10].

Whatever way is adopted to define the sources of a metabolic network, the next question is to determine which precursor sets are able to produce the targets. Romero *et al.* (2001) proposed a method returning alternative precursor sets for a set of target compounds [11]. Their algorithm was based on a backtrack traversing of the metabolic graph from the target compounds to the seeds. Unfortunately, how cycles are dealt with during backtracking is not described in the method.

Another method proposed by Handorf *et al.* to find precursor sets is based on a forward traversing of the metabolic graph [12]. Their algorithm is based on the concept of scope defined as the set of compounds that a set of initial seeds is able to produce [13]. The way to find precursors proposed by Handorf *et al.* is then to test the reachability of several sets of seeds heuristically defined [12]. However, the method does not take into account cycles that may appear between the seeds (see Methods) and only provides a subpart of the possible precursor sets for a target compound.

Recently, we proposed the first definition of minimal precursor sets that explicitly addresses the problem of cycles and the first exact method to find them [14]. In addition, our method, called PITUFO (for “Precursor Identification To U For Observation”), is able to deal with any definition of seeds.

**Figure 1:**
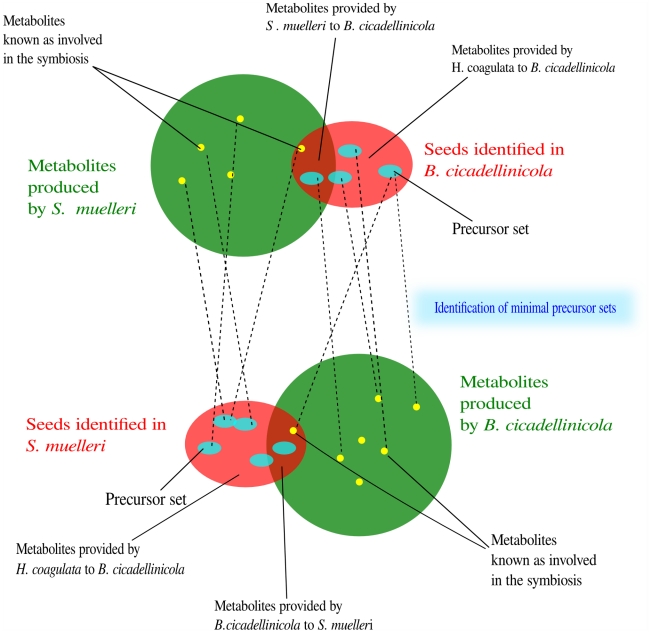
Identification of the metabolic exchanges in the symbiotic system involving the two endocytobionts *B. cicadellinicola*, *S. muelleri* and their insect host, the sharpshooter *Homalodisca coagulata*. Seeds are identified in the metabolic network of each bacterium (red sets). By comparing with the metabolites produced in the metabolic network of the other bacterium (green sets), we determined which metabolites are potentially provided by the cosymbiont or by the insect host. From the identified seeds, we determined which sets of seeds (precursor sets, blue sets) lead to the synthesis of metabolites important in the symbiotic association (yellow points).

To explore the metabolic exchanges occurring between *B. cicadellinicola* and *S. muelleri*, we first defined the set of seeds for each bacterium thanks to the method developed by Borenstein *et al.* [10]. By comparing with the compounds produced by each metabolic network, we were able to discriminate between the seeds produced by the co-endocytobionts and those potentially produced by the insect host or found in its diet. We then applied PITUFO to determine which subsets of seeds are involved in the biosynthesis of compounds already known to participate in the symbiotic function of each bacterium. The two steps are summarised in Figure 1.

Our study offers the first detailed and systematic description of the metabolic exchanges occurring in a symbiotic system. Our results also demonstrate the usefulness of graph-based dedicated methods in the metabolic analysis of multi-species systems.

## Methods

### Reconstruction of the metabolic networks

Draft metabolic reconstructions for the bacterial genomes of *Baumannia cicadellinicola* and *Sulcia muelleri* were downloaded from the MaGe annotation system [15]. This platform makes available metabolic networks built from re-annotated genomes. Each metabolic network reconstruction is available in the pathway-tools format [16]. We restricted the networks to the small-molecule metabolism, meaning that reactions involving macromolecules such as nucleic acids or proteins were removed from the final metabolic networks.

A first manual curation consisted in removing what may be considered as “fake” reactions. Indeed, an enzyme is potentially able to catalyse several reactions but a limited number of them actually takes place in a given organism. The reactions that clearly do not happen correspond to those that either are disconnected from the network (they use as inputs compounds that are not produced in the organism and produce compounds not used as substrates by other reactions), or that are connected to the network only by cofactors. The topology of the network provides thus a clue to remove 14 reactions from the metabolic network of *S. muelleri* and 37 reactions from the metabolic network of *B. cicadellinicola* (see Tables S1 and S2). Such reactions can be automatically detected by a topological analysis but their elimination requires a manual inspection and some biological a priori since some reactions appear disconnected because of a hole in the network, that is, of a single reaction that is missing due to an error or incompleteness in the annotation process. The main clue to fill such holes is to inspect the completeness of the metabolic pathways predicted in the organism. By comparing these predictions with data from the literature, we are able to complete some metabolic pathways, and thus the metabolic network.

In addition, several reactions involve generic compounds (for instance, an aldehyde) in the draft metabolic networks. When the same reaction existed with specific compounds, the generic reaction was simply removed. This is the case of nine reactions in the metabolic network of *B. cicadellinicola*. If specific reactions do not exist, the reaction has to be duplicated into several reactions so that they involve compounds already existing in the metabolic network. This is the case of the reaction RXN-8972 for which a substrate is “lysine or meso-diaminopimelate”. This reaction was thus splitted into two reactions (RXN-8972BIS and RXN-8972TER in Table S4) that involve lysine and meso-diaminopimelate respectively.

In automatic metabolic reconstructions, several reactions can be assigned to annotated enzymes. These reactions often use the same main substrates but different cofactors or even completely different substrates. When several reactions were assigned to a same enzyme, we removed the reactions for which the substrates required were absent in the metabolic network. This is the case of eight reactions in the metabolic network of *S. muelleri* and of 19 reactions in the metabolic network of *B. cicadellinicola*. We removed also six reactions in the metabolic network of *S. muelleri* and seven reactions in the metabolic network of *B. cicadellinicola* that were classified into the small molecule metabolism whereas they clearly involve macromolecules (see Tables S1 and S2).

In the end, a total of 16 reactions in the metabolic network of *S. muelleri* and 58 reactions in the metabolic network of *B. cicadellinicola* were thus removed, using the clues mentioned above (see Tables S1 and S2).

The gene (*epd*) that catalyses the production of erythronate-phosphate from erythrose-phosphate appears as absent in the genome of *B. cicadellinicola*. Wu *et al.* made the assumption that this role could be carried by glyceraldehyde 3-phosphate dehydrogenase [6]. We thus added this reaction to the metabolic network of *B. cicadellinicola* (ERYTH4PDEHYDROG-RXN in Table S4).

McCutcheon *et al.* mentioned that no gene in *S. muelleri* could be assigned as *argE* or *dapE*, potentially coding for an enzyme catalysing one step in either the lysine biosynthesis and the arginine biosynthesis [7]. Interestingly, a gene (SMGWSS-116) was assigned as *argE* in the genome of *S. muelleri* by the MaGe annotation system.

We then considered the corresponding reactions in the two metabolic pathways as present (SUCCDIAMINOPIMDESUCC-RXN and ACETYLORNDEACET-RXN in Table S3).

**Figure 2:**
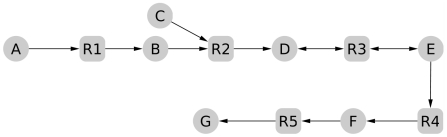
Example of a topological clue to assign a direction to a reaction. All the reactions except the third one (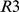) are indicated in the same direction. To produce 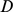 from 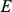 by 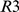, 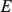 has to be acquired from the environment while there is no other reaction that produces it. Sometimes, biological clues allow to reasonably reject this hypothesis.

The direction of the reactions was first assigned based on the pathways where they are involved in the MetaCyc database [9]. A reaction is thus assigned as irreversible if it occurs in the same direction in all the MetaCyc pathways. If it was not possible to infer a unique direction, then the reaction remained reversible. The direction of most of the reactions in both metabolic networks were assigned in this way (Tables S3 and S4). Various manual corrections were also performed, essentially based on the constraints brought by the topology of the network and the biology of the organism, exemplified in Figure 2. For instance, the molecular weight of compound 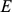 in Figure 2 could be too large to allow a transport of the molecule. Furthermore, the classification of the reactions that are in the same metabolic pathway where 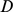 and 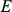 classically appear as intermediate metabolites can be an additional clue to assign the direction of 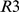. Eight reactions in the metabolic network of *S. muelleri* and 28 reactions in the metabolic network of *B. cicadellinicola* were hence assigned as irreversible (Tables S3 and S4).

The whole set of reactions of the metabolic networks of *S. muelleri* and of *B. cicadellinicola* are displayed in Tables S3 and S4. The metabolic networks of *S. muelleri* and of *B. cicadellinicola* are available in SBML format [17] in Datasets S1 and S2.

### Metabolic network filtering

We restricted our study to the metabolism involving transfers of carbon atoms between molecules. In each reaction, we thus removed sets of molecules that do not participate into carbon exchanges. We called these metabolites “side compounds”.

First, we established a list of 24 classical transformations between side compounds (*e.g.* 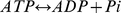) present in the metabolic networks of the two bacteria (see Table S5). When one of these transformations is identified in a reaction, the corresponding side compounds were removed from the reaction. Since whether metabolites are side-compounds in a given reaction is not always clear, some reactions were then manually corrected.

The following inorganic compounds were also removed: water, proton, phosphate, diphosphate, ammonia, hydrogen peroxyde, sulfite, sulfate and oxygen. Reactions that do not imply a transfer of carbon atoms are also eliminated. This is for instance the case of the reactions involved in the sulfate reduction.

The filtered metabolites are written in non-bold in Tables S3 and S4. The filtered metabolic networks of *S. muelleri* and of *B. cicadellinicola* are available in SBML format [17] in Datasets S3 and S4.

### Identification of the seeds

In order to describe the metabolic exchanges between the endocytobionts, the first step consisted in identifying which metabolites each bacterium potentially acquires from its environment. For this, we based ourselves on the definition of Borenstein *et al.* of the seed set of a network: “the minimal subset of the occurring compounds that cannot be synthesized from other compounds in the network (and hence are exogenously acquired)” [10].

To apply the Borenstein method to identify the seed sets, the metabolic network of each bacterium was modelled as a directed compound graph. In such a graph, nodes represent compounds and there is an arc between two compound nodes if at least one reaction produces one of the compounds (possibly more) from the other (possibly more). A reversible reaction between two metabolites is modelled by two arcs with opposite directions linking them. Since the side compounds were previously filtered (see previous Section), we avoid paths between metabolites that are biologically meaningless for our study.

The seeds identification is based on the detection of the strongly connected components (SCC) in the compound graph. An SCC is a subgraph 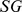 that contains a maximal set of nodes such that for any pair of nodes 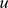 and 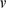 in 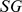, there exists a path between 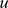 and 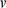 and a path between 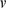 and 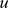. An SCC with no incoming arc is called a source component. Any compound inside a source component is a potential seed or, in our case, just seeds.

This definition of seeds allows to take into account the uncertainty about the direction of some peripheral reactions and about the presence of reactions producing metabolites that are actually exogenously acquired. We invite the reader to refer to the paper of Borenstein *et al.* for more precisions [10].

For each symbiont, we further inspect the collection of seed sets identified in order to classify these seeds as potentially provided by the insect or by the other co-symbiont. This is done by analysing the feeding source of the host as described in the literature [6], [7] or the metabolic network of the other co-symbiont to check whether these metabolites may be produced, and may therefore be supplied.

We implemented a version of the Borenstein's method using the Igraph package [18] and applied it to the compound graph of each bacterium.

### Identification of the precursor sets

Once the set of seeds was defined for each metabolic network, the next step was to identify which subsets of the seeds, henceforward called “precursor sets”, are sufficient to produce the metabolites known to be involved in the symbiotic metabolic association, that from now on we call the “targets”. Those are metabolites output by one bacterium that may then be used by the other co-symbiont or the host. We first put as targets the metabolites reported as involved in the symbiotic association by McCutcheon *et al.* [7]. We then added erythrose-4-phosphate, phosphoenolpyruvate, oxaloacetate and ribose-5-phosphate to the list of target compounds for *B. cicadellinicola* because of their presence both in its metabolic network and in the precursor sets identified for *S. muelleri*. These additional targets are particularly interesting since they could directly correspond to metabolic pathways shared between the two metabolic networks. For the same reasons, we added homoserine and 2-ketovaline to the list of target compounds for *S. muelleri*.

To identify the precursor sets, we used the PITUFO method that we recently developed [14]. Given as input a metabolic network, a list of seeds and a set of target metabolites, PITUFO returns the list of all minimal precursor sets for the target metabolites. For the purposes of this paper, we consider single sets of target metabolites, that is sets with only one element. Notice that once we get as result all minimal sets of precursors that are able to produce a target, we are covering all alternative paths that may lead to its production. understand the reasoning.

The strength of PITUFO comes from the fact that it takes into account cycles in the definition of precursor sets in a fully formalised manner. This allows to find paths from the precursor sets to the targets that pass through cycles in the network but are still feasible. Previous methods, such as those that compute the scope of a subset of the seeds as defined by Handorf *et al.* [13] and were later used to test the reachability of a target compound from a set of seeds [12], fail to link some sets of seeds to a target compound if there is such a cycle in the paths between them. The scope of an initial seed set 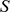 is 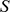 itself and then any metabolite that can be produced using only substrates already in 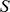 and added to it until no new compound can be produced [13]. This iterative process is called forward propagation [11] or network expansion [13].

**Figure 3:**
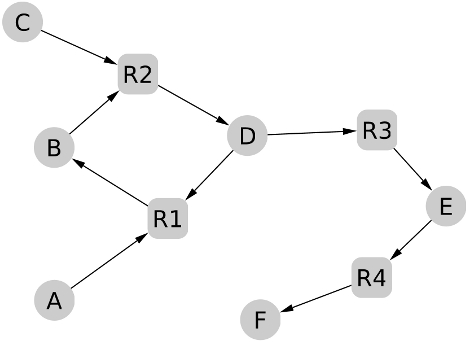
Example of cycle processing in the PITUFO algorithm. The metabolic network contains six metabolites 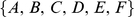 and four reactions 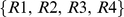. If 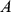 and 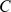 are indicated as seeds, the set 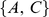 is considered as a precursor set of 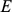 by PITUFO because the scope of 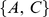 allowing the use of 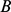 (or 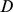) in the forward propagation process contains 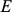 and also 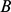 (or 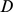).

The strategy of PITUFO to deal with cycles is to allow the use of metabolites involved in cycles if they are also produced (regenerated) in the forward propagation from the seeds to the targets. Indeed, in Figure 3, the scope of the set 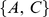 does not contain 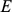 but if we allow the use of 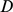 or 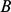 in the forward propagation process, then the scope of 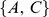 contains 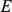. However, this could lead to clearly unrealistic paths without the constraint of regeneration of the compounds involved in cycles. For instance, in the same figure, if we allow the use of 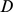 (and possibly also 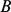), the scope of 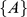 contains also 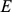 but uses up all of 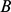 and 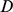 unless both were in infinite supply, in which case they should be considered as seeds.

The metabolites 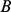 or 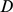, inside a cycle in Figure 3, that may be used and that are regenerated when the network is fired from a subset of the seeds (the set 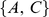 in the figure), are called “self-generating metabolites” [14]. Observe that these are defined in relation to a subset of the seeds. They do not need to be given as input but will be identified by the algorithm together with the sought precursor sets.

The following definitions allowed us then to formally establish what is a precursor set of a given target compound: a subset 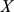 of the set of seeds 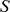 is considered as the precursor set of a target compound 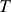 if there exists a set of metabolites 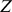 such that the scope of 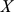, allowing the use of 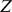 in the forward propagation process, contains 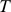 and all the metabolites in 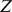, which ensures the regeneration of 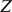. The set 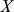 is considered as a minimal precursor set if there is no set 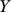 strictly contained in 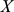 that verifies this property.

In the above definition (and in [14]), a metabolic network is considered as an hypergraph. Nodes are metabolites and there is an hyperarc between two sets of metabolites if there is a reaction that produces one of the sets from the other. Contrary to simple graphs where the nodes represent either compounds or reactions and arcs link individual nodes, the topology of an hypergraph takes into account the need in general for more than one substrate to activate a reaction [19].

The use of hypergraph modelling allows the formalisation of hyperpaths between precursor sets and targets since when a reaction is modelled as an hyperarc, it already explicitly establishes that all of its substrates are needed. On the other hand, an hypergraph could lead to some confusion for the method previously applied to identify the seed sets and proposed by Borenstein *et al.* [10] since there is no clear definition of strongly connected components of an hypergraph. For instance, isolated vertices may not be considered as SCCs and there is no unique definition of cycles in hypergraphs. For these reasons, the method was applied as in the original work on a compound graph representation of the metabolic network.

Since PITUFO is an exact method, it is enough to describe its input and output without recalling how the second is produced from the first. For those interested in the method itself, the algorithm is described in detail in [14].

The current version of the algorithm was implemented in Java and takes as parameter an SBML file describing a metabolic network [17] and a file containing a list of seeds and one or a list of target compounds.

The reconstructed metabolic networks complete or filtered are available in the Supplementary material. The method used is available at this address: http://sites.google.com/site/pitufosoftware/.

### Reconstruction of the sub-networks linking the precursor sets and the target metabolites

Figures S1 to S20 display the sub-networks linking each target metabolite to their precursor sets. These reconstructions were performed from the PITUFO results using the visualisation software Cytoscape [20].

## Results

### Global properties of the metabolic networks

Table 1 shows the number of reactions and compounds in each metabolic network as indicated in the Reconstruction Section. As mentioned in previous studies, both metabolic networks are extremely reduced. The metabolic network of *B. cicadellinicola* is less than half the size of the metabolic network of the free bacterium *Escherichia coli*. The reduction is even more important in *S. muelleri* since, with only 64 reactions, its metabolic network is less than ten percent the size of the network of *E. coli*. In both cases, the extensive manual curation allowed to highly reduce the number of reversible reactions as we succeeded to assign a direction to most of the reactions in the two metabolic networks.

**Table 1:** Number of metabolites, reactions, reversible reactions and identified seeds in the metabolic networks of *B. cicadellinicola* and *S. muelleri*.

	Nb metabolites	Nb reactions	Nb rev. reactions	Nb seeds
***B. cicadellinicola***	227	220	10	19
***S. muelleri***	74	64	1	10

### Identification of the set of seeds

**Figure 4:**
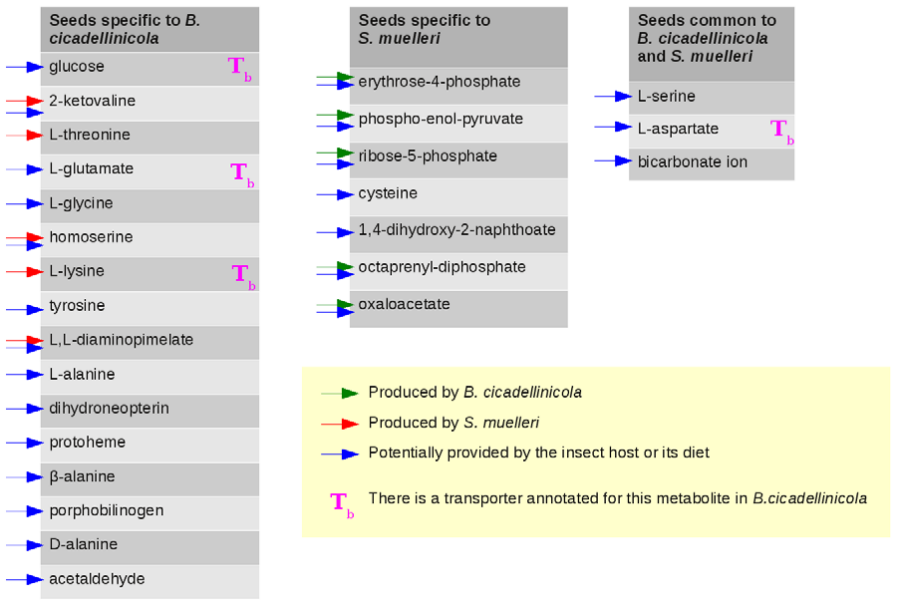
Seeds identified in the metabolic graphs of *B. cicadellinicola* and *S. muelleri*.

Figure 4 displays the set of seeds identified in the metabolic network for each bacterium. Coloured arrows mark those produced in the metabolic network of the co-endocytobiont and those potentially provided by the insect host according to the literature. Seeds that correspond to annotated transport reactions are also tagged.

We recall that the reactions involving big molecules are not taken into account in this analysis. For instance, the reactions charging amino acids onto their corresponding tRNAs do not appear in the metabolic network we built. This means two things. First, an amino acid involved only in the production of proteins does not appear in the seeds that we identified, which explains the absence of some essential amino acids in the set of seeds identified in *B. cicadellinicola*. Second, this also means that an amino acid identified as a seed is involved in the small molecule metabolism and not only in the production of proteins.

Moreover, we focused on the transfers of carbon atoms. There are then no inorganic metabolites in the sets of seeds. Furthermore, the organic compounds not involved in carbon atom transfers do not appear in this list. This is the case for instance of glutamine that appears as a source of nitrogen but not of carbon in the metabolism of *B. cicadellinicola*. This metabolite is thus a seed in the original metabolic network of the bacterium but not in the filtered one.

We identified 19 seeds in the metabolic graph of *B. cicadellinicola* and 10 seeds in the metabolic graph of *S. muelleri*. Only three seeds are common to the two sets: serine, aspartate and bicarbonate ion.

Whereas none of the seeds in *S. muelleri* could be linked to a transport reaction, four seeds (lysine, glutamate, aspartate and glucose) correspond to transport reactions annotated in *B. cicadellinicola*. Furthermore, a general amino acid ABC transporter annotated in its genome enables *B. cicadellinicola* to import also other amino acids identified as seeds: threonine, glycine, tyrosine and alanine.

Among the 10 seeds identified in *S. muelleri*, three are amino acids (cysteine, aspartate and serine) and three are sugars: erythrose-4-phosphate and ribose-5-phosphate are classically produced by the pentose phosphate pathway and ribose-5-phosphate by the glycolysis pathway.

Among the 19 seeds identified in *B. cicadellinicola*, we found 13 amino acids or related metabolites (such as homoserine or 2-ketovaline) and only one sugar, glucose.

**Figure 5:**
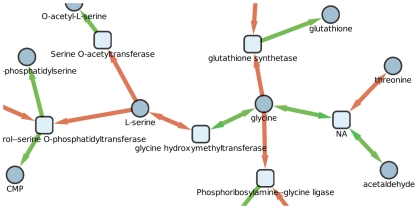
Part of the filtered metabolic network of *B. cicadellinicola* where are figured the two reversible reactions linking glycine, serine and threonine. The three metabolites are identified as seeds by the Borenstein method [10]. Squares correspond to reactions and circles to metabolites. The colour of the arcs differenciates the two sides of a reaction.

In the compound graph of *B. cicadellinicola*, serine belongs to the same source component (see Methods) as threonine and glycine. In fact, there are two reversible reactions that, respectively, link serine and threonine to glycine (Figure 5). It is thus impossible to distinguish which one(s) actually produces the other(s). All were considered as potential seeds and were taken into account by the PITUFO method for detection of the precursor sets.

**Figure 6:**
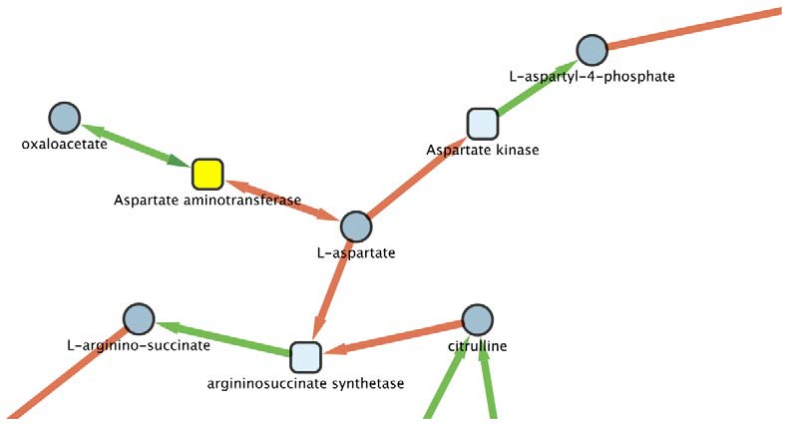
Part of the filtered metabolic network of *S. muelleri* where is figured the reversible reaction linking oxaloacetate and aspartate. Both metabolites are identified as seeds by the Borenstein method [10]. Squares correspond to reactions and circles to metabolites. The colour of the arcs differenciates the two sides of a reaction.

There is only one other example of such alternative seeds detected by the method of Borenstein [10]: these are oxaloacetate and aspartate linked by the same reversible reaction in the metabolic network of *S. muelleri* (Figure 6). All the other seeds found are metabolites that are not produced by any reaction.

Among the 16 seeds specific to *B. cicadellinicola*, five are produced by *S. muelleri* and two are certainly not provided by the insect host: threonine and lysine. Five seeds were already mentioned as potentially provided by the sharpshooter: glucose 6-phosphate, tyrosine, glycine, glutamate and alanine. Protoheme and porphobilinogen were mentioned by Wu *et al.* as needed to be imported by *B. cicadellinicola* to complete the siroheme biosynthesis pathway [6]. They seem not to be produced by *S. muelleri* and should be provided instead by the insect.

Five seeds identified in the metabolic graph of *S. muelleri* are produced by *B. cicadellinicola*: erythrose-4-phosphate, phosphoenolpyruvate, ribose-5-phosphate, octaprenyl-diphosphate and oxaloacetate, but all could be also available in the insect cell.

Other seeds that do not seem to be produced by the co-endocytobiont were not reported before as potentially provided by the host. We assume that these seeds are produced by the insect or present in its diet. Knowledge of the metabolic network of the sharshooter should confirm or disprove the production of these metabolites by the insect host.

### Precursor sets of metabolites involved in the mutualistic association

**Figure 7:**
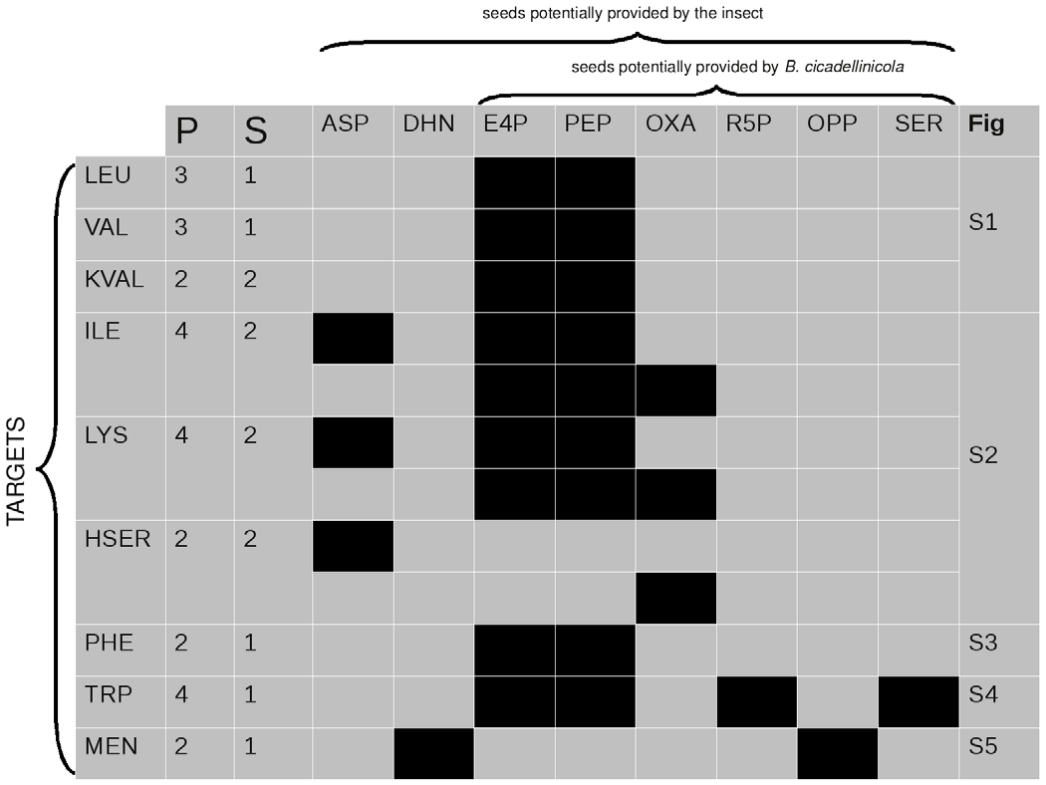
Precursor sets of important metabolites in the metabolic network of *S. muelleri*. Rows correspond to target metabolites and columns to seeds. Column P indicates the total number of precursors and column S the total number of solutions for the corresponding target. A black square means that a seed is present in a solution. The last column indicates the number of the figure in the supplementary material that displays the metabolic sub-network linking the precursors sets and the targets. **LEU**: L-leucine; **ILE**: L-isoleucine; **HSER**: homoserine; **KVAL**: 2-ketovaline; **LYS**:L-lysine; **PHE**:L-phenylalanine; **TRP**:L-tryptophane; **VAL**:L-valine; **MEN**:menaquinone; **ASP**:L-aspartate; **E4P**:erythrose-4-phosphate; **PEP**:phosphoenolpyruvate; **OXA**:oxaloacetate; **R5P**:ribose-5-phosphate; **OPP**:octaprenyl-diphosphate; **SER**: serine; **DHN**:1,4-dihydroxy-2-naphthoate.

**Figure 8:**
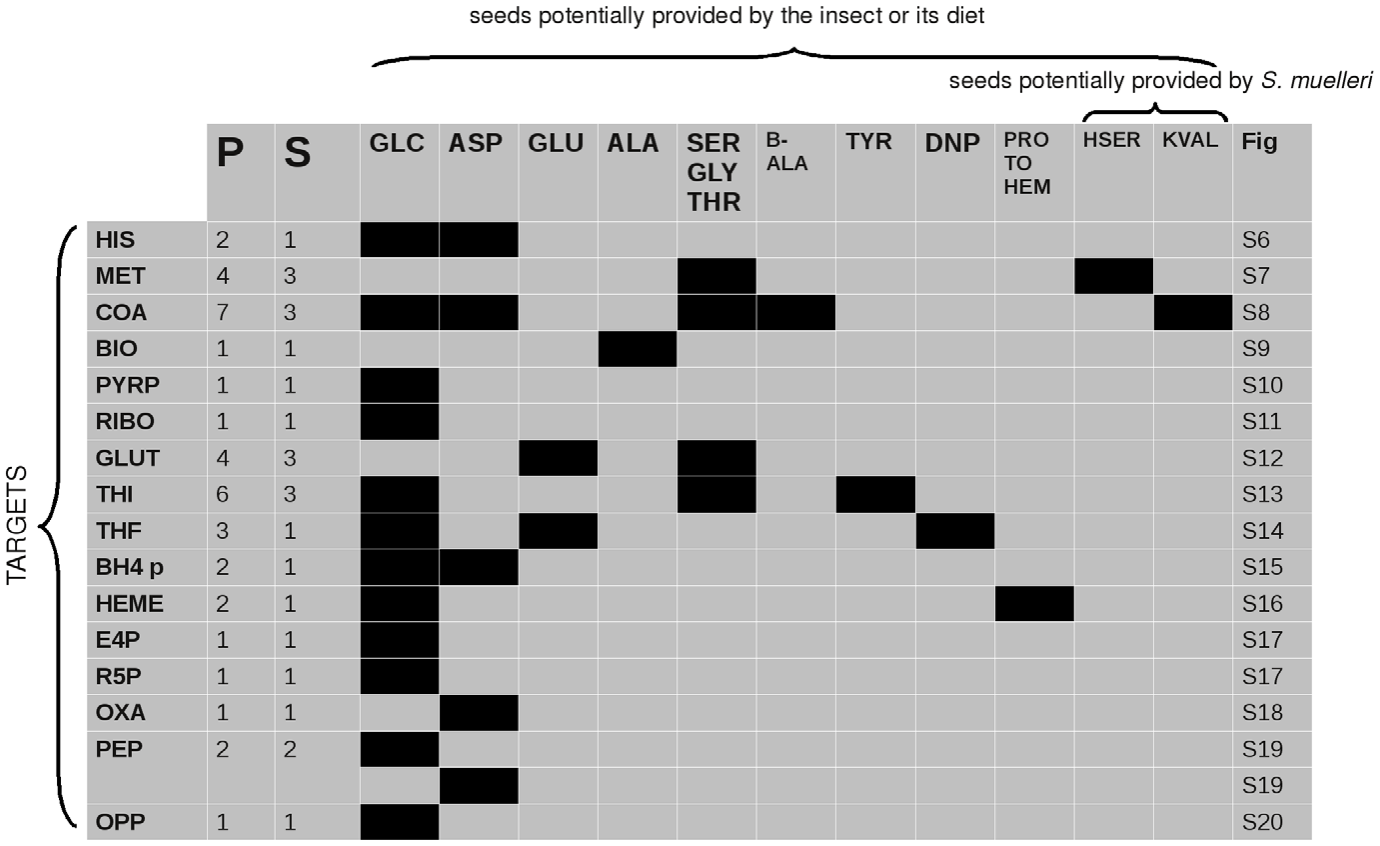
Precursor sets of important metabolites in the metabolic network of *B. cicadellinicola*. Rows correspond to target metabolites and columns to seeds. Column P indicates the total number of precursors and column S the total number of solutions for the corresponding target. A black square means that a seed is present in a solution. The last column indicates the number of the figure in the supplementary material that displays the metabolic sub-network linking the precursors sets and the targets. **HIS**:L-histidine; **MET**:L-methionine; **COA**:coenzyme A; **BIO**:biotin; **PYRP**:pyridoxal-5′-phosphate; **RIBO**:riboflavin; **GLUT**:glutathione; **THI**:thiamine; **THF**:tetrahydrofolate; **BH4 p**:BH4 precursor; **E4P**:erythrose-4-phosphate; **R5P**:ribose-5-phosphate; **OXA**:oxaloaceatate; **PEP**:phosphoenolpyruvate; **OPP**:octaprenyl-diphosphate; **GLC**:glucose; **ASP**:L-aspartate; **ALA**:L-alpha-alanine; **SER**:serine; **GLY**:glycine; **THR**:threonine; **HSER**:homoserine; **KVAL**:2-ketovaline; **B-ALA**:Beta-alanine; **DNP**:dihydroneopterin.

Figures 7 and 8 indicate the precursor sets for the target metabolites selected in the metabolic networks of the two endocytobionts (see Methods).

The parsimony of the metabolic network of both bacteria is reflected in the small number of precursor sets found for the target metabolites to which we applied PITUFO: the maximum number of solutions for a target is only three and the maximum total number of involved precursors is seven.

For *S. muelleri*, apart from menaquinone, all targets are amino acids, which explains the uniformity of the results. Two seeds are present in all minimal precursor sets computed for these amino acids (except homoserine): erythrose-4-phosphate and phosphoenolpyruvate. Both are potentially provided by *B. cicadellinicola*. We found oxaloacetate and aspartate as alternative precursors for the synthesis of isoleucine and lysine. Indeed, each one can produce the other by the same reversible reaction in the metabolic network of *S. muelleri* (Figure 6). This leads to two possible scenarii, depending on which one of them is actually provided. Aspartate is one of the primary components of the xylem sap, it is thus reasonable to think that this compound should be provided by the host. In the metabolic network of *S. muelleri*, aspartate is involved in other reactions that in particular participate in the synthesis of other amino acids. Oxaloacetate is only involved in the reaction that produces aspartate. Since *S. muelleri* is able to produce aspartate from oxaloacetate, and since the former is not used in other reactions, the import of oxaloacetate by *S. muelleri* seems to be more realistic than the import of aspartate. Moreover, *B. cicadellinicola* could provide oxaloacetate while the bacterium is able to synthesise it from aspartate (see Figure 8).

Some seeds appear as obligatory in the synthesis of several targets in *B. cicadellinicola*. Glucose and aspartate, reported as provided by the insect cell, thus appear as obligatory for the synthesis of, respectively, twelve and five target compounds.

As mentioned previously, serine, glycine and threonine have been detected as alternative seeds by the Borenstein method [10]. For *B. cicadellinicola*, they appear in the minimal precursor sets for methionine, coenzyme A, glutathione and thiamine. McCutcheon *et al.* suggested that homoserine and 2-ketovaline, potentially provided by *S. muelleri*, could be precursors of metabolites supplied by *B. cicadellinicola*. Homoserine was reported as a precursor of methionine and 2-ketovaline as a precursor of coenzyme A [7]. Our results confirm these hypotheses. For methionine, our method adds precision by indicating also the alternative precursors serine-glycine-threonine. For coenzyme A, our method further suggests this triplet and also 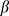-alanine as obligatory precursors.

Interestingly, we observed that only methionine and coenzyme-A require metabolites provided by *S. muelleri*. Moreover, the metabolites needed by the other targets could be all potentially acquired from the host cell by *B. cicadellinicola*.

## Discussion

Graph-based modelling of the metabolic networks of *B. cicadellinicola* and *S. muelleri* enabled us to complete and precise the description of the metabolic exchanges between these two endocytobionts and with their host, the sharpshooter *Homalodisca coagulata*. By automatically computing the set of seeds for each metabolic network, we thus offer the first exhaustive list of metabolites potentially imported by *S. muelleri* and *B. cicadellinicola*. By using our method to find precursor sets for given target compounds, we provide a general and detailed view of the metabolic exchanges that potentially lead to the synthesis of metabolites involved in the mutualistic association.

The definition of seeds by Borenstein *et al.* [10] allowed to indicate alternative ones that could not be found only by defining the seeds as metabolites not produced by any reaction. We found only two instances of such alternative seeds: oxaloacetate-aspartate in the metabolic network of *S. muelleri* and glycine-serine-threonine in the metabolic network of *B. cicadellinicola*. The method of Borenstein *et al.* remains highly suitable to detect seeds in metabolic networks where many reactions cannot be assigned a direction.

From the list of seeds previously defined, the method we developed, PITUFO, was able to find the precursor sets of metabolites reported as involved in the symbiotic association. Contrary to previous methods, PITUFO is an exact algorithm and returns all the precursor sets for a given target compound. In addition, by explicitly taking into account cycles in the definition of precursors and in the algorithm, PITUFO is able to find solutions not reachable by the previous methods. Unfortunately, because the implementation of previous methods is not available or is dataset-dependent, we were not able to compare their performance with the one of PITUFO.

Most of our results could only hardly be found by manual analysis of the metabolic pathways. However, the pertinence of our or of previous analyses is highly linked to the quality of the metabolic network reconstructions. The most time-consuming part in this study was then to refine the metabolic reconstructions available for the two bacteria (see Methods). Interestingly, the methods we used to find seeds and precursor sets also helped us to refine the metabolic reconstructions when some inconsistencies were found. There are several ways to complete this study and to improve the tools that we used. First, PITUFO only returns sets of precursors and not the possible hyperpaths between them and the target compounds. The identification of key metabolites, such as those involved in hyperpath intersections, and compression of the information contained in the metabolic hyperpaths could be a way to provide results easier to interpret for the analyst. When alternative precursor sets are indicated, it would be interesting to point to those that are the most likely to be actually used. Taking into account the stoichiometric coefficients would allow to prune precursor sets not consistent with the stoichiometric constraints. Measuring the production rate of the target compounds would be a way to sort the precursor sets. Finally, PITUFO is restricted to the identification of all the minimal precursor sets leading to the production of the set of targets specified by the user, putting aside the other metabolic functions, even vital for the organism. The identification of all minimal precursor sets leading to the production of both essential metabolites (e.g. those participating to the biomass) and metabolites involved in the mutualistic association was beyond the scope of this study but is certainly of interest and will be developed in the future.

For both bacteria, the number of seeds that we identified is very reduced, even considering that our study is limited to the carbon metabolism of small molecules. This means that the global reduction of the metabolism in the symbionts comes with a reduction in the number of metabolites imported from the host cell. The identification by PITUFO of a unique precursor set for most of the selected target compounds shows that there are almost no alternative sources to produce essential compounds. Indeed, the mutualistic association of the symbionts with their host is very ancient (70 to 100 millions years for *B. cicadellinicola* and approximatively 280 millions years for *S. muelleri*) [21]. The stability of their environment, particularly because of their vertical mode of transmission, made their metabolism specialised in the exploitation of a restricted set of substrates.

However, our results showed that the two bacteria use very differently their environment. Indeed, only three seeds common to the two metabolic networks have been identified. Even the common seeds have a completely different fate in the two bacteria as they are not involved in the same metabolic pathways. The complementarity of the two metabolisms is then not only manifested in the metabolic capabilities of each organism but also by their different use of the nutrients available in the host cell.

The set of seeds identified in the metabolic network of *B. cicadellinicola* is mainly composed of amino acids or related metabolites such as 2-ketovaline or homoserine. Three seeds identified in the metabolic network of *S. muelleri* are also amino acids. The presence of amino acids in the seeds identified in *B. cicadellinicola* (glutamate, lysine, alanine, serine, aspartate and glycine) or in *S. muelleri* (serine, aspartate and cysteine) is interesting in the sense that they are not only provided as essential building blocks of proteins but also as starting points of the biosynthesis of other metabolites. This clarifies the role of the exchanged amino acids as reported in earlier studies [6], [7]. Conversely, the absence of other amino acids in the seeds indicates that they do not participate to the formation of the carbon backbone of other compounds.

Some seeds automatically defined in our study were already mentioned in earlier studies [7]. Aspartate, identified as a common seed in both metabolic networks, is an amino acid indicated to exist in great concentration in the xylem sap that the sharpshooter feeds upon [7]. Its large availability in the direct environment of the two bacteria makes of it an efficient source for the production of other metabolites. Furthermore, a specific aspartate transporter has been annotated in the *B. cicadellinicola* genome. Other metabolites such as non-essential amino acids and glucose, the only sugar identified as seed in the metabolic network of *B. cicadellinicola*, were also mentioned as components of the xylem sap and then available for the two symbionts [7].

However, some metabolites mentioned as highly present in the xylem sap were not identified in our set of seeds. For instance, arginine, an amino acid which is abundant in proteins, is absent from the metabolic network of *B. cicadellinicola* and appears only as output in the metabolic network of *S. muelleri*. Glutamine in both metabolic networks is only used as a nitrogen source and thus does not appear in the filtered metabolic networks. Malate is completely absent from the metabolic network of *S. muelleri*. It is produced from fumarate in the metabolic network of *B. cicadellinicola* and then does not need to be imported.

Three seeds identified in the metabolic network of *S. muelleri* are glycolytic products. Erythrose-4-phosphate and ribose-5-phosphate are commonly produced in the pentose phosphate pathway. Phosphoenolpyruvate is commonly produced during glycolysis. PITUFO returned erythrose-4-phosphate and phosphoenolpyruvate as obligatory precursors in the synthesis of the metabolites that *S. muelleri* provides to the symbiotic system, except homoserine and menaquinone. Ribose-5-phosphate was identified as obligatory precursor for tryptophan. These three metabolites, as well as oxaloacetate and octaprenyl diphosphate, are produced by the metabolic network of *B. cicadellinicola*. However, it is likely that these metabolites could be made available by the insect host. This means that the carbon metabolism of *S. muelleri* may be completely independent of the metabolic network of *B. cicadellinicola*. On the contrary, the two essential amino acids (threonine and lysine) identified as seeds for *B. cicadellinicola* are certainly not produced by the insect host nor present in its diet and must be provided by *S. muelleri*. The carbon metabolism of *B. cicadellinicola* therefore appears as dependent on the metabolism of *S. muelleri*, at least for these two amino acids. This dependence is added to the obligatory supply of other essential amino acids by *S. muelleri* that are required for protein biosynthesis in *B. cicadellinicola* [6], [7].

However, among the precursors identified for the synthesis of metabolites that *B. cicadellinicola* passes on to the symbiotic system, only methionine and coenzyme A need metabolites produced by *S. muelleri*: homoserine and 2-ketovaline. The first one may be produced by the plant and then be present in the diet of the insect, and the second one may be produced by the insect via the degradation of valine. This suggests that *B. cicadellinicola*, as well as *S. muelleri*, may be only dependent on the metabolites obtained from the insect to produce the metabolites provided to the symbiotic system. Indeed, threonine and lysine which are supplied by *S. muelleri* to *B. cicadellinicola*, are only exploited by the latter to produce its proteins.

The reconstruction of the metabolic network of the host and a better knowledge about the metabolome of the plants that the insect feeds upon will inform us whether some seeds, such as intermediates in the biosynthesis of essential amino acids, are actually produced by the insect or present in its diet.

One challenging issue concerns how the metabolites are exchanged between the three partners. The annotation of transporters for amino acids and sugars in *B. cicadellinicola* [6] gives only a partial answer to this question. Indeed, very few transporters were found during the annotation of the genome of *S. muelleri*, and none corresponds to the seeds that we identified [7]. This means that other scenarii have to be proposed to explain the exchanges of metabolites in the symbiotic system. In particular, the cells of *B. cicadellinicola* often appear to adhere to the surface of the much larger cells of *S. muelleri* [6]. This proximity should facilitate the exchanges between the two bacteria. However, the two bacteria seem to be not always in the same cells [6]. This poses the problem of how the essential amino acids are provided to *B. cicadellinicola*.

One other remaining interesting question is evolutionary: how did the reductions of the metabolism of the two symbionts get organised during evolution to reach their current complementarity? A recent study compared the metabolic gene sets of two pairs of co-resident endocytobionts. One pair was formed by the two endocytobionts of the sharpshooter studied in this paper and the other pair was formed by another strain of *S. muelleri* (SMDSEM) and by *Hodgkinia cicadicola*, found in the cells of cicadas [22]. The authors showed that the two strains of *S. muelleri* exhibit almost identical metabolic capabilities. They suggested also that, although phylogenetically distant, *H. cicadicola* and *B. cicadellinicola* have converged on similar metabolic functions, especially those that are complementary to the metabolism preserved in *S. muelleri*.

The application of such methods as we used in this study to this other pair of co-resident endosymbionts should allow to identify some common patterns in the sharing of a set of nutrients and their use in the metabolic networks of the different partners. The extension to other endosymbiotic systems could provide us with crucial information to understand the establishment of such nutritional associations.

## Supporting Information

Dataset S1 Metabolic network of *S. muelleri* in SBML format.
(0.08 MB XML)

Dataset S2 Metabolic network of *B. cicadellinicola* in SBML format.
(0.27 MB XML)

Dataset S3 Filtered metabolic network of *S. muelleri* in SBML format.
(0.06 MB XML)

Dataset S4 Filtered metabolic network of *B. cicadellinicola* in SBML format.
(0.25 MB XML)

Figure S1 Sub-network corresponding to the production of leucine and valine from erythrose-4-phosphate and phosphoenolpyruvate in *Sulcia muelleri*. Squares correspond to reactions and circles to metabolites. The colour of the edges differentiates the two sides of a reaction.
(0.15 MB PDF)

Figure S2 Sub-network corresponding to the production of lysine, homoserine and isoleucine from erythrose-4-phosphate, oxaloacetate, aspartate and phosphoenolpyruvate in *Sulcia muelleri*. Squares correspond to reactions and circles to metabolites. The colour of the edges differentiates the two sides of a reaction.
(0.16 MB PDF)

Figure S3 Sub-network corresponding to the production of phenylalanine from erythrose-4-phosphate and phosphoenolpyruvate in *Sulcia muelleri*. Squares correspond to reactions and circles to metabolites. The colour of the edges differentiates the two sides of a reaction.
(0.11 MB PDF)

Figure S4 Sub-network corresponding to the production of tryptophane from erythrose-4-phosphate, ribose-5-phosphate, serine and phosphoenolpyruvate in *Sulcia muelleri*. Squares correspond to reactions and circles to metabolites. The colour of the edges differentiates the two sides of a reaction.
(0.12 MB PDF)

Figure S5 Sub-network corresponding to the production of menaquinone from octaprenyl-diphosphate and 1,4-dihydroxy-2-naphthoate in *Sulcia muelleri*. Squares correspond to reactions and circles to metabolites. The colour of the edges differentiates the two sides of a reaction.
(0.06 MB PDF)

Figure S6 Sub-network corresponding to the production of histidine from glucose, and aspartate in *B. cicadellinicola*. Squares correspond to reactions and circles to metabolites. The colour of the edges differentiates the two sides of a reaction.
(0.13 MB PDF)

Figure S7 Sub-network corresponding to the production of methionine from serine, glycine, threonine and homoserine in *B. cicadellinicola*. Squares correspond to reactions and circles to metabolites. The colour of the edges differentiates the two sides of a reaction.
(0.09 MB PDF)

Figure S8 Sub-network corresponding to the production of co-enzyme A from β-alanine, glucose, aspartate, glycine, serine, threonine and keto-isovalerate in *B. cicadellinicola*. Squares correspond to reactions and circles to metabolites. The colour of the edges differentiates the two sides of a reaction.
(0.13 MB PDF)

Figure S9 Sub-network corresponding to the production of biotin from alanine in *B. cicadellinicola*. Squares correspond to reactions and circles to metabolites. The colour of the edges differentiates the two sides of a reaction.
(0.08 MB PDF)

Figure S10 Sub-network corresponding to the production of pyridoxal-5′-phosphate from glucose in *B. cicadellinicola*. Squares correspond to reactions and circles to metabolites. The colour of the edges differentiates the two sides of a reaction.
(0.14 MB PDF)

Figure S11 Sub-network corresponding to the production of riboflavin from glucose in *B. cicadellinicola*. Squares correspond to reactions and circles to metabolites. The colour of the edges differentiates the two sides of a reaction.
(0.10 MB PDF)

Figure S12 Sub-network corresponding to the production of glutathione from glutamate, glycine, serine and threonine in *B. cicadellinicola*. Squares correspond to reactions and circles to metabolites. The colour of the edges differentiates the two sides of a reaction.
(0.12 MB PDF)

Figure S13 Sub-network corresponding to the production of thiamine from glucose, serine, glycine, tyrosine and threonine in *B. cicadellinicola*. Squares correspond to reactions and circles to metabolites. The colour of the edges differentiates the two sides of a reaction.
(0.19 MB PDF)

Figure S14 Sub-network corresponding to the production of tetrahydrofolate from dihydroneopterin, glucose and glutamate in *B. cicadellinicola*. Squares correspond to reactions and circles to metabolites. The colour of the edges differentiates the two sides of a reaction.
(0.15 MB PDF)

Figure S15 Sub-network corresponding to the production of BH4 precursor from glucose and aspartate in *B. cicadellinicola*. Squares correspond to reactions and circles to metabolites. The colour of the edges differentiates the two sides of a reaction.
(0.16 MB PDF)

Figure S16 Sub-network corresponding to the production of heme from glucose and protoheme in *B. cicadellinicola*. Squares correspond to reactions and circles to metabolites. The colour of the edges differentiates the two sides of a reaction.
(0.13 MB PDF)

Figure S17 Sub-network corresponding to the production of erythrose-4-phosphate and of ribose-5-phosphate from glucose in *B. cicadellinicola*. Squares correspond to reactions and circles to metabolites. The colour of the edges differentiates the two sides of a reaction.
(0.11 MB PDF)

Figure S18 Sub-network corresponding to the production of oxaloacetate from aspartate in *B. cicadellinicola*. Squares correspond to reactions and circles to metabolites. The colour of the edges differentiates the two sides of a reaction.
(0.06 MB PDF)

Figure S19 Sub-network corresponding to the production of phosphoenolpyruvate from glucose or aspartate in *B. cicadellinicola*. Squares correspond to reactions and circles to metabolites. The colour of the edges differentiates the two sides of a reaction.
(0.10 MB PDF)

Figure S20 Sub-network corresponding to the production of octaprenyl-diphosphate from glucose in *B. cicadellinicola*. Squares correspond to reactions and circles to metabolites. The colour of the edges differentiates the two sides of a reaction.
(0.15 MB PDF)

Table S1 Reactions manually removed from the automatic recontruction of the small-molecule metabolism of *S. muelleri*. IR: isolated reaction; ES: enzyme specificity misannotation; MM: reaction involving macromolecules out of the scope of the study.
(0.04 MB PDF)

Table S2 Reactions manually removed from the automatic recontruction of the small-molecule metabolism of *B. cicadellinicola*. GR: generic reaction; IR: isolated reactions; ES: enzyme specificity misannotations; MM: reactions involving macromolecules out of the scope of the study; NC: reactions not corresponding to carbon atom transferts.
(0.05 MB PDF)

Table S3 List of reactions in the metabolic network we build for *S. muelleri* and the evidences used to assign the direction of the reactions. The metabolites kept in the reactions after filtering appear in bold. M: MetaCyc pathways evidence; T: topological evidence.
(0.06 MB PDF)

Table S4 List of reactions in the metabolic network we build for *B. cicadellinicola* and the evidences used to assign the direction of the reactions. The metabolites kept in the reactions after filtering appear in bold. M: MetaCyc pathways evidence; T: topological evidence.
(0.11 MB PDF)

Table S5 List of transformations cofactors used to automatically filter the metabolic networks of *B. cicadellinicola* and of *S. muelleri*. Each line corresponds to a transformation of cofactors. If the metabolites written in bold in the first column appear in a side of a reaction and the metabolites written in bold in the second column appear in the other side, they and the corresponding subproducts (written in normal font) are removed in the reaction. The third column and the fourth column indicate the number of affected reactions in the metabolic networks of *S. muelleri* and *B. cicadellinicola*.
(0.05 MB PDF)

Text S1 Sub-networks linking each target metabolite to their precursor sets.
(2.11 MB PDF)
