# Supplementary material for: Graph-Based Analysis of the Metabolic Exchanges between Two Co-Resident Intracellular Symbionts, Baumannia cicadellinicola and Sulcia muelleri, with Their Insect Host, Homalodisca coagulata
Source: PLoS Comput Biol. 2010 Sep 2;6(9):e1000904. doi: 10.1371/journal.pcbi.1000904 (PMC2936742; doi:10.1371/journal.pcbi.1000904)

Figure S5. Sub-network corresponding to the production of menaquinone from octaprenyl-diphosphate and 1,4-dihydroxy-2-naphthoate in *Sulcia muelleri*. Squares correspond to reactions and circles to metabolites. The colour of the edges differentiates the two sides of a reaction.

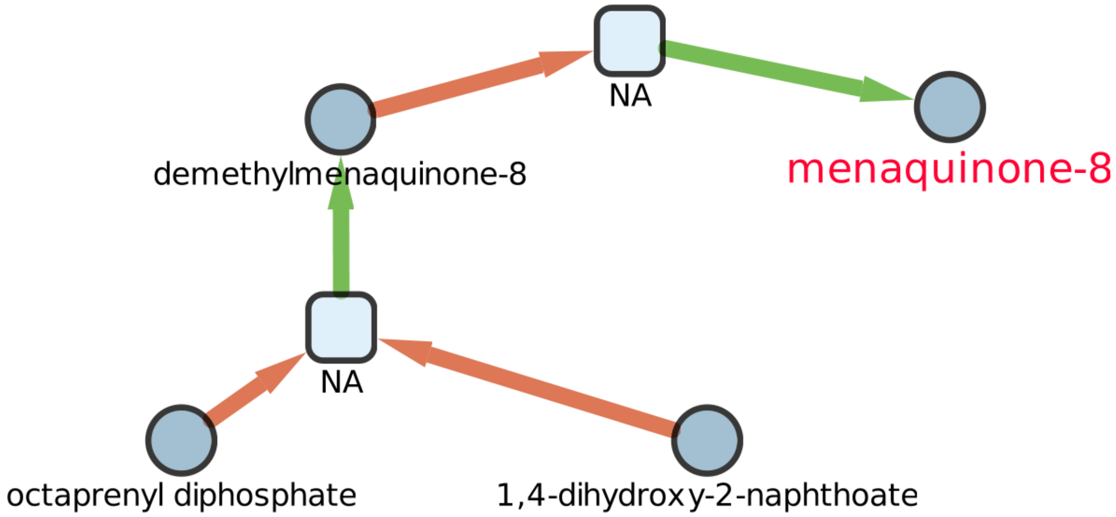

Supplement: Figure S5 — Sub-network corresponding to the production of menaquinone from octaprenyl-diphosphate and 1,4-dihydroxy-2-naphthoate in Sulcia muelleri. Squares correspond to reactions and circles to metabolites. The colour of the edges differentiates the two sides of a reaction. (0.06 MB PDF) [file pcbi.1000904.s009.pdf]
