# Supplementary material for: Graph-Based Analysis of the Metabolic Exchanges between Two Co-Resident Intracellular Symbionts, Baumannia cicadellinicola and Sulcia muelleri, with Their Insect Host, Homalodisca coagulata
Source: PLoS Comput Biol. 2010 Sep 2;6(9):e1000904. doi: 10.1371/journal.pcbi.1000904 (PMC2936742; doi:10.1371/journal.pcbi.1000904)

Figure S6. Sub-network corresponding to the production of histidine from glucose, and aspartate in *B. cicadellinicola*. Squares correspond to reactions and circles to metabolites. The colour of the edges differentiates the two sides of a reaction.

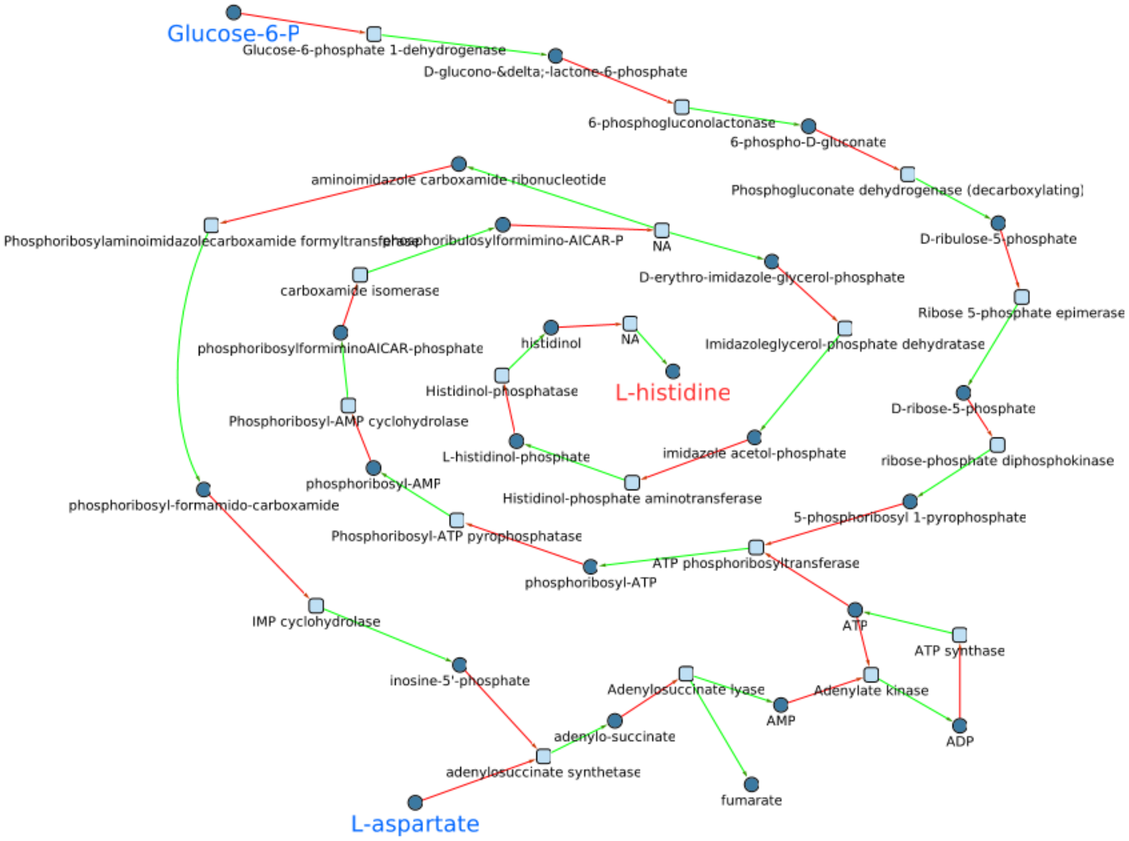

Supplement: Figure S6 — Sub-network corresponding to the production of histidine from glucose, and aspartate in B. cicadellinicola. Squares correspond to reactions and circles to metabolites. The colour of the edges differentiates the two sides of a reaction. (0.13 MB PDF) [file pcbi.1000904.s010.pdf]
