# Supplementary material for: Graph-Based Analysis of the Metabolic Exchanges between Two Co-Resident Intracellular Symbionts, Baumannia cicadellinicola and Sulcia muelleri, with Their Insect Host, Homalodisca coagulata
Source: PLoS Comput Biol. 2010 Sep 2;6(9):e1000904. doi: 10.1371/journal.pcbi.1000904 (PMC2936742; doi:10.1371/journal.pcbi.1000904)

Figure S13. Sub-network corresponding to the production of thiamine from glucose, serine, glycine, tyrosine and threonine in *B. cicadellinicola*. Squares correspond to reactions and circles to metabolites. The colour of the edges differentiates the two sides of a reaction.

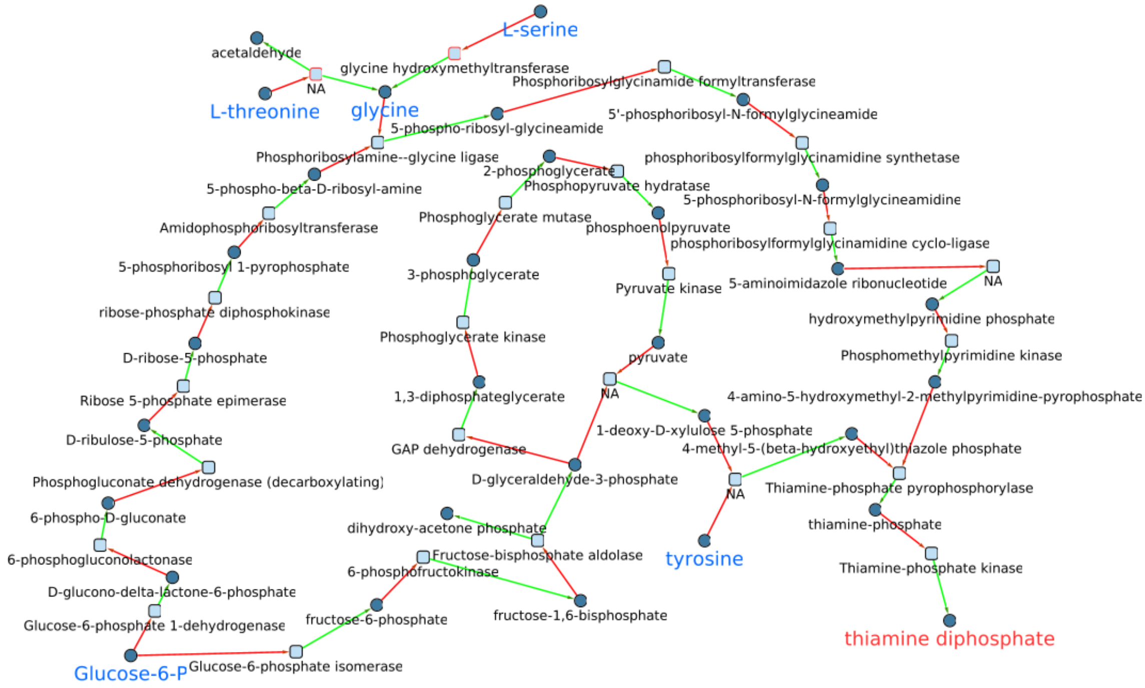

Supplement: Figure S13 — Sub-network corresponding to the production of thiamine from glucose, serine, glycine, tyrosine and threonine in B. cicadellinicola. Squares correspond to reactions and circles to metabolites. The colour of the edges differentiates the two sides of a reaction. (0.19 MB PDF) [file pcbi.1000904.s017.pdf]
