# Supplementary material for: Graph-Based Analysis of the Metabolic Exchanges between Two Co-Resident Intracellular Symbionts, Baumannia cicadellinicola and Sulcia muelleri, with Their Insect Host, Homalodisca coagulata
Source: PLoS Comput Biol. 2010 Sep 2;6(9):e1000904. doi: 10.1371/journal.pcbi.1000904 (PMC2936742; doi:10.1371/journal.pcbi.1000904)

Figure S17. Sub-network corresponding to the production of erythrose-4-phosphate and of ribose-5-phosphate from glucose in *B. cicadellinicola.* Squares correspond to reactions and circles to metabolites. The colour of the edges differentiates the two sides of a reaction.

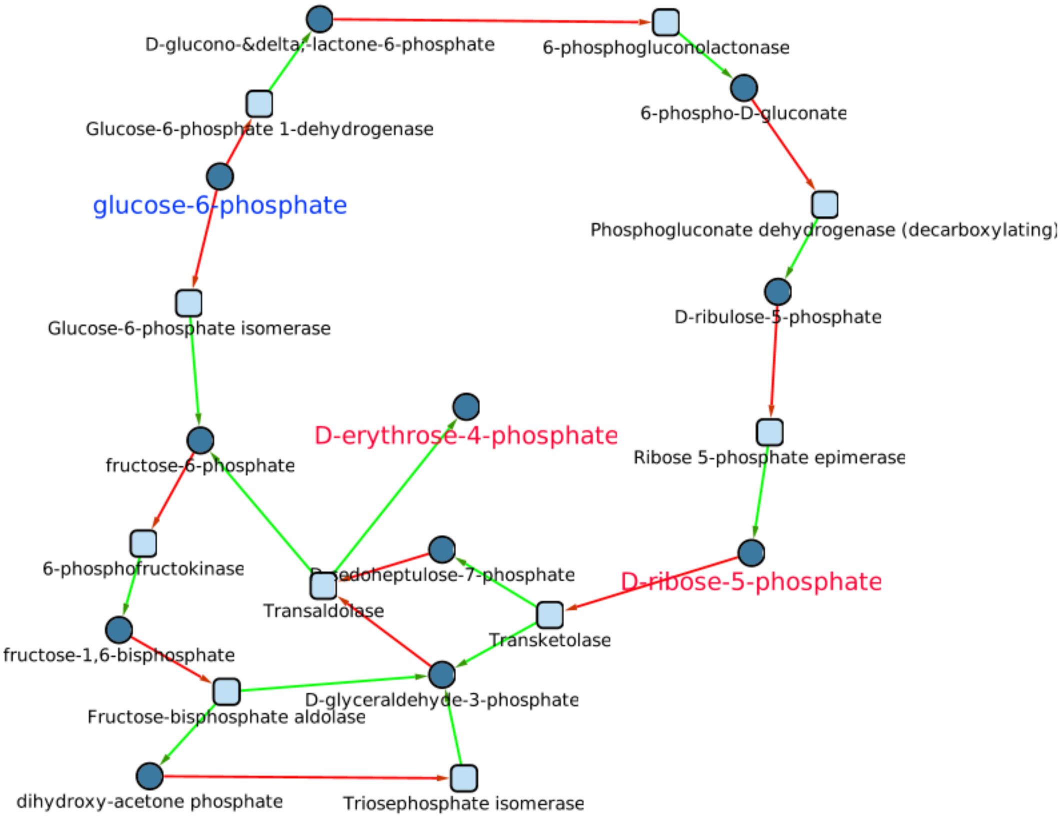

Supplement: Figure S17 — Sub-network corresponding to the production of erythrose-4-phosphate and of ribose-5-phosphate from glucose in B. cicadellinicola. Squares correspond to reactions and circles to metabolites. The colour of the edges differentiates the two sides of a reaction. (0.11 MB PDF) [file pcbi.1000904.s021.pdf]
