# Supplementary material for: Graph-Based Analysis of the Metabolic Exchanges between Two Co-Resident Intracellular Symbionts, Baumannia cicadellinicola and Sulcia muelleri, with Their Insect Host, Homalodisca coagulata
Source: PLoS Comput Biol. 2010 Sep 2;6(9):e1000904. doi: 10.1371/journal.pcbi.1000904 (PMC2936742; doi:10.1371/journal.pcbi.1000904)
Supplement: Table S1 — Reactions manually removed from the automatic recontruction of the small-molecule metabolism of S. muelleri. IR: isolated reaction; ES: enzyme specificity misannotation; MM: reaction involving macromolecules out of the scope of the study. (0.04 MB PDF) [file pcbi.1000904.s025.pdf]

Table S1. Reactions manually removed from the automatic recontruction of the small-molecule metabolism of *S. muelleri*. **IR**: isolated reaction; **ES**: enzyme specificity misannotation; **MM**: reaction involving macromolecules out of the scope of the study.

Reaction BioCyc ID	Name	Formula	Evidence to remove it
2-OCTAPRENYL-METHOXY-BENZOQ-METH-RXN	NA	S-adenosyl-L-methionine + 2-octaprenyl-6-methoxy-1,4-benzoquinol → H+ + S-adenosyl-L-homocysteine + 2-octaprenyl-3-methyl-6-methoxy-1,4-benzoquinol	IR, ES
HYDROXYPYRROLINEDEH-RXN	1-pyrroline-5-carboxylate dehydrogenase	H2O + NAD+ + pyrroline-hydroxy-carboxylate → H+ + L-erythro-4-hydroxy-glutamate + NADH	IR, ES
RXN0-2625	NA	a mismatched DNA base pair ↔ a properly matched DNA base	IR, MM
3-SULFINOALANINE-AMINOTRANSFERASE-RXN	NA	2-oxoglutarate + 3-sulfinoalanine → L-glutamate + 3-sulfinyl-pyruvate	IR, ES
DIHYDLIPOXN-RXN	lipoamide dehydrogenase	dihydrolipoamide + NAD+ ↔ lipoamide + NADH + H+	IR, ES
2.8.1.8-RXN	lipoyl synthase	a protein 6-N-(octanoyl)lysine + 2 S0 + 2 S-adenosyl-L-methionine ↔ a protein 6-N-(lipoyl)lysine + 2 L-methionine +2 5'-deoxyadenosine	IR, MM
TRNA-PSEUDOURIDINE-SYNTHASE-I-RXN	tRNA-pseudouridine synthase I	tRNA uridine ↔ H+ + tRNA pseudouridine	IR, MM
3.5.1.88-RXN	Peptide deformylase	H2O + formyl-L-methionyl peptide ↔ formate + H+ + methionyl peptide	IR, MM
OPPSYN-RXN	NA	isopentenyl diphosphate + a trans-polyisoprenyln-PP ↔ diphosphate + a trans-polyisoprenyln-PP	IR
SUCCGLU-SEMIALDEHYDE-SUCCGLU-RXN	NA	N2-succinyl-L-glutamate 5-semialdehyde + NAD+ ↔ N2-succinylglutamate + NADH	IR
2OXOGLUTARATEDEH-RXN	NA	coenzyme A + NAD+ + 2-oxoglutarate → NADH + succinyl-CoA + CO2	IR, ES
RXN-10	NA	ATP + L-aspartate + O-ureidohomoserine → H+ + diphosphate + canavaninosuccinate + adenosine-5'-phosphate	IR, ES
RXN-22	NA	Canavaninosuccinate → fumarate + L-canavanine	IR, ES
RXN-9	NA	carbamoyl-phosphate + L-canaline → phosphate + O-ureidohomoserine	IR, ES
SELENOCYSTEINE-LYASE-RXN	Selenocysteine lyase	a reduced electron acceptor + L-selenocysteine ↔ selenide + L-alanine + an oxidized electron acceptor	MM

Reaction BioCyc ID	Name	Formula	Evidence to remove it
RXN0-2023	NA	L-cysteine + ATP + uridine ↔ diphosphate + L-alanine + 2-thiouridine + adenosine-5'-phosphate	MM
