# Supplementary material for: Graph-Based Analysis of the Metabolic Exchanges between Two Co-Resident Intracellular Symbionts, Baumannia cicadellinicola and Sulcia muelleri, with Their Insect Host, Homalodisca coagulata
Source: PLoS Comput Biol. 2010 Sep 2;6(9):e1000904. doi: 10.1371/journal.pcbi.1000904 (PMC2936742; doi:10.1371/journal.pcbi.1000904)
Supplement: Table S2 — Reactions manually removed from the automatic recontruction of the small-molecule metabolism of B. cicadellinicola. GR: generic reaction; IR: isolated reactions; ES: enzyme specificity misannotations; MM: reactions involving macromolecules out of the scope of the study; NC: reactions not corresponding to carbon atom transferts. (0.05 MB PDF) [file pcbi.1000904.s026.pdf]

Table S2. Reactions manually removed from the automatic recontruction of the small-molecule metabolism of *B. cicadellinicola*. **GR:** generic reaction; **IR**: isolated reactions; **ES**: enzyme specificity misannotations; **MM**: reactions involving macromolecules out of the scope of the study; **NC:** reactions not corresponding to carbon atom transferts

Reaction BioCyc ID	Name	Formula	Evidence to remove it
MALATE-DEHYDROGENASE-ACCEPTOR-RXN	Malate dehydrogenase (acceptor)	malate + an oxidized electron acceptor → a reduced electron acceptor + oxaloacetate	GR
RXN-6401	NA	pantoyl lactone + beta-alanine ↔ pantothenate	ES, IR
2.8.1.8-RXN	Lipoyl synthase	a protein 6-N-(octanoyl)lysine + 2 S0 + 2 S-adenosyl-L-methionine ↔ a protein 6-N-(lipoyl)lysine + 2 L-methionine + 2 D'-deoxyadenosine	MM
QUINOLINATE-SYNTHE-MULTI-RXN	quinolinate synthetase	L-aspartate + dihydroxy-acetone phosphate + O2 ↔ quinolinate + phosphate + H2O2 + 2 H2O	ES
PTAALT-RXN	NA	phosphate + propionyl-CoA -> coenzyme A + propionyl-P	IR
UPPSYN-RXN	NA	isopentenyl diphosphate + a cis,trans-polyisoprenyln-PP ↔ diphosphate + a cis,trans-polyisoprenyln-PP	GR, IR
DECAPCISTRANSFER-RXN	di-trans-poly-cis-decaprenylcistransferase	di-trans,poly-cis-decaprenyl diphosphate + isopentenyl diphosphate ↔ diphosphate + di-trans,poly-cis-undecaprenyl diphosphate	ES, IR
1.17.1.2-RXN	4-hydroxy-3-methylbut-2-enyl diphosphate reductase	Isopentenyl diphosphate + NAD(P)+ + H2O ↔ (E)-4-hydroxy-3-methyl but2-en-1-yl diphosphate + NAD(P)H	ES
6.3.2.10-RXN	UDP-N-acetylmuramoyl alanine-D-glutamyl-lysine--D-alanyl-D-alanine ligase	D-alanyl-D-alanine + UDP-N-acetylmuramoyl-L-alanyl-D-glutamyl-L-lysine + ATP → H+ + ADP + phosphate + UDP-N-acetylmuramoyl-L-alanyl-D-glutamyl-L-lysyl-D-alanyl-D-alanine	ES, IR
RXN-8975	Phospho-N-acetylmuramoyl-pentapeptide-transferase	UDP-N-acetylmuramoyl-L-alanyl-D-glutamyl-L-lysyl-D-alanyl-D-alanine + di-trans,poly-cis-undecaprenyl phosphate → N-acetylmuramoyl-L-alanyl-D-glutamyl-L-lysyl- D-alanyl-D-alanine-diphosphoundecaprenol + uridine-5'-phosphate	ES, IR
NUCLEOSIDE-DIP-KIN-RXN	Nucleoside-diphosphate kinase	ATP + a ribonucleoside diphosphate ↔ ADP + a ribonucleoside triphosphate	GR
RXN0-1	NA	H2O + a 2'-deoxyribonucleoside diphosphate + an oxidized electron acceptor ↔ a reduced electron acceptor + a ribonucleoside diphosphate	GR
RXN-7241	NA	H2O + phytate → phosphate + D-myo-inositol (1,2,3,5,6) pentakisphosphate	IR
6-PHYT-RXN	6-phytase	H2O + phytate → D-myo-inositol (1,2,3,4,5)-pentakisphosphate + phosphate	IR
DIHYDLIPOXN-RXN	NA	Dihydrolipoamide + NAD+ ↔ lipoamide + NADH	IR, ES
2-6-1-57-RXN	aromatic amino acid aminotransferase	an aromatic amino acid + 2-ketoglutarate ↔ an aromatic oxo-acid + L-glutamate	IR, GR

Reaction BioCyc ID	Name	Formula	Evidence to remove it
R343-RXN	Cob(II)yrinic acid a,c-diamide reductase	H+ + FMN + cob(II)yrinate a,c-diamide → FMNH2 + cob(I)yrinate a,c-diamide	IR
2.6.1.7-RXN	Kynurenine--oxoglutarate aminotransferase	2-oxoglutarate + kynurenine ↔ 4-(2-aminophenyl)-2,4-dioxobutanoate + L-glutamate	IR, ES
MYO-INOSITOL-1OR-4-MONOPHOSPHATASE-RXN	Myo-inositol-1(or 4)-monophosphatase	H2O + D-myo-inositol (3)-monophosphate → phosphate + myo-inositol	IR
RXN0-5063	NA	N-6-isopentyl adenosine-37 tRNA + S-adenosyl-L-methionine ↔ S-adenosyl-L-homocysteine + 2-methylthio-N-6-isopentyl adenosine-37 tRNA + 5'-deoxyadenosine + L-methionine	IR
1-7-1-13-RXN	queuine synthase	queuine + 2 NADP+ ↔ 7-cyano-7-carbaguanine	IR
2-HEXADECENAL-REDUCTASE-RXN	2-hexadecenal reductase	2-trans-Hexadecenal + H+ + NADPH → palmitaldehyde + NADP+	IR
3-1-13-1-RXN	Exoribonuclease II	an RNA ↔ a nucleoside-5'-phosphate + an RNA	IR
PSERTRANSAM-RXN	Phosphoserine aminotransferase	L-glutamate + 3-phospho-hydroxypyruvate → 2-oxoglutarate + 3-phospho-serine	IR
RXN-7958	NA	ATP + propionate ↔ ADP + propionyl-P	IR
RXN0-4022	NA	H+ + 7-cyano-7-deazaguanine + NADPH ↔ 7-aminomethyl-7-deazaguanine + NADP+	IR
NADH-DEHYDROGENASE-QUINONE-RXN	NADH dehydrogenase (quinone)	NADH + a quinone ↔ a hydroquinone + NAD+	IR, GR
CARBOXYLESTERASE-RXN	carboxylesterase	H2O + a carboxylic ester ↔ an alcohol + a carboxylate	IR, GR
RXN0-5225		H2O + GlcNAc-1,6-anhMurNAc-L-Ala-gamma-D-Glu-DAP-D-Ala ↔ L-Ala-gamma-D-Glu-DAP-D-Ala + GlcNAc-1,6-anhMurNAc	IR
POLYPHOSPHATE-KINASE-RXN	Polyphosphate kinase	long chain polyphosphate + ATP ↔ long chain polyphosphate + ADP	IR
PEPTIDYLPROLYL-ISOMERASE-RXN	Peptidylprolyl isomerase	peptidylproline (omega = 180) ↔ peptidylproline (omega = 0)	IR
RXN0-5063	NA	N-6-isopentyl adenosine-37 tRNA + S-adenosyl-L-methionine ↔ S-adenosyl-L-homocysteine + 2-methylthio-N-6-isopentyl adenosine-37 tRNA + 5'-deoxyadenosine + L-methionine	IR
RXN-7919	NA	S-(2-hydroxyacyl)glutathione + H2O ↔ glutathione + a 2-hydroxy carboxylate	IR, GR
GLYOXII-RXN	Hydroxy acylglutathione hydrolase	H2O + S-lactoyl-glutathione → H+ + D-lactate + glutathione	IR
DNA-LIGASE-NAD43-RXN	DNA ligase (NAD+)	a deoxynucleotide + NAD+ + (deoxynucleotides)(m) ↔ a deoxynucleotide + nicotinamide mononucleotide + adenosine-5'-phosphate	IR

Reaction BioCyc ID	Name	Formula	Evidence to remove it
4.6.1.10-RXN	6-pyruvoyl tetrahydropterin synthase	2-amino-4-oxo-6-(erythro-1',2',3'-trihydroxyprolyl)-7,8-dihydroxypteridine triphosphate ↔ PPPi + 2-amino-4-oxo-6-(1',2'-dioxoprolyl)-7,8-dihydroxypteridine	IR, ES
ADPSUGPPHOSPHAT-RXN	ADP-sugar diphosphatase	H2O + an ADP-sugar ↔ an alpha-D-aldose-1-phosphate + adenosine-5'-phosphate	IR, GR
SELENOCYSTEINE-LYASE-RXN	Selenocysteine lyase	a reduced electron acceptor + L-selenocysteine ↔ selenide + L-alanine + an oxidized electron acceptor	MM
GMKALT-RXN	T2-induced deoxynucleotide kinase	dGMP + ATP ↔ H+ + 2'-deoxyguanosine-5'-diphosphate + ADP	ES
RXN0-308	NA	a protein L-cysteine + L-cysteine → L-alanine + a protein-S-sulfanylcysteine	MM
TRYPTOPHAN-AMINOTRANSFERASE-RXN	Tryptophan aminotransferase	L-tryptophan + 2-oxoglutarate → L-glutamate + indole-3-pyruvate	IR, ES
2-6-1-58-RXN	Phenylalanine (histidine) aminotransferase	L-phenylalanine + pyruvate → L-alanine + phenylpyruvate	ES
PHEAMINOTRANS-RXN	Aromatic amino acid transferase	L-glutamate + phenylpyruvate ↔ L-phenylalanine + 2-oxoglutarate	ES
2-6-1-28-RXN	Tryptophan--phenylpyruvate aminotransferase	L-tryptophan + phenylpyruvate → L-phenylalanine + indole-3-pyruvate	ES
TYRAMINOTRANS-RXN	Aromatic amino acid transferase	L-glutamate + 4-hydroxyphenylpyruvate ↔ L-tyrosine + 2-oxoglutarate	ES
RXN0-2023	NA	L-cysteine + ATP + uridine ↔ diphosphate + L-alanine + 2-thiouridine + adenosine-5'-phosphate	MM
PSEUDOURIDYLATE-SYNTHASE-RXN	Pseudouridylate synthase	uracil + D-ribose-5-phosphate ↔ H2O + pseudouridine 5'-phosphate	MM
RXN0-1441	ADP-ribose pyrophosphatase	ADP-ribose + H2O ↔ H+ + D-ribose-5-phosphate + adenosine-5'-phosphate	MM
RXN-8631	NA	fructose-1-phosphate ↔ glyceraldehyde + dihydroxyacetone phosphate	ES
GLYCEROL-3-PHOSPHATE-DEHYDROGENASE-RXN	NA	sn-glycerol-3-phosphate + an oxidized electron acceptor ↔ dihydroxy-acetone phosphate + a reduced electron acceptor	GR
SULFITE-REDUCTASE-RXN	Sulfite reductase	H2O + hydrogen sulfide + an oxidized electron acceptor ↔ a reduced electron acceptor + sulfite	GR
HEMN-RXN	NA	coproporphyrinogen III + S-adenosyl-L-methionine → 5'-deoxyadenosine + L-methionine + protoporphyrinogen IX + CO2	IR
3-5-1-88-RXN	Peptide deformylase	H2O + formyl-L-methionyl peptide ↔ formate + H+ + methionyl peptide	MM
OHMETPYRKIN-RXN	Hydroxymethyl pyrimidine kinase	ATP + hydroxymethylpyrimidine → H+ + hydroxymethylpyrimidine phosphate + ADP	IR
RXN-6182	NA	alpha-D-glucose 6-phosphate → D-fructose-6-phosphate	ES

**Reaction BioCyc ID**	**Name**	**Formula**	**Evidence to remove it**
3-6-1-41-RXN	Bis(5'-nucleosyl)-tetraphosphatase (symmetrical)	5',5'''-diadenosine tetraphosphate + H_2O ↔ H^+ + ADP	IR
GLYC3PDEHYDROG-RXN	Glycerol-3-phosphate dehydrogenase	sn-glycerol-3-phosphate + ubiquinone-8 → ubiquinol-8 + dihydroxyacetone phosphate	IR, ES
CYT-UBIQUINOL-OXID-RXN	NA	ubiquinol-8 + oxygen ↔ H_2O + ubiquinone-8	IR, ES
