# Supplementary material for: Graph-Based Analysis of the Metabolic Exchanges between Two Co-Resident Intracellular Symbionts, Baumannia cicadellinicola and Sulcia muelleri, with Their Insect Host, Homalodisca coagulata
Source: PLoS Comput Biol. 2010 Sep 2;6(9):e1000904. doi: 10.1371/journal.pcbi.1000904 (PMC2936742; doi:10.1371/journal.pcbi.1000904)
Supplement: Table S3 — List of reactions in the metabolic network we build for S. muelleri and the evidences used to assign the direction of the reactions. The metabolites kept in the reactions after filtering appear in bold. M: MetaCyc pathways evidence; T: topological evidence. (0.06 MB PDF) [file pcbi.1000904.s027.pdf]

Table S3. List of reactions in the metabolic network we build for *S. muelleri* and the evidences used to assign the direction of the reactions. The metabolites kept in the reactions after filtering appear in bold. **M**: MetaCyc pathways evidence; **T**: topological evidence

**Reaction BioCyc ID**	**Name**	**Formula**	**Evidence to assign the direction**
PHEAMINOTRANS-RXN	Aromatic amino acid transferase	L-glutamate + **phenylpyruvate** → **L-phenylalanine** + 2-ketoglutarate	T
ACETOLACTREDUCTOISOM-RXN	Ketol-acid reducto isomerase	H+ + NADPH + **2-acetolactate** → **2,3-dihydroxy-isovalerate** + NADP+	M
CARBPSYN-RXN	carbamoyl-phosphate synthetase (glutamine-hydrolysing)	L-glutamine + **HCO3-** + ATP + H2O → **carbamoyl-phosphate** + ADP + phosphate + L-glutamate	M
3-ISOPROPYLMALDEHYDROG-RXN	3-isopropylmalate dehydrogenase	NAD+ + **3-isopropylmalate** → **2-isopropyl-3-oxosuccinate** + NADH + H+	M
CHORISMATEMUT-RXN	Chorismate mutase	**Chorismate** → **prephenate**	M
ASPAMINOTRANS-RXN	Aspartate aminotransferase	2-ketoglutarate + **L-aspartate** ↔ **oxaloacetate** + L-glutamate	
ASPARTATEKIN-RXN	Aspartate kinase	ATP + **L-aspartate** → ADP + **L-aspartyl-4-phosphate**	M
ACETYLORNTRANSAM-RXN	Acetylornithine aminotransferase	L-glutamate + **N-acetyl-L-glutamate 5-semialdehyde** → 2-ketoglutarate + **N-acetyl-L-ornithine**	M
ACETYLGLUTKIN-RXN	Acetylglutamate kinase	**N-acetyl-L-glutamate** + ATP → **N-acetylglutamyl-phosphate** + ADP	M
RXN-7562	Acetylornithine aminotransferase	**L-ornithine** + 2-ketoglutarate ↔ L-glutamate + **L-glutamate gamma-semialdehyde**	M
ADOMET-DMK-METHYLTRANSFER-RXN	NA	S-adenosyl-L-methionine + **demethylmenaquinone-8** → **menaquinone-8** + S-adenosyl-L-homocysteine	M
RXN-7800	NA	**2-isopropyl-3-oxosuccinate** → **2-ketoisocaproate** + CO2	M
PRAISOM-RXN	Phosphoribosyl anthranilate isomerase	**N-(5'-phosphoribosyl)-anthranilate** → **1-(o-carboxyphenylamino)-1'-deoxyribulose-5'-phosphate**	M
SUCCINYLDIAMINOPIMTRANS-RXN	Succinyl diaminopimelate aminotransferase	L-glutamate + **N-succinyl-2-amino-6-ketopimelate** → **N-succinyl-L,L-2,6-diaminopimelate** + 2-ketoglutarate	M
ACETOLACTSYN-RXN	Acetolactate synthase	**Pyruvate** → **2-acetolactate** + CO2	M
HOMOSERDEHYDROG-RXN	Homoserine dehydrogenase	H+ + NAD(P)H + **L-aspartate-semialdehyde** → NAD(P)+ + **homoserine**	M
RXN-7183	NA	**pyrroline 5-carboxylate** + NAD(P)+ + H20 → **L-glutamate** + NAD(P)H + H+	M
HOMOSERKIN-RXN	Homoserine kinase	**homoserine** + ATP → ADP + **O-phospho-L-homoserine**	M

Reaction BioCyc ID	Name	Formula	Evidence to assign the direction
ACETOOHBUTREDUCTOISOM-RXN	Ketol-acid reducto isomerase	NADPH + **2-aceto-2-hydroxy-butyrate** → **2,3-dihydroxy-3-methylvalerate** + NADP+	M
TETHYDPICSUCC-RXN	2,3,4,5-tetrahydropyridine-2-carboxylate N-succinyltransferase	H2O + **tetrahydrodipicolinate** + succinyl-CoA → coenzyme A + **N-succinyl-2-amino-6-ketopimelate**	M
N-ACETYLGLUTPREDUCT-RXN	N-acetyl-gamma-glutamyl-phosphate reductase	**N-acetylglutamyl-phosphate** + NADPH → **N-acetyl-L-glutamate 5-semialdehyde** + NADP+ + phosphate	M
2461463469-RXN	NA	**carbamoyl-phosphate** + **N-acetyl-L-ornithine** → **N-acetyl-L-citrulline** + phosphate	M
3-DEHYDROQUINATE-DEHYDRATASE-RXN	3-dehydroquinate dehydratase	**3-dehydroquinate** → **3-dehydro-shikimate** + H2O	M
THRESYN-RXN	Threonine synthase	H2O + **O-phospho-L-homoserine** → **L-threonine** + phosphate	M
RXN0-2381	NA	**indole-3-glycerol-phosphate** → **D-glyceraldehyde-3-phosphate** + **indole**	M
SHIKIMATE-5-DEHYDROGENASE-RXN	Shikimate 5-dehydrogenase	**3-dehydro-shikimate** + NADPH → **shikimate** + NADP+	M
N-ACETYLTRANSFER-RXN	Amino-acid N-acetyltransferase	**L-glutamate** + acetyl-CoA → coenzyme A + **N-acetyl-L-glutamate**	M
2-ISOPROPYLMALATESYN-RXN	2-isopropylmalate synthase	H2O + acetyl-CoA + **2-keto-isovalerate** → coenzyme A + **2-isopropylmalate**	M
CHORISMATE-SYNTHASE-RXN	Chorismate synthase	**5-enolpyruvyl-shikimate-3-phosphate** → phosphate + **chorismate**	M
TRYPSYN-RXN	Tryptophan synthase	**L-serine** + **indole-3-glycerol-phosphate** → **L-tryptophan** + **D-glyceraldehyde-3-phosphate** + H2O	T
RXN-8991	3-isopropylmalate dehydratase	**Isopropylmaleate** + H2O → **3-isopropylmalate**	M
DIAMINOPIMEPIM-RXN	Diamino pimelate epimerase	**L,L-diaminopimelate** → **meso-diaminopimelate**	M
BRANCHED-CHAINAMINOTRANSFERVAL-RXN	Branched-chain amino acid aminotransferase	**L-valine** + 2-ketoglutarate ← L-glutamate + **2-keto-isovalerate**	T
DMK-RXN	NA	**all-trans-octaprenyl diphosphate** + **1,4-dihydroxy-2-naphthoate** → **demethylmenaquinone-8** + diphosphate + CO2	M
DAHPSYN-RXN	2-dehydro-3-deoxy phosphoheptonate aldolase	H2O + **phosphoenolpyruvate** + **D-erythrose-4-phosphate** → **3-deoxy-D-arabino-heptulosonate-7-phosphate** + phosphate	M
3-ISOPROPYLMALISOM-RXN	3-isopropylmalate dehydratase	H2O + **2-isopropylmalate** → **isopropylmaleate** + H2O	M

Reaction BioCyc ID	Name	Formula	Evidence to assign the direction
SPONTPRO-RXN	NA	**L-glutamate gamma-semialdehyde** → **pyrroline 5-carboxylate** + H_2O	T
ORNCARBAMTRANSFER-RXN	Ornithine carbamoyl transferase	**carbamoyl-phosphate** + **L-ornithine** → **citrulline** + phosphate	T
DIHYDROXYISOVALDEHYDRAT-RXN	Dihydroxy-acid dehydratase	**2,3-dihydroxy-isovalerate** → **2-keto-isovalerate** + H_2O	M
PREPHENATEDEHYDRAT-RXN	Prephenate dehydratase	**Prephenate** → **phenylpyruvate** +H_2O + CO_2	M
ARGSUCCINSYN-RXN	Arginino succinate synthetase	**citrulline** + ATP + **L-aspartate** → **L-arginino-succinate** + AMP + diphosphate	M
3-DEHYDROQUINATE-SYNTHASE-RXN	3-dehydroquinate synthase	**3-deoxy-D-arabino-heptulosonate-7-phosphate** → **3-dehydroquinate** + phosphate	M
PRTRANS-RXN	Anthranilate phosphoribosyl transferase	**anthranilate** + **5-phosphoribosyl 1-pyrophosphate** → **N-(5'-phosphoribosyl)-anthranilate** + diphosphate	M
DIHYDROPICRED-RXN	Dihydrodi picolinate reductase	NAD(P)H + **L-2,3-dihydrodipicolinate** → NAD(P)+ + **tetrahydrodipicolinate**	M
ASPARTATE-SEMIALDEHYDE-DEHYDROGENASE-RXN	Aspartate-semialdehyde dehydrogenase	H+ + NADPH + **L-aspartyl-4-phosphate** → **L-aspartate-semialdehyde** + NADP+ + phosphate	M
ANTHRANSYN-RXN	Anthranilate synthase	L-glutamine + **chorismate** → **anthranilate** + **pyruvate** + L-glutamate	M
DIAMINOPIMDECARB-RXN	Diamino pimelate decarboxylase	**Meso-diaminopimelate** → **L-lysine** + CO_2	M
RXN0-2382	NA	**L-serine** + **indole** → **L-tryptophan** + H_2O	M
PRPPSYN-RXN	ribose-phosphate diphosphokinase	**D-ribose-5-phosphate** + ATP → **5-phosphoribosyl 1-pyrophosphate** + AMP	M
IGPSYN-RXN	indole-3-glycerol-phosphate synthase	**1-(o-carboxyphenylamino)-1'-deoxyribulose-5'-phosphate** → **indole-3-glycerol-phosphate** +H_2O +CO_2	M
SUCCDIAMINOPIMDESUCC-RXN	Succinyl-diaminopimelate desuccinylase	**N-succinyl-L,L-2,6-diaminopimelate** + H_2O → **succinate** + **L,L-diaminopimelate**	M
24654614619-RXN	3-phospho shikimate 1-carboxy vinyltransferase	**phosphoenolpyruvate** + **shikimate-3-phosphate** → **5-enolpyruvyl-shikimate-3-phosphate** + phosphate	M
BRANCHED-CHAINAMINOTRANSFERLEU-RXN	Branched-chain amino acid aminotransferase	2-ketoglutarate + **L-leucine** ← L-glutamate + **2-ketoisocaproate**	T
BRANCHED-CHAINAMINOTRANSFERILEU-RXN	Branched-chain amino acid aminotransferase	2-ketoglutarate + **L-isoleucine** ← L-glutamate + **2-keto-3-methyl-valerate**	T

Reaction BioCyc ID	Name	Formula	Evidence to assign the direction
DIHYDRODIPICSYN-RXN	Dihydro dipicolinate synthase	**pyruvate** + **L-aspartate-semialdehyde** → **L-2,3-dihydrodipicolinate** + H2O	M
DIHYDROXYMETVALDEHYDRAT-RXN	Dihydroxy-acid dehydratase	**2,3-dihydroxy-3-methylvalerate** → **2-keto-3-methyl-valerate** + H2O	M
THREDEHYD-RXN	Threonine dehydratase	**L-threonine** → ammonia + **2-oxobutanoate**	M
SHIKIMATE-KINASE-RXN	shikimate-kinase	ATP + **shikimate** → ADP + **shikimate-3-phosphate**	M
ARGSUCCINLYA-RXN	Arginino succinate lyase	**L-arginino-succinate** → **L-arginine** + **fumarate**	M
GAPOXNPHOSPHN-RXN	Glyceraldehyde 3-phosphate dehydrogenase (phosphorylating)	NAD+ + **D-glyceraldehyde-3-phosphate** + phosphate → NADH + **1,3-diphosphateglycerate**	T
ACETOOHBUTSYN-RXN	Acetolactate synthase	**pyruvate** + **2-oxobutanoate** → **2-aceto-2-hydroxy-butyrate** + CO2	M
PYRROLINECARBDEHYDROG-RXN	1-pyrroline-5-carboxylate dehydrogenase	**pyrroline 5-carboxylate** + NAD+ + H2O → NADH + **L-glutamate**	M
RXN-7933	NA	**N-acetyl-L-citrulline** + H2O → **acetate** + **citrulline**	M
ACETYLORNDEACET-RXN	Acetylornithine deacetylase	**N-acetyl-L-ornithine** + H2O → **acetate** + **L-ornithine**	M
