# Supplementary material for: Graph-Based Analysis of the Metabolic Exchanges between Two Co-Resident Intracellular Symbionts, Baumannia cicadellinicola and Sulcia muelleri, with Their Insect Host, Homalodisca coagulata
Source: PLoS Comput Biol. 2010 Sep 2;6(9):e1000904. doi: 10.1371/journal.pcbi.1000904 (PMC2936742; doi:10.1371/journal.pcbi.1000904)
Supplement: Table S4 — List of reactions in the metabolic network we build for B. cicadellinicola and the evidences used to assign the direction of the reactions. The metabolites kept in the reactions after filtering appear in bold. M: MetaCyc pathways evidence; T: topological evidence. (0.11 MB PDF) [file pcbi.1000904.s028.pdf]

Table S4. List of reactions in the metabolic network we build for *B. cicadellinicola* and the evidences used to assign the direction of the reactions. The metabolites kept in the reactions after filtering appear in bold. **M**: MetaCyc pathways evidence; **T**: topological evidence

Reaction BioCyc ID	Name	Formula	Evidence to assign the direction
RXNI-3	NA	menaquinone-8 + **malate** → menaquinol + **oxaloacetate**	M
6PGLUCONOLACT-RXN	6-phospho gluconolactonase	H2O + **D-glucono-delta-lactone-6-phosphate** → **6-phospho-D-gluconate**	M
DEPHOSPHOCOAKIN-RXN	Dephospho-CoA kinase	**dephospho-CoA** + ATP → ADP + **coenzyme A**	M
PGLUCISOM-RXN	Glucose-6-phosphate isomerase	**beta-D-glucose-6-phosphate** → **fructose-6-phosphate**	T
DIHYDROFOLATESYNTH-RXN	dihydrofolate synthetase	**7,8-dihydropteroate** + **L-glutamate** + ATP → ADP + phosphate + **7,8-dihydrofolate**	M
HOMOCYSMET-RXN	5-methyl tetrahydropteroyl triglutamate--homocysteine S-methyltransferase	**L-homocysteine** + 5-methyltetrahydropteroyltri-L-glutamate → **L-methionine** + tetrahydropteroyltri-L-glutamate	M
THI-P-SYN-RXN	Thiamine-phosphate pyro phosphorylase	**4-amino-5-hydroxymethyl-2-methylpyrimidine-pyrophosphate** + **4-methyl-5-(beta-hydroxyethyl)thiazole phosphate** → diphosphate + **thiamine-phosphate**	M
F16ALDOLASE-RXN	Fructose-bisphosphate aldolase	f**ructose-1,6-bisphosphate** → **dihydroxy-acetone phosphate** + **D-glyceraldehyde-3-phosphate**	T
UDPREDUCT-RXN	NA	**UDP** + a reduced thioredoxin → H2O + **dUDP** + an oxidized thioredoxin	M
METHYLENETHFDEHYDROG-NADP-RXN	Methylene tetrahydrofolate dehydrogenase (NADP+)	**5,10-methylene-THF** + NADP+ ↔ **5,10-methenyltetrahydrofolate** + NADPH	
GMP-SYN-NH3-RXN	GMP synthetase	ammonia + **xanthosine-5-phosphate** + ATP → **GMP** + diphosphate + AMP	M
RIBULP3EPIM-RXN	ribulose phosphate 3-epimerase	**D-ribulose-5-phosphate** → **D-xylulose-5-phosphate**	T
P-PANTOCYSLIG-RXN	Phospho pantothenate--cysteine ligase	**D-4'-phosphopantothenate** + **L-cysteine** + CTP → diphosphate + **R-4'-phosphopantothenoyl-L-cysteine** + CMP	M
GLUTRACE-RXN	Glutamate racemase	**L-glutamate** → **D-glutamate**	M
RIBOFLAVINKIN-RXN	Riboflavin kinase	ATP + **riboflavin** → **FMN** + ADP	M
3-DEHYDROQUINATE-DEHYDRATASE-RXN	3-dehydroquinate dehydratase	**3-dehydroquinate** → H2O + **3-dehydro-shikimate**	M
SIROHEME-FERROCHELAT-RXN	Sirohydrochlorin ferrochelatase	**sirohydrochlorin** + Fe2+ → H+ + **siroheme**	M

Reaction BioCyc ID	Name	Formula	Evidence to assign the direction
1.5.1.20-RXN	Methylene tetrahydrofolate reductase (NADPH)	H+ + **5,10-methylene-THF** + NAD(P)H → NAD(P)+ + **5-methyl-THF**	M
DIHYDROOROTOX-RXN	Dihydroorotate oxidase	**dihydroorotate** + oxygen → H2O2 + **orotate**	T
CHORISMATE-SYNTHASE-RXN	Chorismate synthase	**5-enolpyruvyl-shikimate-3-phosphate** → phosphate + **chorismate**	M
PHOSACETYLTRANS-RXN	Phosphate acetyltransferase	phosphate + **acetyl-CoA** ↔ coenzyme A + **acetylphosphate**	
CDPREDUCT-RXN	NA	a reduced thioredoxin + **CDP** → H2O + **dCDP** + an oxidized thioredoxin	M
PANTEPADENYLYLTRAN-RXN	Pantetheine-phosphate adenylyl transferase	**pantetheine 4'-phosphate** + **ATP** → **dephospho-CoA** + diphosphate	M
RXN-8972TER	UDP-N-acetylmuramoylalanyl-D-glutamate--2,6-diaminopimelate ligase	**UDP-N-acetylmuramoyl-L-alanyl-D-glutamate** + **meso-diaminopimelate** + ATP → **a UDP-N-acetylmuramoyl-tripeptide** + ADP + phosphate	M
7KAPSYN-RXN	8-amino-7-oxononanoate synthase	**L-alanine** + pimeloyl-CoA → **7-keto-8-aminopelargonate** + coenzyme A + CO2	M
H2PTEROATESYNTH-RXN	Dihydropteroate synthase	**(2-amino-4-hydroxy-7,8-dihydropteridin-6-yl)methyl diphosphate** + **p-aminobenzoate** → diphosphate + **7,8-dihydropteroate**	M
GPPSYN-RXN	Dimethyl allyltransferase	**dimethylallyl-diphosphate** + **isopentenyl diphosphate** → **geranyl-diphosphate** + diphosphate	M
5-FORMYL-THF-CYCLO-LIGASE-RXN	5-formyl tetrahydrofolate cyclo-ligase	**5-formyl-tetrahydrofolate** + ATP ↔ **5,10-methenyltetrahydrofolate** + ADP + phosphate	
POLY-RXN	polymerisation	**a UDP-N-acetylmuramoyl-pentapeptide** → **UDP-N-acetylmuramoyl-L-alanyl-D-glutamyl-meso-2,6-diaminoheptanedioate- D-alanyl-D-alanine**	M
TRANSALDOL-RXN	Transaldolase	**D-sedoheptulose-7-phosphate** + **D-glyceraldehyde-3-phosphate** →**D-erythrose-4-phosphate** + **fructose-6-phosphate**	T
QUINOLINATE-SYNTHA-RXN	NA	**dihydroxy-acetone phosphate** + **iminoaspartate** → H2O + phosphate + **quinolinate**	M
RXN-6102	NA	**L-glutamate** + **tetrahydrofolate** + ATP → **tetrahydrofolate-L-glutamate** + ADP + phosphate	M
PABSYNMULTI-RXN	synthesis of p-aminobenzoic acid	L-glutamine + **chorismate** → **pyruvate** + L-glutamate + **p-aminobenzoate** + H+	T
TRIOSEPISOMERIZATION-RXN	Triosephosphate isomerase	**D-glyceraldehyde-3-phosphate** ← **dihydroxy-acetone phosphate**	T

Reaction BioCyc ID	Name	Formula	Evidence to assign the direction
SAICARSYN-RXN	Phosphoribosyl aminoimidazole-succino carboxamide synthetase	**4-carboxyaminoimidazole ribonucleotide** + ATP + **L-aspartate** → ADP + phosphate + **5'-phosphoribosyl-4-(N-succinocarboxamide)-5-aminoimidazole**	M
RXN0-302	2-C-methyl-D-erythritol 2,4-cyclodiphosphate synthase	**2-phospho-4-(cytidine 5'-diphospho)-2-C-methyl-D-erythritol** → **2-C-methyl-D-erythritol-2,4-cyclodiphosphate** + **CMP**	M
ATPPHOSPHORIBOSYLTRANS-RXN	ATP phosphoribosyl transferase	**5-phosphoribosyl 1-pyrophosphate** + **ATP** → **phosphoribosyl-ATP** + diphosphate	M
PSERTRANSAMPYR-RXN	NA	**2-oxo-3-hydroxy-4-phosphobutanoate** + L-glutamate → **4-(phosphonooxy)-threonine** + 2-ketoglutarate	M
FADSYN-RXN	FMN adenylyl transferase	**FMN** + **ATP** → diphosphate + **FAD**	M
H2NEOPTERINALDOL-RXN	Dihydroneopterin aldolase	**7,8-dihydro-D-neopterin** → **6-hydroxymethyl-7,8-dihydropterin** + **glycolaldehyde**	M
PRPPAMIDOTRANS-RXN	Amido phosphoribosyl transferase	L-glutamine + H2O + **5-phosphoribosyl 1-pyrophosphate** → diphosphate + L-glutamate + **5-phospho-beta-D-ribosyl-amine**	M
4.2.3.12-RXN	6-pyruvoyl tetrahydropterin synthase	**7,8-dihydroneopterin triphosphate** → PPPi + **6-pyruvoyl tetrahydropterin**	M
PEPDEPHOS-RXN	Pyruvate kinase	ADP + **phosphoenolpyruvate** → **pyruvate** + ATP	M
DIMETHUROPORDEHYDROG-RXN	NA	NAD+ + **precorrin-2** → **sirohydrochlorin** + NADH	M
OROTPDECARB-RXN	Orotidine-5'-phosphate decarboxylase	**orotidine-5'-phosphate** → **UMP** + CO2	M
GDPKIN-RXN	NA	**GDP** + ATP → ADP + **GTP**	M
AICARSYN-RXN	Adenylosuccinate lyase	**5'-phosphoribosyl-4-(N-succinocarboxamide)-5-aminoimidazole** → **fumarate** + **aminoimidazole carboxamide ribonucleotide**	M
DIHYDROOROT-RXN	Dihydroorotase	**N-carbamoyl-L-aspartate** → **dihydroorotate** + H2O	M
PHOSNACMURPENTATRANS-RXN	Phospho-N-acetylmuramoyl-pentapeptide-transferase	**undecaprenyl phosphate** + **UDP-N-acetylmuramoyl-L-alanyl-D-glutamyl-meso-2,6-diaminoheptanedioate- D-alanyl-D-alanine** → **UMP** + **N-acetylmuramoyl-L-alanyl-D-glutamyl-meso-2,6-diaminoheptane-D-alanyl-D-alanine-diphosphoundecaprenol**	T
IMPCYCLOHYDROLASE-RXN	IMP cyclohydrolase	**phosphoribosyl-formamido-carboxamide** → **inosine-5'-phosphate** + H2O	M
GLU6PDEHYDROG-RXN	Glucose-6-phosphate 1-dehydrogenase	**beta-D-glucose-6-phosphate** + NADP+ → H+ + NADPH + **D-glucono-δ-lactone-6-phosphate**	M
UDPKIN-RXN	NA	**UDP** + ATP → ADP + **UTP**	M

Reaction BioCyc ID	Name	Formula	Evidence to assign the direction
PABASYN-RXN	Amino deoxychorismate synthase	L-glutamine + **chorismate** → **4-amino-4-deoxychorismate** + L-glutamate	M
P-PANTOCYSDECARB-RXN	Phospho pantothenoyl cysteine decarboxylase	**R-4'-phosphopantothenoyl-L-cysteine** → **pantetheine 4'-phosphate** + CO2	M
UMPKI-RXN	NA	**UMP** + ATP → **UDP** + ADP	M
H2PTERIDINEPYROPHOSPHOKIN-RXN	2-amino-4-hydroxy-6-hydroxy methyldihydro pteridine pyrophospho kinase	**6-hydroxymethyl-7,8-dihydropterin** + ATP → **(2-amino-4-hydroxy-7,8-dihydropteridin-6-yl)methyl diphosphate** + AMP	M
S-ADENMETSYN-RXN	Methionine adenosyl transferase	H2O + **L-methionine** + **ATP** → diphosphate + phosphate + **S-adenosyl-L-methionine**	M
2-DEHYDROPANTOATE-REDUCT-RXN	2-dehydropantoate 2-reductase	**2-dehydropantoate** + NADPH → **L-pantoate** + NADP+	M
HISTALDEHYD-RXN	Histidinol dehydrogenase	H2O + NAD+ + **histidinal** → NADH + **L-histidine**	M
GLUTATHIONE-SYN-RXN	glutathione synthetase	**L-γ-glutamylcysteine** + ATP + **glycine** → ADP + phosphate + **glutathione**	M
FGAMSYN-RXN	Phosphoribosyl formyl glycinamidine synthetase	L-glutamine + H2O + **5'-phosphoribosyl-N-formylglycineamide** + ATP → **5-phosphoribosyl-N-formylglycineamidine** + ADP + phosphate + L-glutamate	M
NAD-SYNTH-NH3-RXN	NAD(+) synthetase	ammonia + **deamido-NAD** + ATP → diphosphate + **NAD+** + AMP	M
GTP-CYCLOHYDRO-II-RXN	GTP cyclohydrolase II	H2O + **GTP** → **formate** + diphosphate + **2,5-diamino-6-(ribosylamino)-4-(3H)-pyrimidinone 5'-phosphate**	M
UDP-NACMURALA-GLU-LIG-RXN	UDP-N-acetylmuramoylalanine--D-glutamate ligase	**D-glutamate** + ATP + **UDP-N-acetylmuramoyl-L-alanine** → ADP + phosphate + **UDP-N-acetylmuramoyl-L-alanyl-D-glutamate**	M
PHOSPHASERDECARB-RXN	Phosphatidyl serine decarboxylase	**an L-1-phosphatidylserine** → **an L-1-phosphatidyl-ethanolamine** + CO2	M
THYMIDYLATESYN-RXN	Thymidylate synthase	5,10-methylene-THF + **dUMP** → **dTMP** + 7,8-dihydrofolate	T
L-ASPARTATE-OXID-RXN	NA	oxygen + **L-aspartate** → **H2O2** + **iminoaspartate**	M
DTDPKIN-RXN	NA	ATP + **dTDP** → **dTTP** + ADP	M

Reaction BioCyc ID	Name	Formula	Evidence to assign the direction
1.1.1.262-RXN	4-hydroxythreonine-4-phosphate dehydrogenase	**4-(phosphonooxy)-threonine** + NAD+ → NADH + **(2S)-2-amino-3-oxo-4-phosphonooxybutanoate**	M
PGPPHOSPHA-RXN	Phosphatidyl glycero phosphatase	H2O + **an L-1-phosphatidylglycerol-phosphate** → phosphate + **an L-1-phosphatidyl-glycerol**	M
ASPARTASE-RXN	Aspartate ammonia-lyase	**L-aspartate** → ammonia + **fumarate**	M
PDXJ-RXN	NA	**1-amino-propan-2-one-3-phosphate** + **1-deoxy-D-xylulose 5-phosphate** → **pyridoxine-5'-phosphate** + H2O + phosphate	M
CARDIOLIPSYN-RXN	NA	**an L-1-phosphatidyl-glycerol** → **glycerol** + **cardiolipin**	M
3-DEHYDROQUINATE-SYNTHASE-RXN	3-dehydroquinate synthase	**3-deoxy-D-arabino-heptulosonate-7-phosphate** → phosphate + **3-dehydroquinate**	M
GLUTAMIDOTRANS-RXN	NA	L-glutamine + **phosphoribulosylformimino-AICAR-P** → **D-erythro-imidazole-glycerol-phosphate** + L-glutamate + **aminoimidazole carboxamide ribonucleotide**	M
RIBOFLAVIN-SYN-RXN	Riboflavin synthase	**6,7-dimethyl-8-(1-D-ribityl)lumazine** → **riboflavin** + **5-amino-6-ribitylamino-2,4(1H,3H)-pyrimidinedione**	M
DUDPKIN-RXN	NA	**dUDP** + ATP → **dUTP** + ADP	M
RXN-8447	NA	H+ + **(2S)-2-amino-3-oxo-4-phosphonooxybutanoate** → **1-amino-propan-2-one-3-phosphate** + CO2	M
NAG1P-URIDYLTRANS-RXN	UDP-N-acetyl glucosamine pyropho sphorylase	**N-acetyl-glucosamine-1-phosphate** + **UTP** → diphosphate + **UDP-N-acetyl-D-glucosamine**	M
RXN0-2921	NA	**L-glutamate** + ATP + **a 5,10-methylene-tetrahydrofolate** → ADP + phosphate + **a 5,10-methylene-tetrahydrofolate**	M
ADENOSYLHOMOCYSTEINE-NUCLEOSIDASE-RXN	Adenosyl homocysteine nucleosidase	**S-adenosyl-L-homocysteine** + H2O → **S-ribosyl-L-homocysteine** + **adenine**	T
ACETATEKIN-RXN	Acetate kinase	**acetate** + ATP ↔ ADP + **acetylphosphate**	
ERYTH4PDEHYDROG-RXN	Erythrose-4-phosphate dehydrogenase	H2O + NAD+ + **D-erythrose-4-phosphate** → H+ + NADH + **erythronate-4-phosphate**	M
THI-P-KIN-RXN	Thiamine-phosphate kinase	ATP + **thiamine-phosphate** → ADP + **thiamine diphosphate**	M
DTMPKI-RXN	Thymidylate kinase	**dTMP** + ATP → ADP + **dTDP**	M
5.4.2.10-RXN	Phospho glucosamine mutase	**D-glucosamine-6-phosphate** → **D-glucosamine 1-phosphate**	M

Reaction BioCyc ID	Name	Formula	Evidence to assign the direction
RXN0-5180	NA	**isopentenyl diphosphate** + **(E,E)-farnesyl diphosphate** → **diphosphate** + **2-cis,6-trans,10-trans-geranylgeranyl diphosphate**	M
ASPCARBTRANS-RXN	Aspartate carbamoyl transferase	**carbamoyl-phosphate** + **L-aspartate** → phosphate + **N-carbamoyl-L-aspartate**	M
L-ASPARTATE-OXIDASE-RXN	L-aspartate oxidase	H2O + oxygen + **L-aspartate** → H2O2 + ammonia + **iminoaspartate**	T
ERYTHRON4PDEHYDROG-RXN	NA	NAD+ + **erythronate-4-phosphate** → **2-oxo-3-hydroxy-4-phosphobutanoate** + NADH	M
GARTRANSFORMYL2-RXN	NA	**formate** + **5-phospho-ribosyl-glycineamide** + ATP → **5'-phosphoribosyl-N-formylglycineamide** + ADP + phosphate	M
HISTAMINOTRANS-RXN	Histidinol-phosphate aminotransferase	**imidazole acetol-phosphate** + L-glutamate → **L-histidinol-phosphate** + 2-ketoglutarate	M
AIRCARBOXY-RXN	Phosphoribosyl aminoimidazole carboxylase	**5-aminoimidazole ribonucleotide** + CO2 → **4-carboxyaminoimidazole ribonucleotide**	M
DALADALALIG-RXN	D-alanine--D-alanine ligase	**D-alanine** + ATP → ADP + **D-alanyl-D-alanine** + phosphate	M
UDPNACETYLGLUCOSAMENOLPYRTRANS-RXN	UDP-N-acetyl glucosamine 1-carboxyvinyl transferase	**phosphoenolpyruvate** + **UDP-N-acetyl-D-glucosamine** → **UDP-GlcNAc-enolpyruvate** + phosphate	M
ADENYL-KIN-RXN	Adenylate kinase	**ATP** + **AMP** → **ADP**	M
CARBPSYN-RXN	carbamoyl-phosphate synthetase (glutamine-hydrolysing)	L-glutamine + H2O + **HCO3-** + ATP → **carbamoyl-phosphate** + ADP + phosphate + L-glutamate	M
NACGLCTRANS-RXN	Undecaprenyl diphospho-muramoyl pentapeptide beta-N- acetyl glucosaminyl transferase	**N-acetylmuramoyl-L-alanyl-D-glutamyl-meso-2,6-diaminoheptane-D-alanyl-D-alanine-diphosphoundecapreno**l + UDP-N-acetyl-D-glucosamine → UDP + **N-acetylmuramoyl-L-alanyl-D-glutamyl-meso-2,6-diaminoheptane-D-alanyl-D-alanine-diphosphoundecaprenyl-N-acetylglucosamine**	M
L-GLN-FRUCT-6-P-AMINOTRANS-RXN	Glucosamine--fructose-6-phosphate aminotransferase (isomerizing)	L-glutamine + **fructose-6-phosphate** → **D-glucosamine-6-phosphate** + L-glutamate	M
DXS-RXN	NA	**pyruvate** + **D-glyceraldehyde-3-phosphate** → **1-deoxy-D-xylulose 5-phosphate** + CO2	M
HEMEOSYN-RXN	heme o biosynthesis	H2O + **protoheme IX** + **(E,E)-farnesyl diphosphate** → diphosphate + **heme o**	T
GLYOHMETRANS-RXN	glycine hydroxy methyltransferase	**L-serine** + tetrahydrofolate ↔ H2O + 5,10-methylene-THF + **glycine**	

Reaction BioCyc ID	Name	Formula	Evidence to assign the direction
GLUTCYSLIG-RXN	Glutamate--cysteine ligase	**L-glutamate** + **L-cysteine** + ATP → ADP + **L-γ-glutamylcysteine** + phosphate	M
METBALT-RXN	NA	ammonia + **succinate** + **2-oxobutanoate** ← **O-succinyl-L-homoserine** + H2O	T
RXN-8675	Uro porphyrinogen-III C-methyltransferase	**precorrin-1** + S-adenosyl-L-methionine → S-adenosyl-L-homocysteine + **precorrin-2**	M
AMPSYN-RXN	Adenylosuccinate lyase	**adenylo-succinate** → **fumarate** + **AMP**	M
UROGENIIISYN-RXN	uroporphyrinogen-III synthase	**hydroxymethylbilane** → H2O + **uroporphyrinogen-III**	M
ADCLY-RXN	Amino deoxychorismate lyase	**4-amino-4-deoxychorismate** → **pyruvate** + **p-aminobenzoate**	M
GTP-CYCLOHYDRO-I-RXN	GTP cyclohydrolase I	H2O + **GTP** → **formate** + **7,8-dihydroneopterin triphosphate**	M
DIHYDROFOLATEREDUCT-RXN	Dihydrofolate reductase	NADPH + **7,8-dihydrofolate** → **tetrahydrofolate** + NADP+	M
2.7.7.60-RXN	2-C-methyl-D-erythritol 4-phosphate cytidylyl transferase	**2-C-methyl-D-erythritol-4-phosphate** + **CTP** → **4-(cytidine 5'-diphospho)-2-C-methyl-D-erythritol** + diphosphate	M
HOMSUCTRAN-RXN	Homoserine O-succinyl transferase	**homoserine** + succinyl-CoA → **O-succinyl-L-homoserine** + coenzyme A	M
DCTP-DEAM-RXN	dCTP deaminase	H2O + **dCTP** → ammonia + **dUTP**	M
PYRIMSYN3-RXN	Phospho methylpyrimidine kinase	**hydroxymethylpyrimidine phosphate** + ATP → ADP + **4-amino-5-hydroxymethyl-2-methylpyrimidine-pyrophosphate**	M
RXN-1381	NA	**sn-glycerol-3-phosphate** + a fatty acyl CoA → coenzyme A + **a 2-lysophosphatidate**	M
PEPCARBOXYKIN-RXN	Phospho enolpyruvate carboxykinase (ATP)	**oxaloacetate** + ATP → ADP + **phosphoenolpyruvate** + CO2	M
RIBOFLAVINSYNDEAM-RXN	NA	H2O + **2,5-diamino-6-(ribosylamino)-4-(3H)-pyrimidinone 5'-phosphate** → ammonia + **5-amino-6-(5'-phosphoribosylamino)uracil**	M
RXN-1623	NA	**a 2-lysophosphatidate** + a fatty acyl CoA → coenzyme A + **an L-phosphatidate**	M
THIAZOLSYN2-RXN	NA	L-tyrosine + a ThiS-ThiF acyl-disulfide + **1-deoxy-D-xylulose 5-phosphate** → a ThiS sulfur-carrier protein + 4-hydroxybenzyl alcohol + a ThiF protein + CO2 + **4-methyl-5-(beta-hydroxyethyl)thiazole phosphate**	M
DUTP-PYROP-RXN	dUTP pyrophosphatase	**dUTP** + H2O → diphosphate + **dUMP**	M

Reaction BioCyc ID	Name	Formula	Evidence to assign the direction
ACETYL-COA-CARBOXYLTRANSFER-RXN	Acetyl-CoA carboxylase	H+ + **HCO3-** + **acetyl-CoA** + ATP → **malonyl-CoA** + ADP + phosphate	M
DCDPKIN-RXN	NA	**dCDP** + ATP → ADP + **dCTP**	M
RXN-5985	NA	**9-mercaptodethiobiotin** → **biotin**	M
RXN-5984	NA	a sulfurated sulfur donor + **dethiobiotin** + a sulfur donor → **9-mercaptodethiobiotin** + an unsulfurated sulfur acceptor	M
DAHPSYN-RXN	2-dehydro-3-deoxyphospho heptonate aldolase	H2O + **phosphoenolpyruvate** + **D-erythrose-4-phosphate** → **3-deoxy-D-arabino-heptulosonate-7-phosphate** + phosphate	M
ADENYLOSUCCINATE-SYNTHASE-RXN	adenylosuccinate synthetase	**inosine-5'-phosphate** + GTP + **L-aspartate** → phosphate + GDP + **adenylo-succinate**	M
HISTPRATPHYD-RXN	Phosphoribosyl-ATP pyrophosphatase	H2O + **phosphoribosyl-ATP** → **phosphoribosyl-AMP** + diphosphate	M
PYRIMSYN1-RXN	NA	**5-aminoimidazole ribonucleotide** → **hydroxymethylpyrimidine phosphate**	M
PNPOXI-RXN	NA	**pyridoxine-5'-phosphate** + oxygen → H2O2 + **pyridoxal 5'-phosphate**	M
DETHIOBIOTIN-SYN-RXN	dethiobiotin synthetase	**7,8-diaminopelargonate** + ATP + CO2 → ADP + phosphate + **dethiobiotin**	M
IMIDPHOSDEHYD-RXN	Imidazoleglycerol-phosphate dehydratase	**D-erythro-imidazole-glycerol-phosphate** → **imidazole acetol-phosphate** + H2O	M
RXN-6282	NA	H2O + **5,10-methenyl-tetrahydropteroyl-[γ-Glu](n)** → **an N10-formyl-tetrahydrofolate**	M
PRPPSYN-RXN	ribose-phosphate diphosphokinase	**D-ribose-5-phosphate** + ATP → **5-phosphoribosyl 1-pyrophosphate** + AMP	M
GMP-SYN-GLUT-RXN	GMP synthetase (glutamine-hydrolysing)	L-glutamine + H2O + **xanthosine-5-phosphate** + ATP → **GMP** + diphosphate + L-glutamate + AMP	M
DAPASYN-RXN	Adenosylmethionine--8-amino-7-oxononanoate aminotransferase	**7-keto-8-aminopelargonate** + S-adenosyl-L-methionine → **7,8-diaminopelargonate** + S-adenosyl-4-methylthio-2-oxobutanoate	M
FPPSYN-RXN	Geranyl transtransferase	**geranyl-diphosphate** + **isopentenyl diphosphate** → diphosphate + **(E,E)-farnesyl diphosphate**	T
RXN-8001	NA	NAD+ + **histidinol** → H+ + NADH + **L-histidine**	T
PANTOATE-BETA-ALANINE-LIG-RXN	Pantoate--beta-alanine ligase	**beta-alanine** + ATP + **L-pantoate** → **pantothenate** + diphosphate + AMP	M
ACSERLY-RXN	Cysteine synthase	**O-acetyl-L-serine** + hydrogen sulfide → **acetate** + **L-cysteine**	M
O-SUCCHOMOSERLYASE-RXN	O-succinyl homoserine (thiol)-lyase	**O-succinyl-L-homoserine** + **L-cysteine** → **cystathionine** + **succinate**	M

**Reaction BioCyc ID**	**Name**	**Formula**	**Evidence to assign the direction**
CDPKIN-RXN	NA	**CDP** + ATP ↔ ADP + **CTP**	
RIB5PISOM-RXN	Ribose 5-phosphate epimerase	**D-ribose-5-phosphate** ← **D-ribulose-5-phosphate**	T
2.8.1.6-RXN	biotin synthase	a sulfurated sulfur donor + **dethiobiotin** + S-adenosyl-L-methionine + a sulfur donor → **biotin** + **5'-deoxyadenosine** + an unsulfurated sulfur acceptor + L-methionine	M
SERINE-O-ACETTRAN-RXN	Serine O-acetyltransferase	**L-serine** + acetyl-CoA → coenzyme A + **O-acetyl-L-serine**	M
QUINOPRIBOTRANS-RXN	Nicotinate-nucleotide pyro phosphorylase (carboxylating)	diphosphate + **nicotinate nucleotide** + CO2 ← **quinolinate** + **5-phosphoribosyl 1-pyrophosphate**	T
RIBOFLAVINSYNREDUC-RXN	NA	H+ + NADPH + **5-amino-6-(5'-phosphoribosylamino)uracil** → **5-amino-6-(5'-phosphoribitylamino)uracil** + NADP+	M
HISTOLDEHYD-RXN	Histidinol dehydrogenase	NAD+ + **histidinol** → **histidinal** + NADH	M
RXN-3742	NA	**L-glutamate** + ATP + **a folylpolyglutamate(n)** → ADP + phosphate + **a folylpolyglutamate(n)**	M
SHIKIMATE-KINASE-RXN	shikimate-kinase	ATP + **shikimate** → ADP + **shikimate-3-phosphate**	M
GLYC3PDEHYDROGBIOSYN-RXN	Glycerol-3-phosphate dehydrogenase (NAD(P)+)	H+ + **dihydroxy-acetone phosphate** + NAD(P)H → NAD(P)+ + **sn-glycerol-3-phosphate**	M
METHENYLTHFCYCLOHYDRO-RXN	Methenyl tetrahydrofolate cyclohydrolase	**5,10-methenyltetrahydrofolate** + H2O ↔ **10-formyl-tetrahydrofolate**	
PHOSGLYPHOS-RXN	Phosphoglycerate kinase	**3-phosphoglycerate** + ATP ← ADP + **1,3-diphosphateglycerate**	T
6PGLUCONDEHYDROG-RXN	Phosphogluconate dehydrogenase (decarboxylating)	NAD(P)+ + **6-phospho-D-gluconate** → **D-ribulose-5-phosphate** + NAD(P)H + CO2	M
2PGADEHYDRAT-RXN	Phosphopyruvate hydratase	**2-phosphoglycerate** → H2O + **phosphoenolpyruvate**	T
CTPSYN-RXN	CTP synthetase	L-glutamine + H2O + ATP + **UTP** → ADP + phosphate + L-glutamate + **CTP**	M
UROPORIIIMETHYLTRANSA-RXN	NA	**uroporphyrinogen-III** + S-adenosyl-L-methionine → S-adenosyl-L-homocysteine + **precorrin-1**	M
NAD-SYNTH-GLN-RXN	NAD(+) synthetase (glutamine-hydrolysing)	L-glutamine + H2O + **deamido-NAD** + ATP → diphosphate + **NAD+** + L-glutamate + AMP	M
FORMYLTHFGLUSYNTH-RXN	NA	**L-glutamate** + ATP + **an N10-formyl-tetrahydrofolate** → ADP + phosphate + **an N10-formyl-tetrahydrofolate**	M

Reaction BioCyc ID	Name	Formula	Evidence to assign the direction
HISTIDPHOS-RXN	Histidinol-phosphatase	H2O + **L-histidinol-phosphate** → phosphate + **histidinol**	M
1TRANSKETO-RXN	Transketolase	**D-xylulose-5-phosphate** + **D-ribose-5-phosphate** → **D-sedoheptulose-7-phosphate** + **D-glyceraldehyde-3-phosphate**	T
NAD-KIN-RXN	NAD(+) kinase	**NAD+** + ATP → ADP + **NADP+**	M
PHOSPHASERSYN-RXN	CDP-diacylglycerol--serine O-phosphatidyltransferase	**a CDP-diacylglycerol** + **L-serine** → **an L-1-phosphatidylserine** + **CMP**	M
GUANYL-KIN-RXN	Guanylate kinase	**GMP** + ATP → ADP + **GDP**	M
RXN0-5234	NA	**threonine** ↔ **acetaldehyde** + **glycine**	
RXN-8973	UDP-N-acetylmuramoylalanyl-D-glutamyl-2,6-diaminopimelate--D-alanyl- D-alanine ligase	**a UDP-N-acetylmuramoyl-tripeptide** + **D-alanyl-D-alanine** + ATP → ADP + phosphate + **a UDP-N-acetylmuramoyl-pentapeptide**	M
RXN-8972BIS	UDP-N-acetylmuramoyl alanyl-D-glutamate--2,6-diaminopimelate ligase	**L-lysine** + **UDP-N-acetylmuramoyl-L-alanyl-D-glutamate** + ATP → **a UDP-N-acetylmuramoyl-tripeptide** + ADP + phosphate	M
IMP-DEHYDROG-RXN	IMP dehydrogenase	**inosine-5'-phosphate** + H2O + NAD+ → **xanthosine-5-phosphate** + NADH	M
UNDECAPRENYL-DIPHOSPHATASE-RXN	Undecaprenyl-diphosphatase	H2O + **di-trans,poly-cis-undecaprenyl diphosphate** → phosphate + **undecaprenyl phosphate**	M
RXN0-5240	NA	**D-alanine** + pyridoxal 5'-phosphate → **pyruvate** + pyridoxamine 5'-phosphate	T
DIOHBUTANONEPSYN-RXN	NA	**D-ribulose-5-phosphate** → **formate** + **3,4-dihydroxy-2-butanone-4-P**	M
DGDPKIN-RXN	NA	**dGDP** + ATP → **dGTP** + ADP	M
3PGAREARR-RXN	Phosphoglycerate mutase	**3-phosphoglycerate** → **2-phosphoglycerate**	T
SHIKIMATE-5-DEHYDROGENASE-RXN	Shikimate 5-dehydrogenase	**3-dehydro-shikimate** + NADPH → **shikimate** + NADP+	M
NICONUCADENYLYLTRAN-RXN	Nicotinate-nucleotide adenylyl transferase	**nicotinate nucleotide** + **ATP** → diphosphate + **deamido-NAD**	M
DIAMINOPIMEPIM-RXN	Diaminopimelate epimerase	**L,L-diaminopimelate** → **meso-diaminopimelate**	M
GLYRIBONUCSYN-RXN	Phosphoribosyl amine--glycine ligase	ATP + **glycine** + **5-phospho-beta-D-ribosyl-amine** → ADP + phosphate + **5-phospho-ribosyl-glycineamide**	M

Reaction BioCyc ID	Name	Formula	Evidence to assign the direction
CDPDIGLYSYN-RXN	phosphatidate cytidylyl transferase	**an L-phosphatidate** + **CTP** → **a CDP-diacylglycerol** + diphosphate	M
RXN-6321	NA	**5,10-methenyltetrahydrofolate** + H2O → **5-formyl-tetrahydrofolate**	M
UDPNACETYLMURAMATEDEHYDROG-RXN	UDP-N-acetylmuramate dehydrogenase	NADPH + **UDP-GlcNAc-enolpyruvate** → **UDP-N-acetylmuramate** + NADP+	M
FUMHYDR-RXN	Fumarate hydratase	**Malate** ← H2O + **fumarate**	T
OHMETHYLBILANESYN-RXN	Hydroxy methylbilane synthase	H2O + **porphobilinogen** → ammonia + **hydroxymethylbilane**	M
RXN-8998	NA	**di-trans,poly-cis-nonaprenyl diphosphate** + **isopentenyl diphosphate** → **di-trans,poly-cis-decaprenyl diphosphate** + diphosphate	M
RXN-8997	NA	**isopentenyl diphosphate** + **di-trans,poly-cis-octaprenyl diphosphate** → **di-trans,poly-cis-nonaprenyl diphosphate** + diphosphate	M
RXN-8999	NA	**di-trans,poly-cis-decaprenyl diphosphate** + **isopentenyl diphosphate** → diphosphate + **di-trans,poly-cis-undecaprenyl diphosphate**	M
RXN-8994	NA	**isopentenyl diphosphate** + **di-trans,poly-cis-pentaprenyl diphosphate** → diphosphate + **di-trans,poly-cis-hexaprenyl diphosphate**	M
PRIBFAICARPISOM-RXN	N-(5'-phospho-D-ribosylformimino)-5-amino-1-(5''-phosphoribosyl)-4- imidazole carboxamide isomerase	**phosphoribosylformiminoAICAR-phosphate** → **phosphoribulosylformimino-AICAR-P**	M
RXN-8993	NA	**isopentenyl diphosphate** + **2-cis,6-trans,10-trans-geranylgeranyl diphosphate** → diphosphate + **di-trans,poly-cis-pentaprenyl diphosphate**	M
RXN-8996	NA	**isopentenyl diphosphate** + **di-trans,poly-cis-heptaprenyl diphosphate** → diphosphate + **di-trans,poly-cis-octaprenyl diphosphate**	M
6PFRUCTPHOS-RXN	6-phospho fructokinase	**fructose-6-phosphate** + ATP → ADP + **fructose-1,6-bisphosphate**	M
CYSTATHIONINE-BETA-LYASE-RXN	Cystathionine beta-lyase	**cystathionine** + H2O → ammonia + **pyruvate** + **L-homocysteine**	M
RXN-8995	NA	**isopentenyl diphosphate** + **di-trans,poly-cis-hexaprenyl diphosphate** → diphosphate + **di-trans,poly-cis-heptaprenyl diphosphate**	M
CMPKI-RXN	Cytidylate kinase	ATP + **CMP** → ADP + **CDP**	M
2.3.1.157-RXN	Glucosamine-1-phosphate N-acetyltransferase	acetyl-CoA + **D-glucosamine 1-phosphate** → coenzyme A + **N-acetyl-glucosamine-1-phosphate**	M

Reaction BioCyc ID	Name	Formula	Evidence to assign the direction
PHOSPHAGLYPSYN-RXN	CDP-diacylglycerol--glycerol-3-phosphate 3-phosphatidyl transferase	**a CDP-diacylglycerol** + **sn-glycerol-3-phosphate** → **an L-1-phosphatidylglycerol-phosphate** + **CMP**	M
ISPH2-RXN	NA	H+ + **1-hydroxy-2-methyl-2-(E)-butenyl 4-diphosphate** + NAD(P)H → NAD(P)+ + H2O + **isopentenyl diphosphate**	M
AIRS-RXN	Phosphoribosyl formyl glycinamidine cyclo-ligase	**5-phosphoribosyl-N-formylglycineamidine** + ATP → ADP + phosphate + **5-aminoimidazole ribonucleotide**	M
OROPRIBTRANS-RXN	Orotate phosphoribosyl transferase	**orotate** + **5-phosphoribosyl 1-pyrophosphate** → diphosphate + **orotidine-5'-phosphate**	M
2.5.1.19-RXN	3-phosphoshikimate 1-carboxyvinyl transferase	**phosphoenolpyruvate** + **shikimate-3-phosphate** → phosphate + **5-enolpyruvyl-shikimate-3-phosphate**	M
PYRUVDEH-RXN	NA	coenzyme A + NAD+ + **pyruvate** → NADH + **acetyl-CoA** + CO2	M
FOLYLPOLYGLUTAMATESYNTH-RXN	Folyl polyglutamate synthetase	**L-glutamate** + ATP + **a tetrahydrofolate polyglutamate** → ADP + phosphate + **a tetrahydrofolate polyglutamate**	M
PANTOTHENATE-KIN-RXN	Pantothenate kinase	**pantothenate** + ATP → **D-4'-phosphopantothenate** + ADP	M
AICARTRANSFORM-RXN	Phosphoribosyl aminoimidazole carboxamide formyltransferase	10-formyl-tetrahydrofolate + **aminoimidazole carboxamide ribonucleotide** → **phosphoribosyl-formamido-carboxamide** + tetrahydrofolate	M
GDPREDUCT-RXN	NA	**GDP** + a reduced thioredoxin → **dGDP** + H2O + an oxidized thioredoxin	M
RXN0-884	NA	H+ + **1-hydroxy-2-methyl-2-(E)-butenyl 4-diphosphate** + NAD(P)H → NAD(P)+ + H2O + **dimethylallyl-diphosphate**	M
DXPREDISOM-RXN	NA	NADPH + **1-deoxy-D-xylulose 5-phosphate** → **2-C-methyl-D-erythritol-4-phosphate** + NADP+	M
RXN0-882	NA	**2-C-methyl-D-erythritol-2,4-cyclodiphosphate** + a protein dithiol → H2O + **1-hydroxy-2-methyl-2-(E)-butenyl 4-diphosphate** + a protein disulfide	M
2TRANSKETO-RXN	Transketolase	**D-xylulose-5-phosphate** + **D-erythrose-4-phosphate** → **D-glyceraldehyde-3-phosphate** + **fructose-6-phosphate**	T
GART-RXN	Phosphoribosyl glycinamide formyltransferase	**5-phospho-ribosyl-glycineamide** + 10-formyl-tetrahydrofolate → **5'-phosphoribosyl-N-formylglycineamide** + tetrahydrofolate	M

Reaction BioCyc ID	Name	Formula	Evidence to assign the direction
2.7.1.148-RXN	4-(cytidine 5'-diphospho)-2-C-methyl-D-erythritol kinase	**4-(cytidine 5'-diphospho)-2-C-methyl-D-erythritol** + ATP → ADP + **2-phospho-4-(cytidine 5'-diphospho)-2-C-methyl-D-erythritol**	M
LUMAZINESYN-RXN	NA	**3,4-dihydroxy-2-butanone-4-P** + **5-amino-6-ribitylamino-2,4(1H,3H)-pyrimidinedione** → H2O + phosphate + **6,7-dimethyl-8-(1-D-ribityl)lumazine**	M
3-CH3-2-OXOBUTANOATE-OH-CH3-XFER-RXN	3-methyl-2-oxobutanoate hydroxy methyltransferase	H2O + 5,10-methylene-THF + **2-keto-isovalerate** → **2-dehydropantoate** + tetrahydrofolate	M
GAPOXNPHOSPHN-RXN	Glyceraldehyde 3-phosphate dehydrogenase (phosphorylating)	phosphate + NAD+ + **D-glyceraldehyde-3-phosphate** → NADH + **1,3-diphosphateglycerate**	T
HISTCYCLOHYD-RXN	Phosphoribosyl-AMP cyclohydrolase	H2O + **phosphoribosyl-AMP** → **phosphoribosylformiminoAICAR-phosphate**	M
RXN0-742	NA	HCO3- + **5-aminoimidazole ribonucleotide** + ATP → ADP + phosphate + **N5-carboxyaminoimidazole ribonucleotide**	M
UDP-NACMUR-ALA-LIG-RXN	UDP-N-acetylmuramate--alanine ligase	**UDP-N-acetylmuramate + L-alanine** + ATP → ADP + phosphate + **UDP-N-acetylmuramoyl-L-alanine**	M
RXN0-743	NA	**N5-carboxyaminoimidazole ribonucleotide** → **4-carboxyaminoimidazole ribonucleotide**	M
SULITE-REDUCT-RXN	Sulfite reductase (NADPH)	sulfite → hydrogen sulfide	M
HYDROGENSULFITE__45__REDUCTASE__45__RXN	Hydrogen sulfite reductase	bisulfite → trithionate + OH-	M
RXN__45__8315	NA	sulfite → bisulfite	M
ADENYLYLSULFKIN__45__RXN	Adenylyl sulfate kinase	adenosine 5'-phosphosulfate → phosphoadenosine-5'-phosphosulfate	M
_1__46__8__46__4__46__8__45__RXN	Phospho adenylyl-sulfate reductase (thioredoxin)	phosphoadenosine-5'-phosphosulfate → adenosine-3',5'-bisphosphate + sulfite	M
ADENYLYLSULFKIN__45__RXN	Adenylyl sulfate kinase	adenosine 5'-phosphosulfate → phosphoadenosine-5'-phosphosulfate	M
SULFATE__45__ADENYLYLTRANS__45__RXN	Sulfate adenylyl transferase	sulfate + ATP → adenosine 5'-phosphosulfate	M
_325__45__BISPHOSPHATE__45__NUCLEOTIDASE__45__RXN	3'(2'),5'-bisphosphate nucleotidase	adenosine-3',5'-bisphosphate → AMP	M
SUPEROX__45__DISMUT__45__RXN	Superoxide dismutase	O2- → H2O2	M
RXN0__45__1241	NA	L-glutamine → N5-methyl-L-glutamine	M

Reaction BioCyc ID	Name	Formula	Evidence to assign the direction
PMPOXI__45__RXN	Pyridoxamine-phosphate oxidase	pyridoxamine 5'-phosphate → H_2O_2 + pyridoxal 5'-phosphate	M
