# Supplementary material for: Graph-Based Analysis of the Metabolic Exchanges between Two Co-Resident Intracellular Symbionts, Baumannia cicadellinicola and Sulcia muelleri, with Their Insect Host, Homalodisca coagulata
Source: PLoS Comput Biol. 2010 Sep 2;6(9):e1000904. doi: 10.1371/journal.pcbi.1000904 (PMC2936742; doi:10.1371/journal.pcbi.1000904)
Supplement: Table S5 — List of transformations cofactors used to automatically filter the metabolic networks of B. cicadellinicola and of S. muelleri. Each line corresponds to a transformation of cofactors. If the metabolites written in bold in the first column appear in a side of a reaction and the metabolites written in bold in the second column appear in the other side, they and the corresponding subproducts (written in normal font) are removed in the reaction. The third column and the fourth column indicate the number of affected reactions in the metabolic networks of S. muelleri and B. cicadellinicola. (0.05 MB PDF) [file pcbi.1000904.s029.pdf]

Table S5. List of transformations cofactors used to automatically filter the metabolic networks of *B. cicadellinicola* and of *S. muelleri*. Each line corresponds to a transformation of cofactors. If the metabolites written in bold in the first column appear in a side of a reaction and the metabolites written in bold in the second column appear in the other side, they and the corresponding subproducts (written in normal font) are removed in the reaction.The third column and the fourth column indicate the number of affected reactions in the metabolic networks of *S. muelleri* and *B. cicadellinicola*.

**Side 1**	**Side 2**	**Number of reactions in *S. muelleri***	**Number of reactions in *B. cicadellinicola***
**ATP**	**ADP** + Pi	5	51
**NADH**	**NAD+** + H+	3	10
**NADPH**	**NADP+** + H+	5	9
**ATP**	**AMP** + diphosphate	2	7
**NAD(P)H**	**NAD(P)+** + H+	3	5
**An oxidized thioredoxin**	**A reduced thioredoxin**	0	4
**Coenzyme A**	**Acetyl-CoA**	2	4
**S-adenosyl-L-methionine**	**S-adenosyl-L-homocysteine**	1	3
**L-glutamate**	**2-ketoglutarate**	8	2
**L-glutamate**	**glutamine**	2	10
**Coenzyme A**	**A fatty acyl CoA**	0	2
**GTP**	**GDP** + phosphate	0	2
**Tetrahydrofolate**	**10-formyl-tetrahydrofolate**	0	2
**Tetrahydrofolate**	**5,10-methylene-THF**	0	2
**UTP**	**UDP** + phosphate	0	1
**UDP**	**UDP-N-acetyl-D-glucosamine**	0	1
**An oxidized electron acceptor**	**A reduced electron acceptor**	0	1
**CTP**	**CDP** + phosphate	0	1
**CTP**	**CMP** + diphosphate	0	1
**Coenzyme A**	**Pimeloyl-CoA**	0	1
**Coenzyme A**	**Succinyl-CoA**	1	1
**7,8-dihydrofolate**	**5,10-methylene-THF**	0	1
**A protein dithiol**	**A protein disuldide**	0	1
