# Supplementary material for: Graph-Based Analysis of the Metabolic Exchanges between Two Co-Resident Intracellular Symbionts, Baumannia cicadellinicola and Sulcia muelleri, with Their Insect Host, Homalodisca coagulata
Source: PLoS Comput Biol. 2010 Sep 2;6(9):e1000904. doi: 10.1371/journal.pcbi.1000904 (PMC2936742; doi:10.1371/journal.pcbi.1000904)

Figure S1. Sub-network corresponding to the production of leucine and valine from erythrose-4-phosphate and phosphoenolpyruvate in *Sulcia muelleri*.Squares correspond to reactions and circles to metabolites. The colour of the edges differenciates the two sides of a reaction.

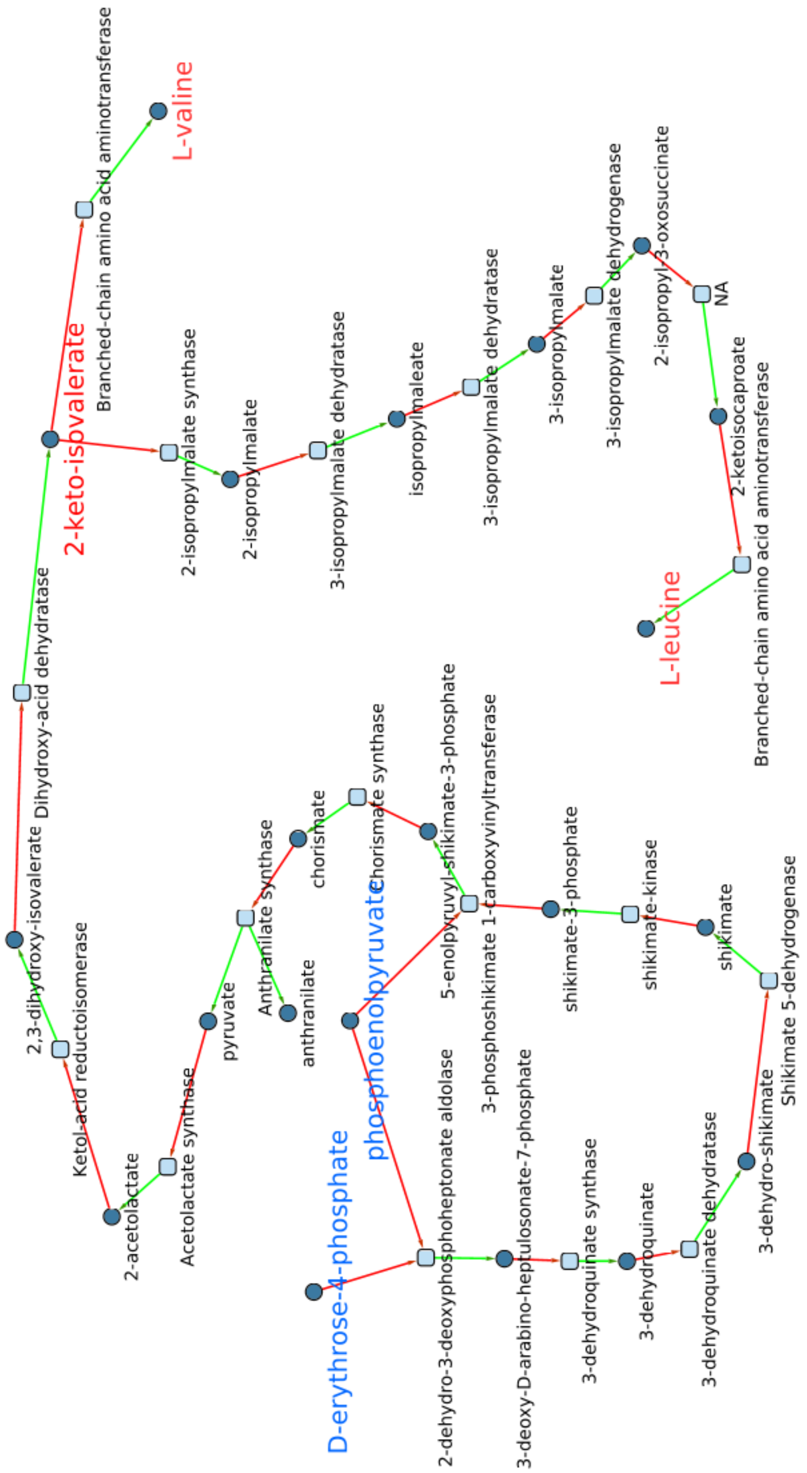

Figure S2. Sub-network corresponding to the production of lysine, homoserine and isoleucine from erythrose-4-phosphate, oxaloacetate, aspartate and phosphoenolpyruvate in *Sulcia muelleri*.Squares correspond to reactions and circles to metabolites. The colour of the edges differenciates the two sides of a reaction.

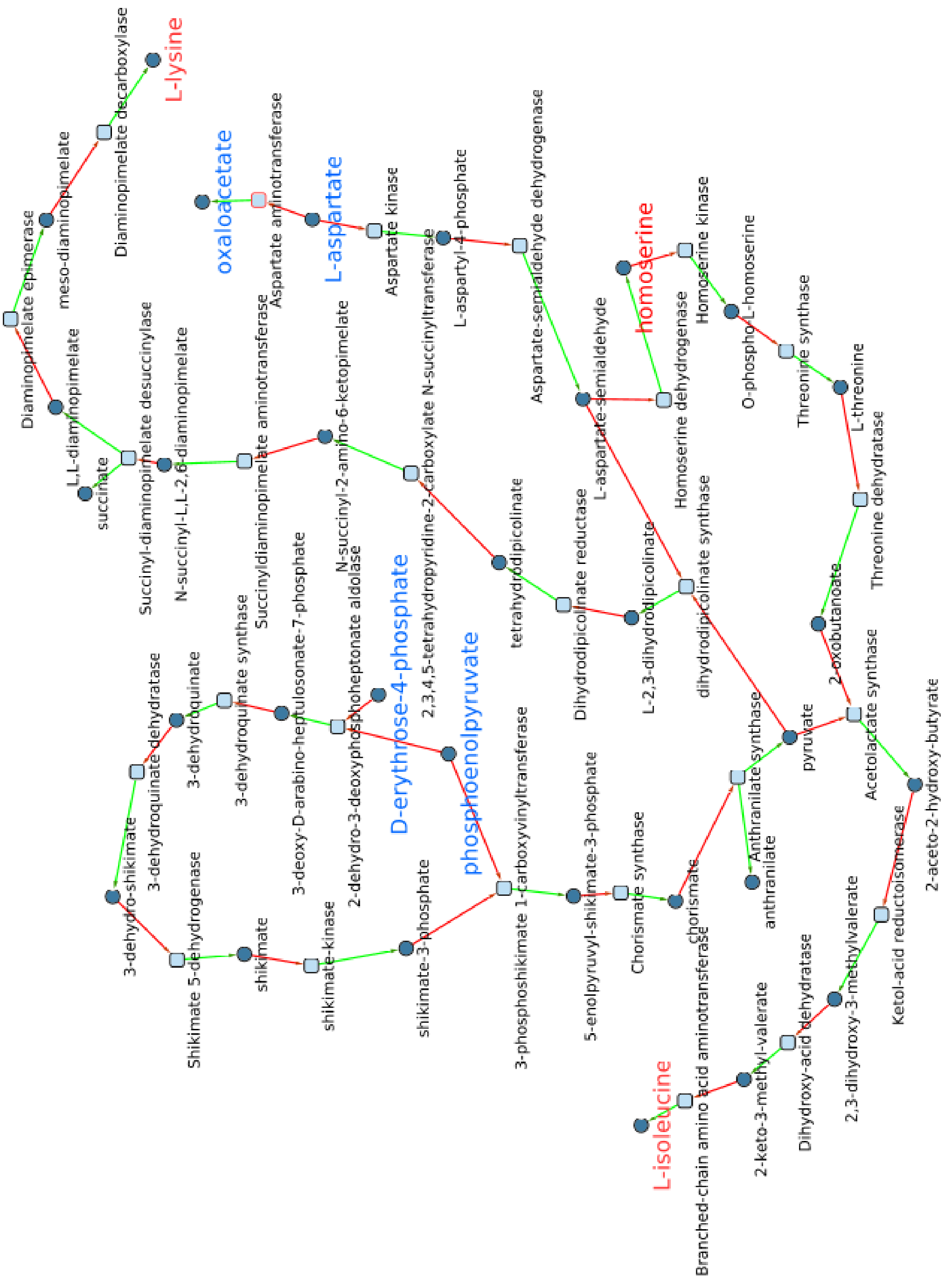

Figure S3. Sub-network corresponding to the production of phenylalanine from erythrose-4-phosphate and phosphoenolpyruvate in *Sulcia muelleri*.Squares correspond to reactions and circles to metabolites. The colour of the edges differenciates the two sides of a reaction.

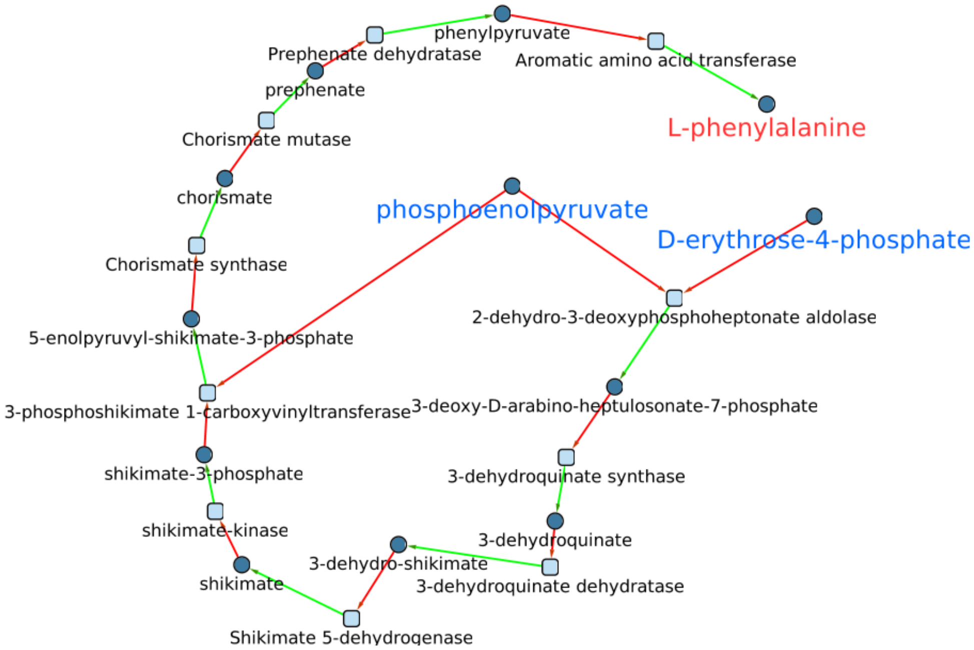

Figure S4. Sub-network corresponding to the production of tryptophane from erythrose-4-phosphate, ribose-5-phosphate, serine and phosphoenolpyruvate in *Sulcia muelleri*.Squares correspond to reactions and circles to metabolites. The colour of the edges differenciates the two sides of a reaction.

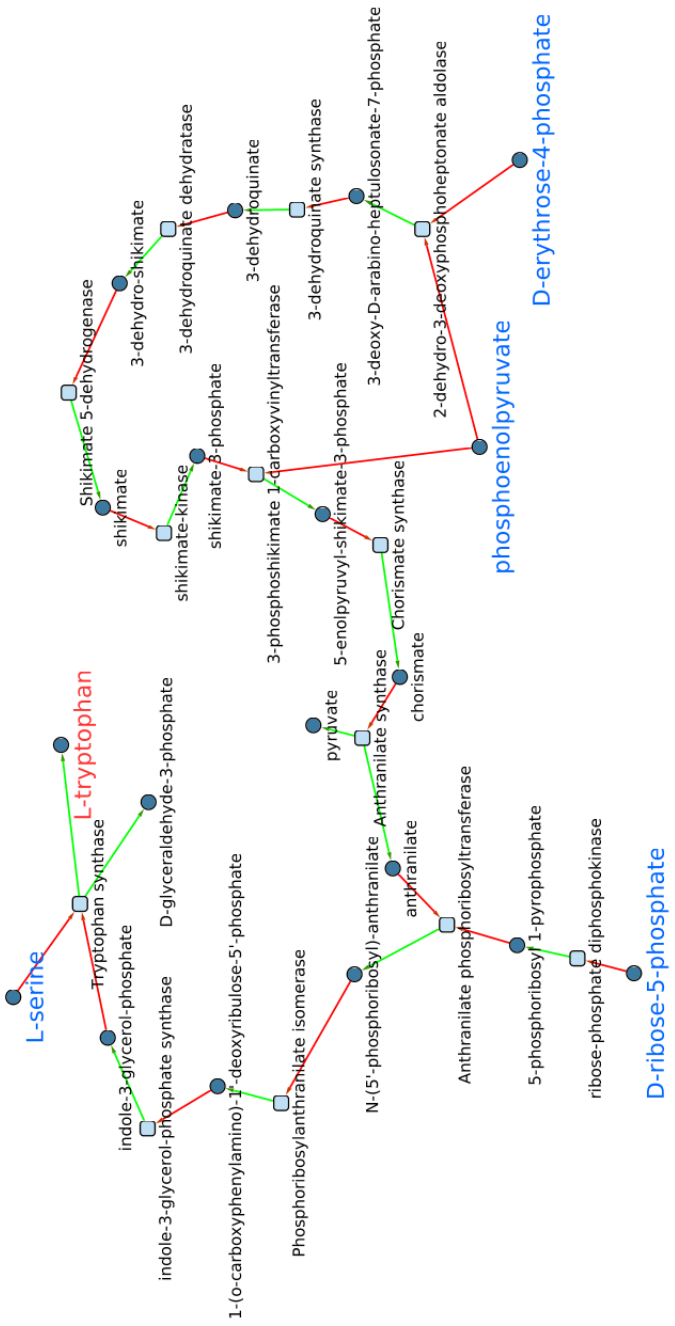

Figure S5. Sub-network corresponding to the production of menaquinone from octaprenyl-diphosphate and 1,4-dihydroxy-2-naphthoate in *Sulcia muelleri*.Squares correspond to reactions and circles to metabolites. The colour of the edges differenciates the two sides of a reaction.

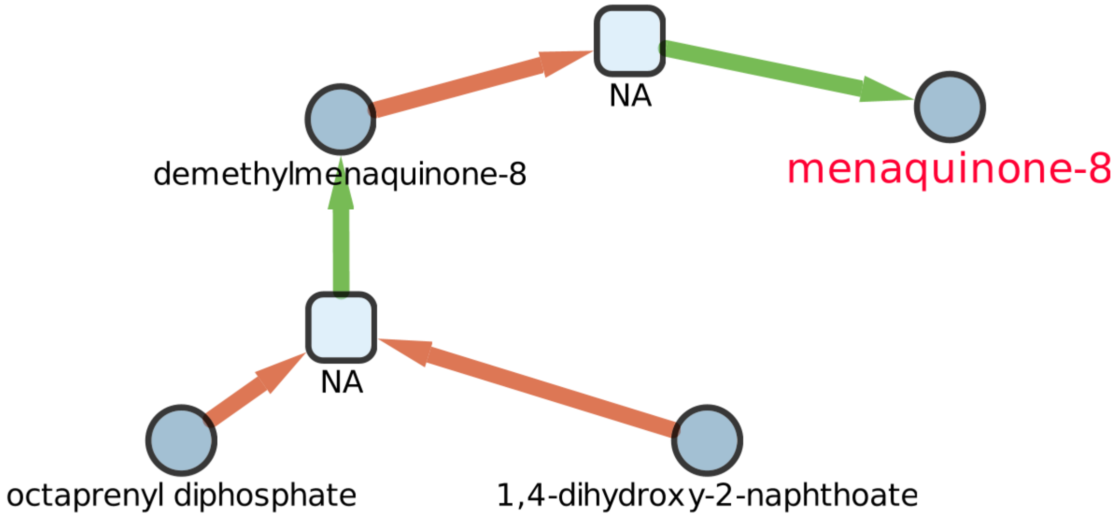

Figure S6. Sub-network corresponding to the production of histidine from glucose, and aspartate in *B. cicadellinicola*. Squares correspond to reactions and circles to metabolites. The colour of the edges differenciates the two sides of a reaction.

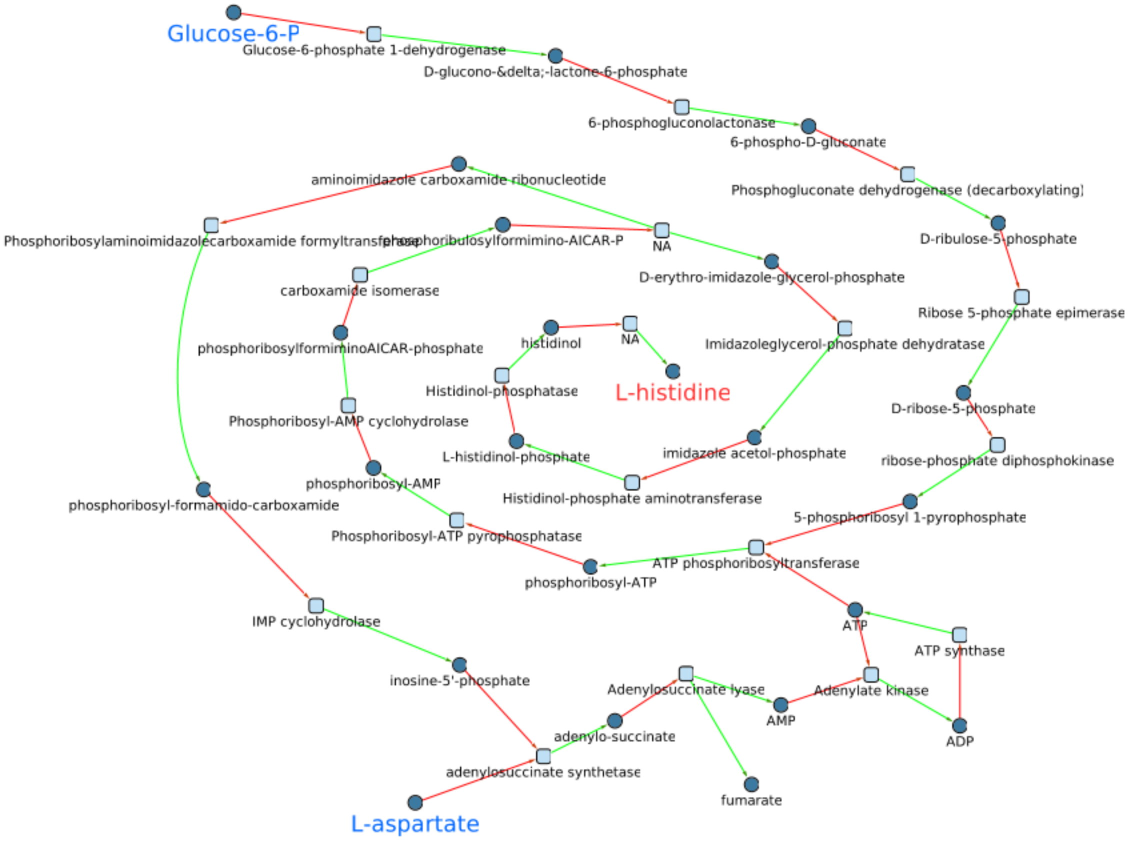

Figure S7. Sub-network corresponding to the production of methionine from serine, glycine, threonine and homoserine in *B. cicadellinicola*. Squares correspond to reactions and circles to metabolites. The colour of the edges differenciates the two sides of a reaction.

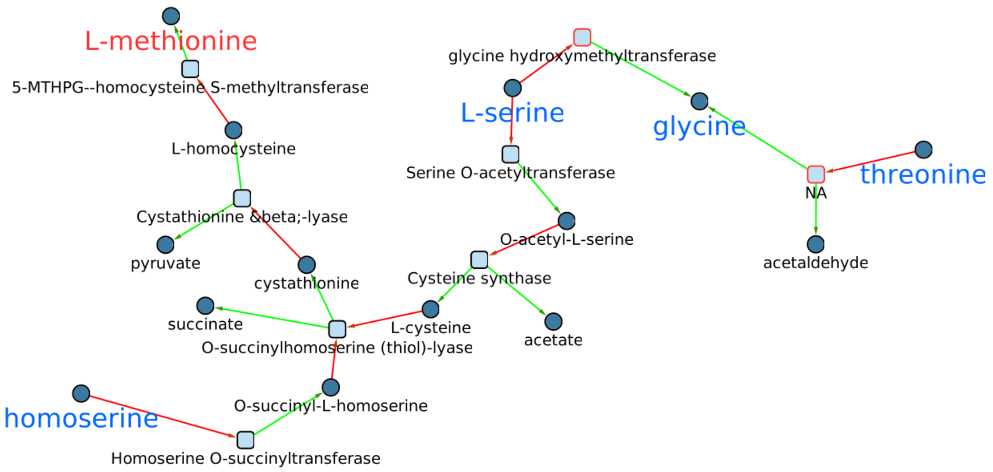

Figure S8. Sub-network corresponding to the production of co-enzyme A from β-alanine, glucose, aspartate, glycine, serine, threonine and keto-isovalerate in *B. cicadellinicola*. Squares correspond to reactions and circles to metabolites. The colour of the edges differenciates the two sides of a reaction.

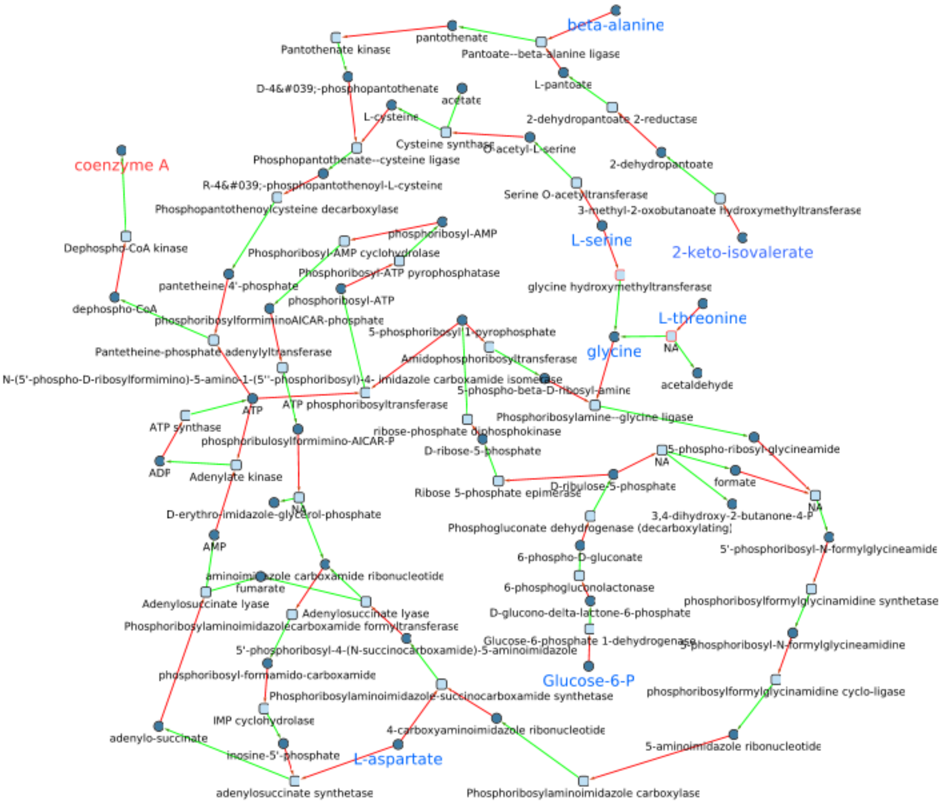

Figure S9. Sub-network corresponding to the production of biotin from alanine in *B. cicadellinicola*. Squares correspond to reactions and circles to metabolites. The colour of the edges differenciates the two sides of a reaction.

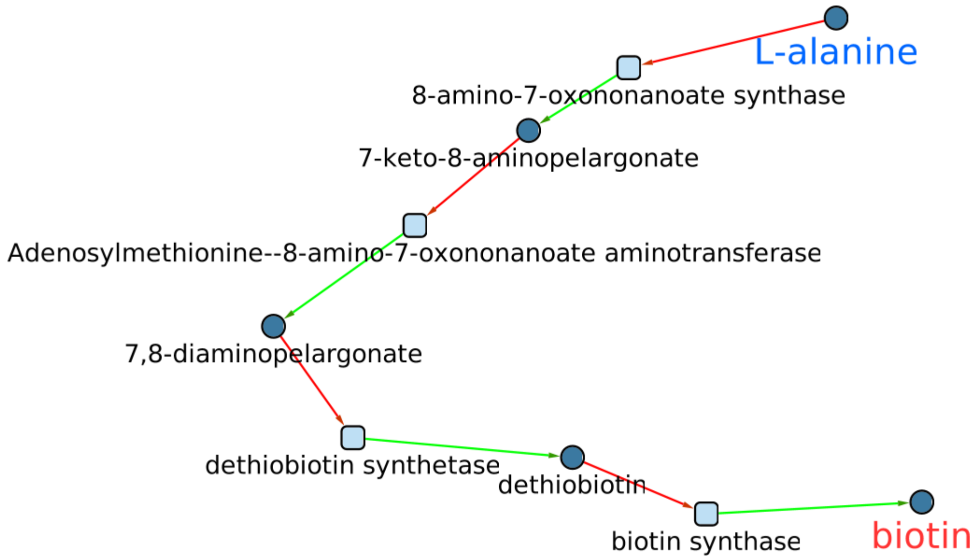

Figure S10. Sub-network corresponding to the production of pyridoxal-5'-phosphate from glucose in *B. cicadellinicola*. Squares correspond to reactions and circles to metabolites. The colour of the edges differenciates the two sides of a reaction.

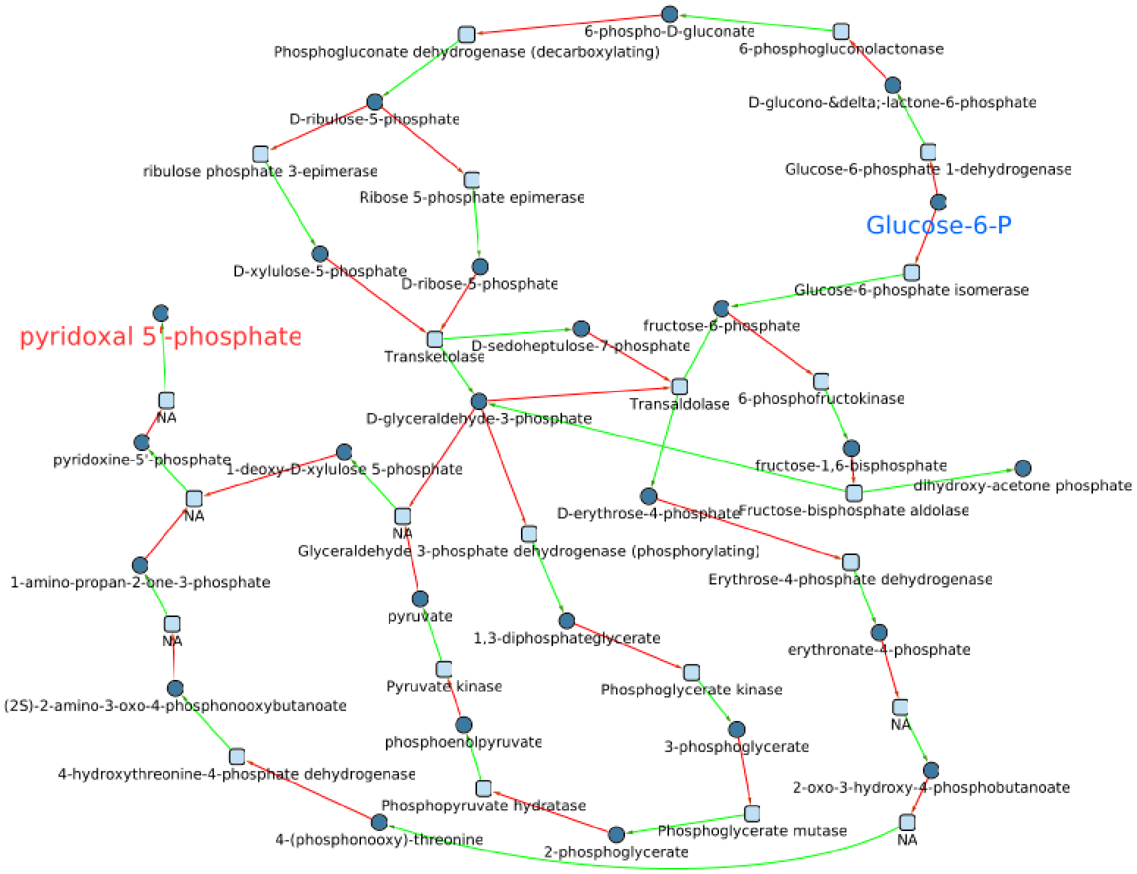

Figure S11. Sub-network corresponding to the production of riboflavin from glucose in *B. cicadellinicola*. Squares correspond to reactions and circles to metabolites. The colour of the edges differenciates the two sides of a reaction.

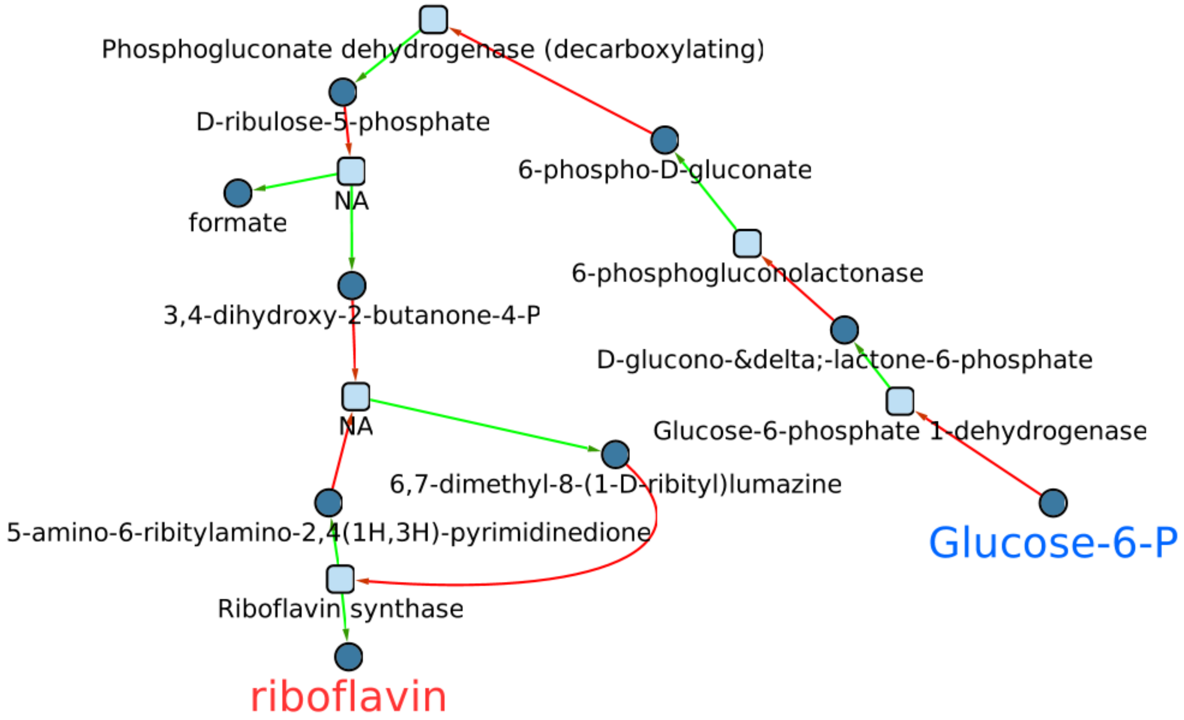

Figure S12. Sub-network corresponding to the production of glutathione from glutamate, glycine, serine and threonine in *B. cicadellinicola*. Squares correspond to reactions and circles to metabolites. The colour of the edges differenciates the two sides of a reaction.

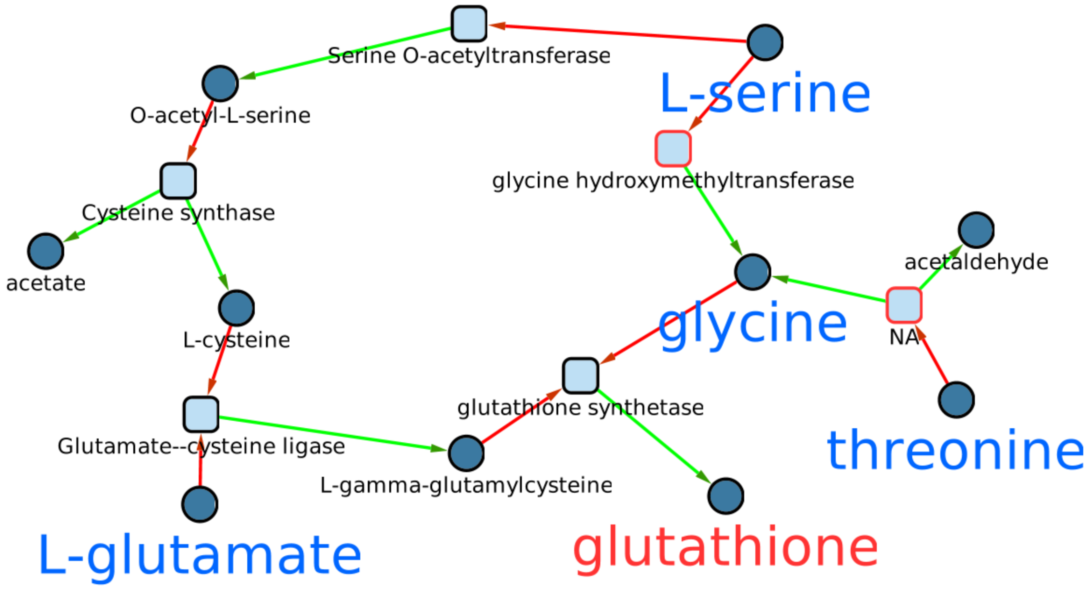

Figure S13. Sub-network corresponding to the production of thiamine from glucose, serine, glycine, tyrosine and threonine in *B. cicadellinicola*. Squares correspond to reactions and circles to metabolites. The colour of the edges differenciates the two sides of a reaction.

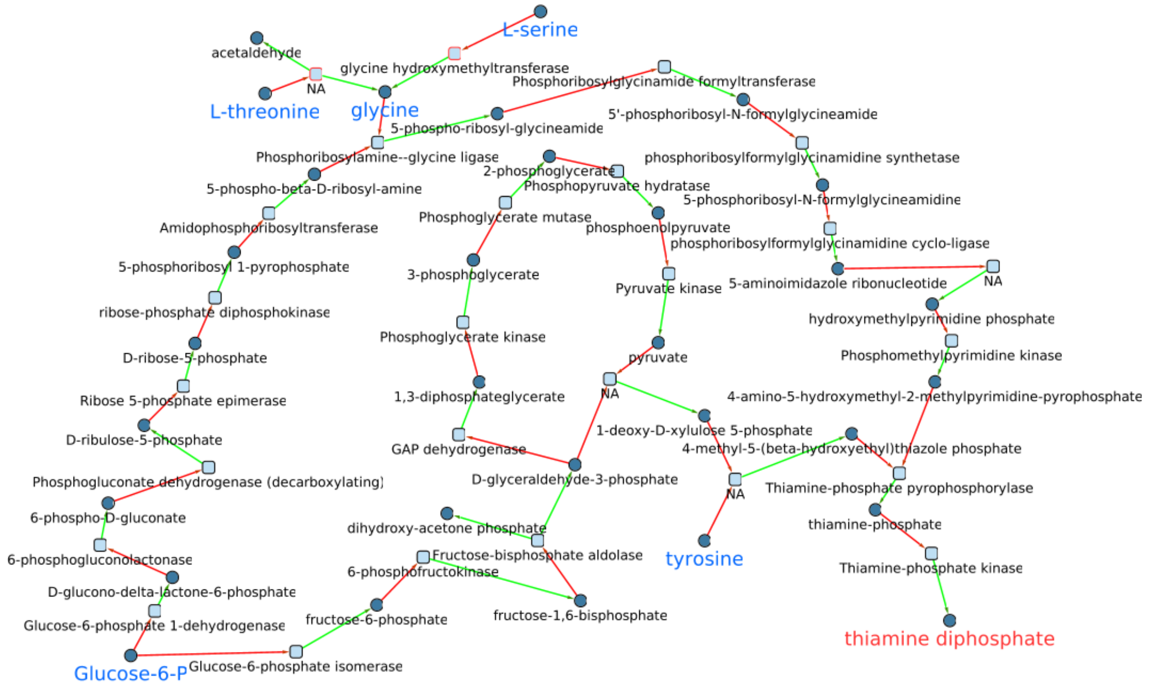

Figure S14. Sub-network corresponding to the production of tetrahydrofolate from dihydroneopterin, glucose and glutamate in *B. cicadellinicola*. Squares correspond to reactions and circles to metabolites. The colour of the edges differenciates the two sides of a reaction.

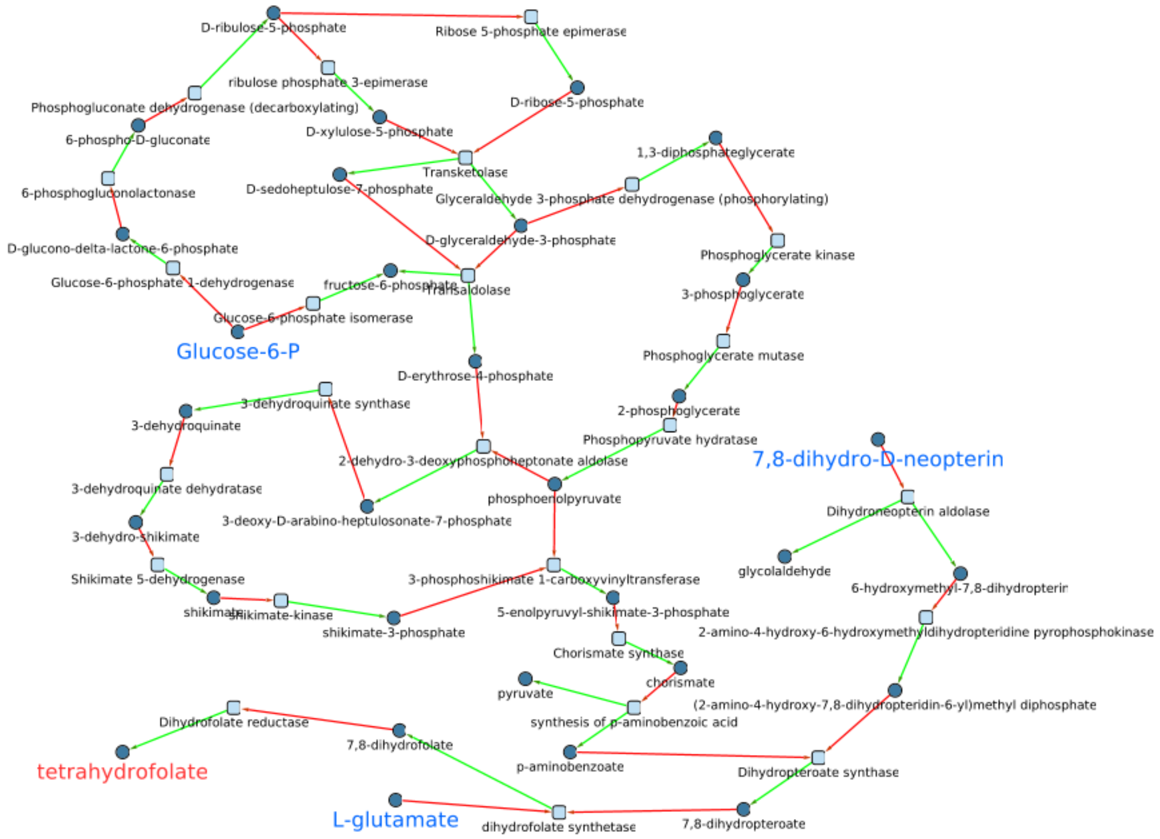

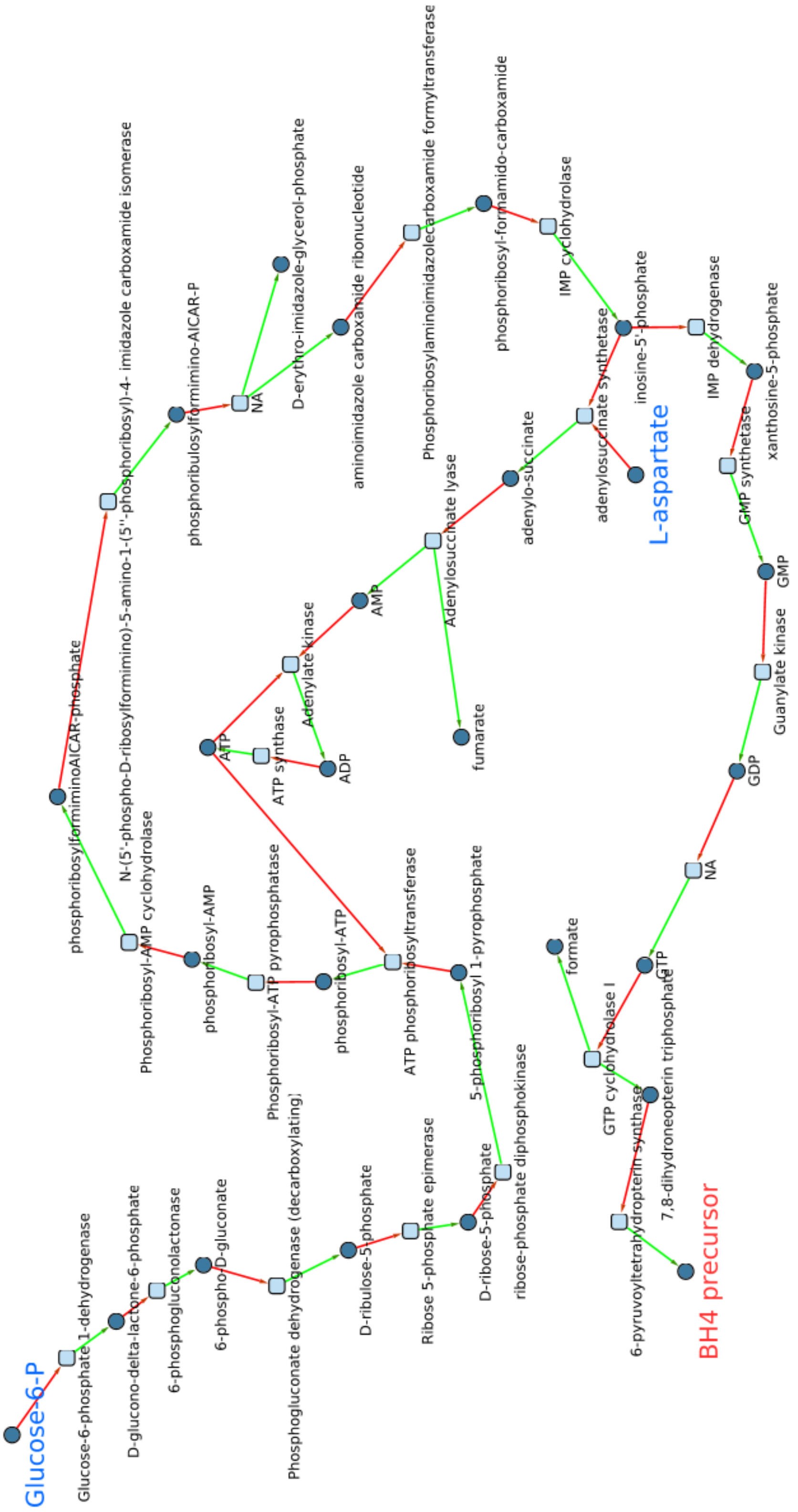

Figure S15. Sub-network corresponding to the production of BH4 precursor from glucose and aspartate in *B. cicadellinicola*. Squares correspond to reactions and circles to metabolites. The colour of the edges differenciates the two sides of a reaction.

Figure S16. Sub-network corresponding to the production of heme from glucose and protoheme in *B. cicadellinicola*. Squares correspond to reactions and circles to metabolites. The colour of the edges differenciates the two sides of a reaction.

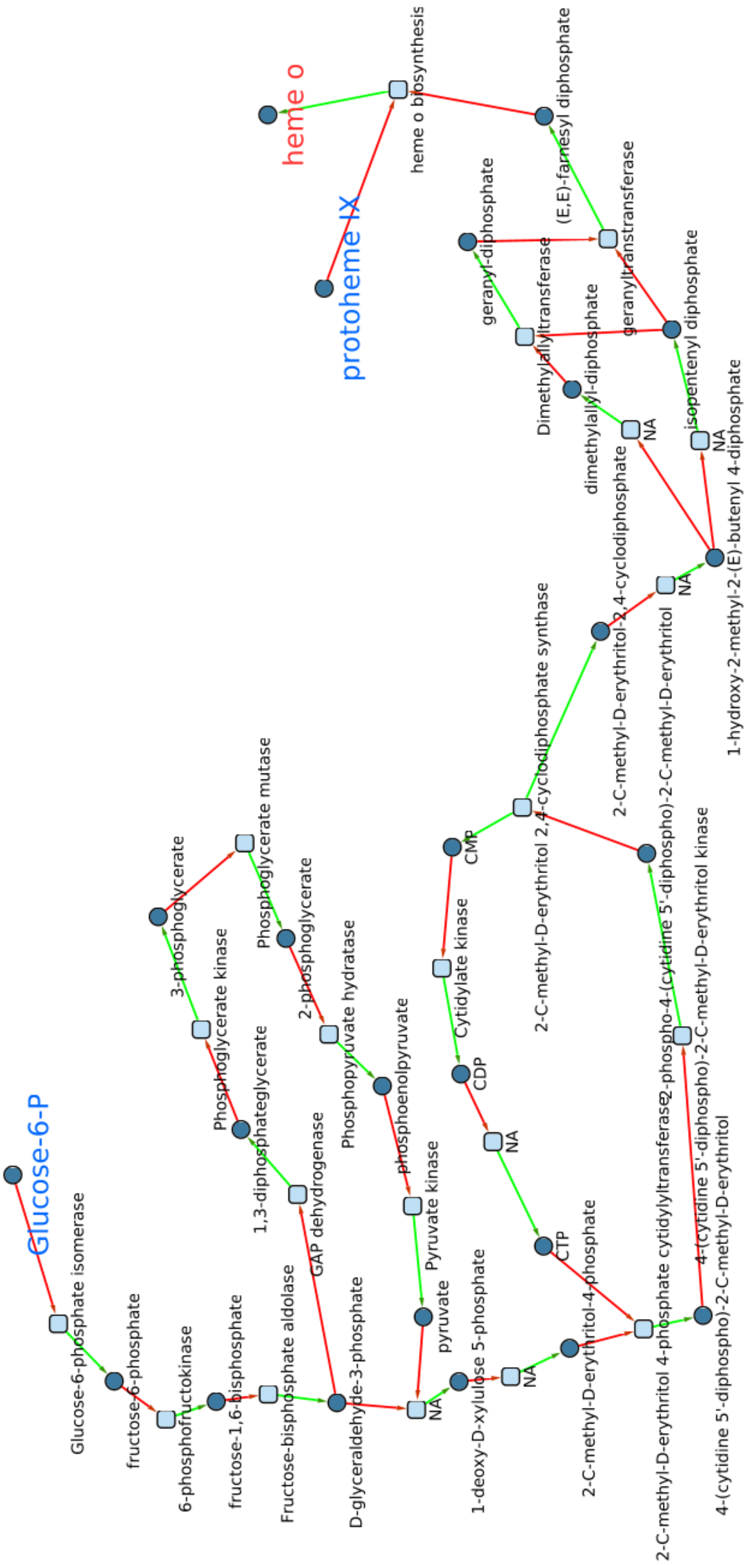

Figure S17. Sub-network corresponding to the production of erythrose-4-phosphate and of ribose-5-phosphate from glucose in *B. cicadellinicola.* Squares correspond to reactions and circles to metabolites. The colour of the edges differenciates the two sides of a reaction.

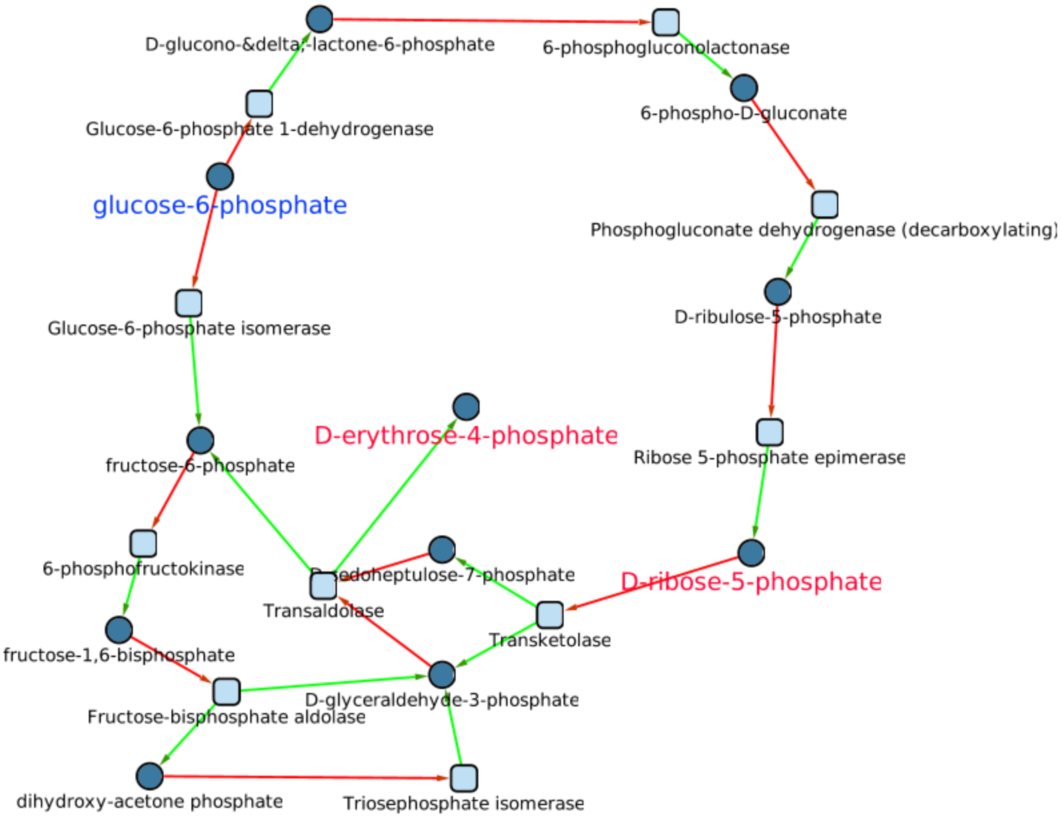

Figure S18. Sub-network corresponding to the production of oxaloacetate from aspartate in *B. cicadellinicola*. Squares correspond to reactions and circles to metabolites. The colour of the edges differenciates the two sides of a reaction.

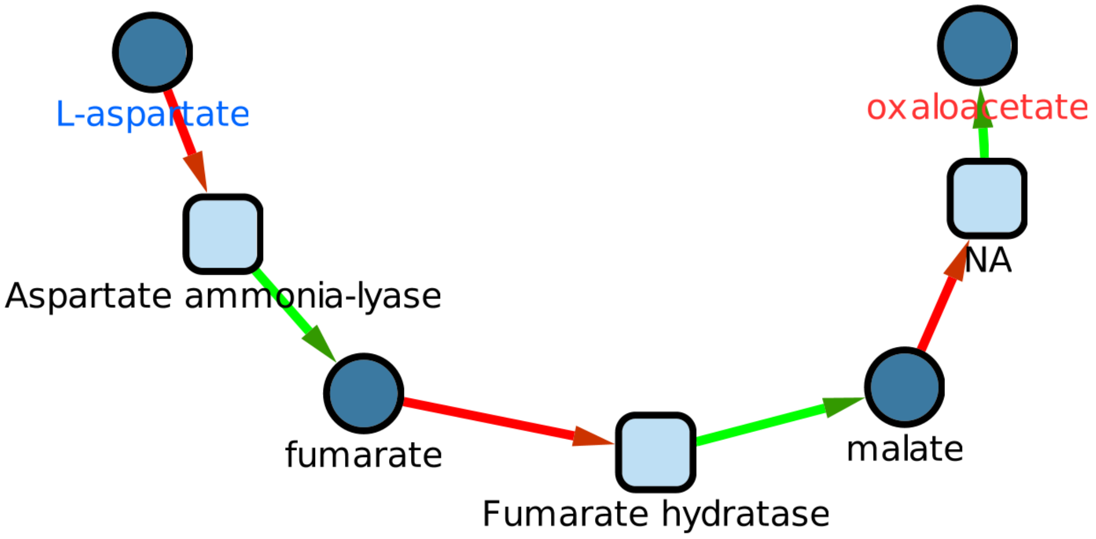

Figure S19. Sub-network corresponding to the production of phosphoenolpyruvate from glucose or aspartate in *B. cicadellinicola*. Squares correspond to reactions and circles to metabolites. The colour of the edges differenciates the two sides of a reaction.

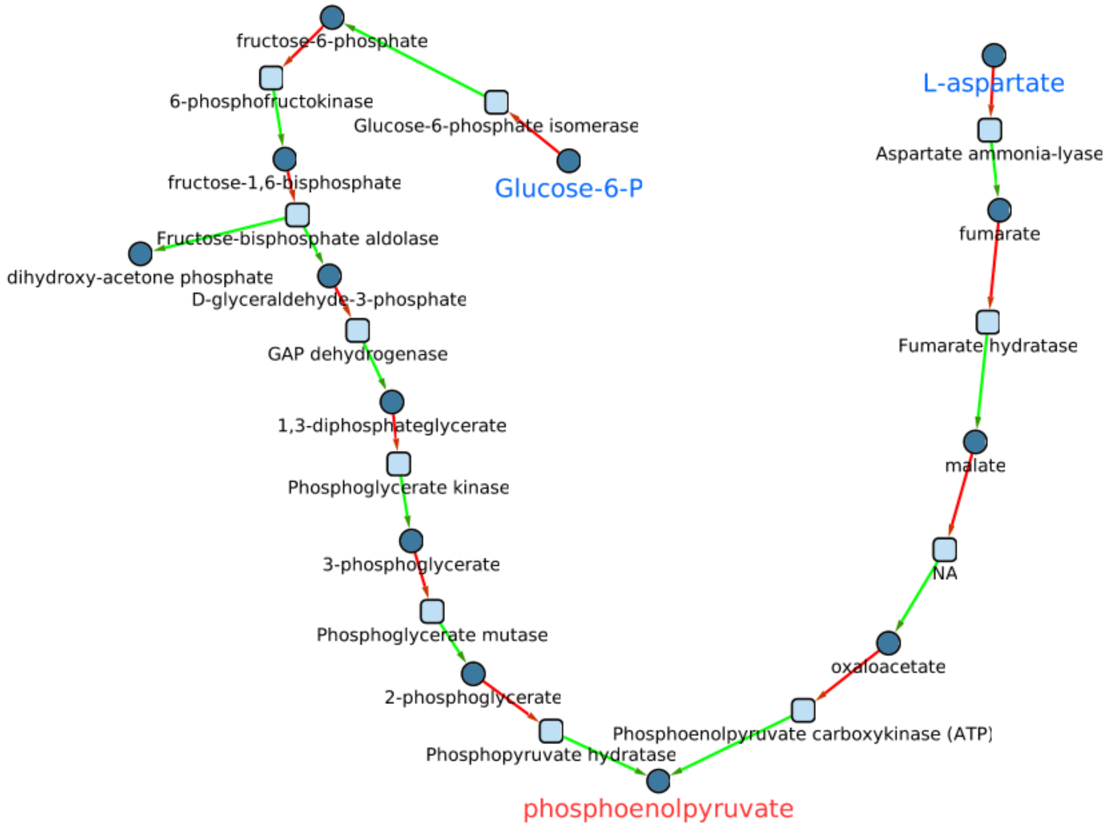

Figure S20. Sub-network corresponding to the production of octaprenyl-diphosphate from glucose in *B. cicadellinicola*. Squares correspond to reactions and circles to metabolites. The colour of the edges differenciates the two sides of a reaction.

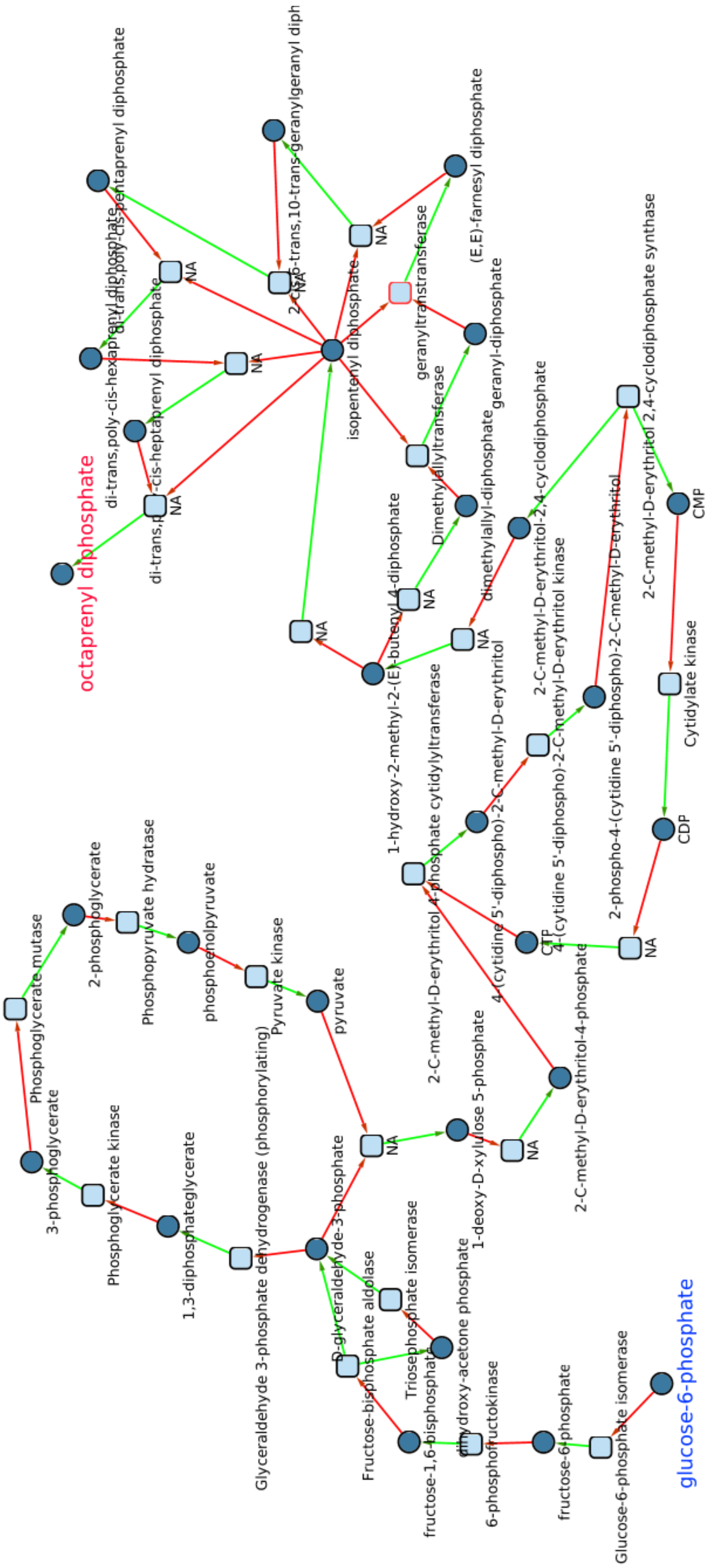

Supplement: Text S1 — Sub-networks linking each target metabolite to their precursor sets. (2.11 MB PDF) [file pcbi.1000904.s030.pdf]
